# Supplementary material for: PAMAM-Calix-Dendrimers: Third Generation Synthesis and Impact of Generation and Macrocyclic Core Conformation on Hemotoxicity and Calf Thymus DNA Binding
Source: Pharmaceutics. 2024 Oct 27;16(11):1379. doi: 10.3390/pharmaceutics16111379 (PMC11597237; doi:10.3390/pharmaceutics16111379)
Supplement: Supplementary file 1 [file pharmaceutics-16-01379-s001.zip › pharmaceutics-3241002-supplementary.pdf]

pharmaceutics

Electronic Supplementary Information

PAMAM-Calix-Dendrimers: Third Generation Synthesis and Impact of Generation and Macrocyclic Core Conformation on Hemotoxicity and Calf Thymus DNA Binding

Olga Mostovaya [1], Igor Shiabiev [1], Daniil Ovchinnikov [1], Dmitry Pysin [1], Timur Mukhametzyanov [1], Alesia Stanavaya [2], Viktar Abashkin [2], Dzmitry Shcharbin [2], Arthur Khannanov [1], Marianna Kutyreva [1], Mingwu Shen [3], Xiangyang Shi [3,4], Pavel Padnya [1,*] and Ivan Stoikov [1,*]

1. Materials and Methods **2**

2. NMR, IR and mass spectra **8**

3. Complexation investigation **19**

3.1. UV-Vis spectra **19**

3.2. Fluorescence spectroscopy data **24**

3.3. DLS data **37**

3.4. TEM data **52**

4. References **54**

1. Materials and Methods

1.1. General experimental information

^{1}H NMR and $^{13}C\{^{1}H\}$ NMR spectra were obtained on the Bruker Avance-400 spectrometer (Bruker Corp., Billerica, MA, USA) (^{1}H 400 MHz and $^{13}C\{^{1}H\}$ 100 MHz). Chemical shifts were determined against the signals of residual protons of deuterated solvent (CD_3OD). Concentrations of the compounds were equal to 3–5 mass %. FTIR ATR spectra were recorded on the Spectrum 400 FT-IR spectrometer (Perkin–Elmer, Seer Green, Llantrisant, UK) with the Diamond KRS-5 attenuated total internal reflectance attachment (resolution 0.5 cm^{-1}, accumulation of 64 scans, recording time 16 s in the wavelength range 400–4000 cm^{-1}). Melting points were determined using the Boetius Block apparatus (VEB Kombinat Nagema, Radebeul, Germany). High-resolution mass spectra with electrospray ionization (ESI HRMS) were obtained on an Agilent iFunnel 6550 Q-TOF LC/MS (Agilent Technologies, Santa Clara, CA, USA) in positive mode, equipped with Agilent 1290 Infinity II LC. Elution solvent used was 0.1% formic acid in mixture of Milli-Q water (20%) and HPLC-grade acetonitrile (80%). ESI conditions: carrier gas-nitrogen, temperature 300 °C, carrier flow rate 0.2 L × min^{-1}, nebulizer pressure 275 kPa, funnel voltage 3500 V, capillary voltage 500 V, total ion current recording mode, 100–3000 m/z mass range.

All reagents and solvents were used directly as purchased or purified according to the standard procedures. Methyl acrylate, ethylenediamine, and ninhydrin were purchased from Acros Organics (Geel, Belgium), sodium salt of calf thymus DNA, Tris-HCl, and ethidium bromide were purchased from Sigma-Aldrich (St. Louis, MO, USA). Deionized water with resistivity >18.0 MΩ cm (Millipore-Q, Simplicity® water purification system, Merck-Millipore, Molsheim, France) was used for the solutions preparation.

The first (**G1-cone**, **G1-paco**, and **G1-alt**) and second (**G2-cone**, **G2-paco**, and **G2-alt**) generations of **PAMAM-calix-dendrimers** were synthesized according to the previously described procedures [1,2].

1.2. General procedure for the synthesis of G2.5 PAMAM-calix-dendrimers.

The solution (10% by the weight) of **G2** (0.40 g, 0.098 mmol) in methanol was added dropwise to an ice-cooled (0 °C) methyl acrylate (0.56 mL, 6.27 mmol) dissolved in 4 mL of methanol. It was kept in the ice bath for 2 h. Then, the reaction mixture was stirred at room temperature for 48 h. Afterward, methyl acrylate and the solvent were removed on a rotary evaporator. The residue was dried under reduced pressure.

5,11,17,23-Tetra-*tert*-butyl-25,26,27,28-tetrakis[*N*-(6-(*N*,*N*-di(*N*-(2-(*N*,*N*-di(*N*-(2-(*N*,*N*-di(methoxycarbonylethyl)amino)ethyl)carbamoylethyl)amino)ethyl)carbamoylethyl)amino)hexyl)carbamoylmethoxy]-2,8,14,20-tetrathiacalix[4]arene in *cone* conformation [**G2.5-cone**]. Viscous yellowish oil, yield: 0.60 g (90%).

^{1}H NMR (CD_3OD, δ, ppm, *J*/Hz): 1.15 (br.s, 36H, $(CH_3)_3C$), 1.28–1.41 (m, 16H, $CH_2CH_2C\underline{H_2}C\underline{H_2}CH_2CH_2$), 1.44–1.54 (m, 8H, $NCH_2C\underline{H_2}CH_2$), 1.55–1.66 (m, 8H, $NHCH_2C\underline{H_2}CH_2$), 2.38 (br.t, 48H, $NCH_2C\underline{H_2}C(O)NH$), 2.42–2.50 (m, 72H, $NC\underline{H_2}CH_2CH_2$,

$NCH_2C\underline{H}_2C(O)OCH_3$), 2.51–2.65 (m, 48H, $NHCH_2C\underline{H}_2N$), 2.71–2.87 (m, 112H, $NC\underline{H}_2CH_2C(O)$), 3.22–3.34 (m, 56H, $NHC\underline{H}_2$), 3.66 (s, 96H, OCH_3), 4.90 (br.s, 8H, $OCH_2C(O)$), 7.45 (br.s, 8H, Ar$\underline{H}$).t

$^{13}C\{^1H\}$ NMR (CD_3OD, δ, ppm): 27.85, 28.18, 28.47, 30.76, 31.70, 33.59, 34.31, 34.75, 35.24, 38.49, 38.61, 40.38, 50.50, 50.84, 51.07, 52.21, 53.50, 53.80, 54.49, 75.41, 129.80, 136.11, 148.81, 159.33, 170.40, 174.59, 174.67.

FTIR ATR (ν, cm^{-1}): 3288 (N-H), 3074 (N-H), 1732 (C=O), 1642 (C(O)NH, amide I), 1539 (C(O)NH, amide II), 1095 ($C_{Ph}OCH_2$).

5,11,17,23-Tetra-*tert*-butyl-25,26,27,28-tetrakis[*N*-(6-(*N*,*N*-di(*N*-(2-(*N*,*N*-di(*N*-(2-(*N*,*N*-di(methoxycarbonylethyl)amino)ethyl)carbamoylethyl)amino)ethyl)carbamoylethyl)amino)hexyl)carbamoylmethoxy]-2,8,14,20-tetrathiacalix[4]arene in *partial cone* conformation [**G2.5-paco**]. Viscous yellowish oil, yield: 0.63 g (94%).

^1H NMR (CD_3OD, δ, ppm, *J*/Hz): 1.09 (s, 18H, $(CH_3)_3C$), 1.35 (s, 18H, $(CH_3)_3C$), 1.37–1.72 (m, 32H, $CH_2C\underline{H}_2C\underline{H}_2C\underline{H}_2C\underline{H}_2CH_2$), 2.38 (br.t, 48H, $NCH_2C\underline{H}_2CO$), 2.42–2.51 (m, 72H, $NCH_2C\underline{H}_2CH_2$, $NCH_2C\underline{H}_2CO$), 2.52–2.63 (m, 48H, $NHCH_2C\underline{H}_2N$, $NHCH_2C\underline{H}_2N$), 2.72–2.86 (m, 112H, $NC\underline{H}_2CH_2CO$), 3.16-3.34 (m, 56H, $NHC\underline{H}_2$), 3.66 (s, 96H, $OC\underline{H}_3$), 4.28 (br.d, 2H, $OCH_2C(O)$), 4.60 (br.s, 2H, $OCH_2C(O)$), 4.86 (br.s, 2H, $OCH_2C(O)$), 5.00 (br.d, 2H, $OC\underline{H}_2CONH$), 7.12 (br.d, 2H, Ar$\underline{H}$), 7.62 (br.d, 2H, Ar$\underline{H}$), 7.66 (s, 2H, Ar$\underline{H}$), 7.84 (s, 2H, Ar$\underline{H}$).

$^{13}C\{^1H\}$ NMR (CD_3OD, δ, ppm): 27.91, 28.18, 28.36, 28.46, 30.63, 30.76, 31.76, 31.86, 31.95, 33.60, 34.35, 34.77, 34.95, 35.21, 35.33, 38.50, 38.63, 40.04, 40.15, 40.42, 50.51, 50.85, 51.08, 52.21, 53.51, 53.81, 54.42, 54.49, 54.64, 70.86, 74.18, 74.58, 127.99, 128.07, 129.50, 129.97, 134.75, 135.59, 135.96, 137.53, 147.18, 147.62, 148.81, 157.93, 159.41, 160.89, 170.03, 170.36, 170.71, 174.62, 174.69.

FTIR ATR (ν, cm^{-1}): 3289 (N-H), 3074 (N-H), 1732 (C=O), 1642 (C(O)NH, amide I), 1538 (C(O)NH, amide II), 1090 ($C_{Ph}OCH_2$).

5,11,17,23-Tetra-*tert*-butyl-25,26,27,28-tetrakis[*N*-(6-(*N*,*N*-di(*N*-(2-(*N*,*N*-di(*N*-(2-(*N*,*N*-di(methoxycarbonylethyl)amino)ethyl)carbamoylethyl)amino)ethyl)carbamoylethyl)amino)hexyl)carbamoylmethoxy]-2,8,14,20-tetrathiacalix[4]arene in *1,3-alternate* conformation [**G2.5-alt**]. Viscous yellowish oil, yield: 0.61 g (91%).

^1H NMR (CD_3OD, δ, ppm, *J*/Hz): 1.28 (s, 36H, $(CH_3)_3C$), 1.31–1.39 (m, 16H, $CH_2CH_2C\underline{H}_2C\underline{H}_2CH_2CH_2$), 1.45–1.62 (m, 16H, $CH_2C\underline{H}_2CH_2N$, $C(O)NHCH_2C\underline{H}_2CH_2$), 2.38 (br.t, 48H, $NCH_2C\underline{H}_2C(O)NH$), 2.46 (m, 72H, $NC\underline{H}_2CH_2CH_2$, $NCH_2C\underline{H}_2C(O)OCH_3$), 2.50–2.66 (m, 48H, $NHCH_2C\underline{H}_2N$), 2.72–2.86 (m, 112H, $NC\underline{H}_2CH_2C(O)$), 3.17–3.30 (m, 56H, $NHC\underline{H}_2$), 3.66 (s, 96H, OCH_3), 4.11 (br.s, 8H, $OCH_2C(O)$), 7.61 (br.s, 8H, Ar$\underline{H}$).

$^{13}C\{^1H\}$ NMR (CD_3OD, δ, ppm): 27.85, 28.22, 28.39, 30.79, 31.78, 33.59, 34.31, 34.74, 35.35, 38.49, 38.62, 40.70, 50.50, 50.83, 51.06, 52.21, 53.49, 53.80, 54.46, 71.69, 129.19, 133.49, 148.83, 157.94, 169.82, 174.60, 174.68.

FTIR ATR (ν, cm^{-1}): 3299 (N-H), 3074 (N-H), 1731 (C=O), 1642 (C(O)NH, amide I), 1536 (C(O)NH, amide II), 1086 ($C_{Ph}OCH_2$).

1.3. General procedure for the synthesis of G3 PAMAM-calix-dendrimers.

The solution (10% by the weight) of **G2.5** (0.60 g, 0.088 mmol) in methanol was added dropwise to an ice-cooled (0 °C) solution of ethylenediamine (3.75 mL, 56 mmol) in 4 mL of methanol. It was kept in the ice bath for 2 h. Then, the reaction mixture was stirred at room temperature (25 °C) for 110 h. Afterward, the solvent was removed on a rotary evaporator, and the excess of ethylenediamine was removed by azeotropic distillation with the mixture methanol:toluene (1:9). Then, the remaining toluene was removed by azeotropic distillation with methanol. The residue was dried under reduced pressure.

5,11,17,23-Tetra-*tert*-butyl-25,26,27,28-tetrakis[*N*-(6-(*N*,*N*-di(*N*-(2-(*N*,*N*-di(*N*-(2-(*N*,*N*-di(*N*-(2-aminoethyl)carbamoylethyl)amino)ethyl)carbamoylethyl)amino)ethyl)carbamoylethyl) amino)hexyl)carbamoylmethoxy]-2,8,14,20-tetrathiacalix[4]arene in *cone* conformation [**G3-cone**]. White solid foam, mp 68 °C, yield: 0.54 g (80%)

^{1}H NMR (CD_3OD, δ, ppm, *J*/Hz): 1.14 (br.s, 36H, $(CH_3)_3C$), 1.28–1.41 (m, 16H, $CH_2CH_2C\underline{H_2}C\underline{H_2}CH_2CH_2$), 1.42–1.53 (m, 8H, $NCH_2C\underline{H_2}CH_2$), 1.54–1.67 (m, 8H, $NHCH_2C\underline{H_2}CH_2$), 2.30–2.42 (m, 112H, $NCH_2C\underline{H_2}CO$), 2.43–2.53 (m, 8H, $NCH_2C\underline{H_2}CH_2$), 2.54–2.62 (m, 48H, $NHCH_2C\underline{H_2}N$), 2.68–2.85 (m, 176H, $NHCH_2C\underline{H_2}NH_2$, $NC\underline{H_2}CH_2CO$), 3.20–3.32 (m, 120H, $NHC\underline{H_2}$), 4.91 (br.s, 8H, $OCH_2C(O)$), 7.44 (br.s, 8H, Ar$\underline{H}$).

$^{13}C\{^1H\}$ NMR (CD_3OD, δ, ppm): 27.87, 28.15, 28.45, 30.71, 31.68, 34.42, 34.79, 38.60, 40.35, 42.05, 43.02, 50.85, 51.13, 53.49, 54.48, 54.62, 75.41, 129.77, 136.06, 148.80, 159.26, 170.41, 174.64, 175.07.

FTIR ATR (ν, cm^{-1}): 3278 (N-H), 3065 (N-H), 1636 (C(O)NH, amide I), 1540 (C(O)NH, amide II), 1245 (C(O)NH, amide III), 1095 ($C_{Ph}OCH_2$).

ESI HRMS, Calculated $[M + 16H + Na]^{17+}$ *m*/*z* = 457.3709. Found $[M + 16H + Na]^{17+}$ *m*/*z* = 457.3234.

5,11,17,23-Tetra-*tert*-butyl-25,26,27,28-tetrakis[*N*-(6-(*N*,*N*-di(*N*-(2-(*N*,*N*-di(*N*-(2-(*N*,*N*-di(*N*-(2-aminoethyl)carbamoylethyl)amino)ethyl)carbamoylethyl)amino)ethyl)carbamoylethyl) amino)hexyl)carbamoylmethoxy]-2,8,14,20-tetrathiacalix[4]arene in *partial cone* conformation [**G3-paco**]. White solid foam, mp 70 °C, Yield: 0.59 g (87%)

^{1}H NMR (CD_3OD, δ, ppm, *J*/Hz): 1.09 (s, 16H, $(C\underline{H_3})_3C$), 1.35 (s, 16H, $(C\underline{H_3})_3C$), 1.38–1.73 (m, 32H, $CH_2C\underline{H_2}C\underline{H_2}C\underline{H_2}C\underline{H_2}CH_2$), 2.29–2.42 (m, 112H, $NCH_2C\underline{H_2}CO$), 2.44–2.53 (m, 8H, $NCH_2C\underline{H_2}CH_2$), 2.54–2.65 (m, 48H, $NHCH_2C\underline{H_2}N$), 2.68–2.87 (m, 176H, $NHCH_2C\underline{H_2}NH_2$, $NC\underline{H_2}CH_2CO$), 3.28–3.36 (m, 120H, $NHC\underline{H_2}$), 4.21–4.36 (m, 2H, $OCH_2C(O)$), 4.60 (br.s, 2H, $OCH_2C(O)$), 4.86 (br.s, 2H, $OCH_2C(O)$), 4.92–5.10 (m, 2H, $OC\underline{H_2}CONH$), 7.12 (br.d, 2H, Ar$\underline{H}$), 7.62 (br.d, 2H, Ar$\underline{H}$), 7.66 (s, 2H, Ar$\underline{H}$), 7.84 (s, 2H, Ar$\underline{H}$).

$^{13}C\{^1H\}$ NMR (CD_3OD, δ, ppm): 27.90, 28.17, 28.38, 28.46, 30.60, 30.76, 31.76, 31.85, 31.93, 34.41, 34.80, 38.63, 40.08, 42.05, 43.01, 50.84, 51.14, 53.51, 54.44, 54.65, 70.84, 74.16, 74.57, 128.06, 128.18, 129.51, 129.89, 134.82, 135.60, 135.99, 137.45, 147.05, 147.58, 148.79, 157.88, 159.36, 160.91, 170.04, 170.27, 170.77, 174.69, 175.11.

FTIR ATR (ν, cm^{-1}): 3278 (N-H), 3065 (N-H), 1635 (C(O)NH, amide I), 1539 (C(O)NH, amide II), 1242 (C(O)NH, amide III), 1090 ($C_{Ph}OCH_2$).

ESI HRMS, Calculated $[M + 16H + Na]^{17+}$ *m*/*z* = 457.3709. Found $[M + 16H + Na]^{17+}$ *m*/*z* = 457.3238.

5,11,17,23-Tetra-*tert*-butyl-25,26,27,28-tetrakis[*N*-(6-(*N*,*N*-di(*N*-(2-(*N*,*N*-di(*N*-(2-(*N*,*N*-di(*N*-(2-aminoethyl)carbamoylethyl)amino)ethyl)carbamoylethyl)amino)ethyl)carbamoylethyl) amino)hexyl)carbamoylmethoxy]-2,8,14,20-tetrathiacalix[4]arene in *1,3-alternate* conformation [**G3-alt**]. White solid foam, mp 70 °C, Yield: 0.57 g (84%).

^{1}H NMR (CD_3OD, δ, ppm, *J*/Hz): 1.28 (s, 36H, $(CH_3)_3C$), 1.29–1.38 (m, 16H, $C(O)NHCH_2CH_2C\underline{H}_2C\underline{H}_2$), 1.44–1.64 (m, 16H, $CH_2C\underline{H}_2CH_2N$, $C(O)NHCH_2C\underline{H}_2CH_2$), 2.32–2.42 (m, 112H, $NCH_2C\underline{H}_2C(O)$), 2.44–2.52 (m, 8H, $CH_2CH_2C\underline{H}_2N$), 2.53–2.63 (m, 48H, $NHCH_2C\underline{H}_2N$), 2.69–2.86 (m, 176H, $NHCH_2C\underline{H}_2NH_2$, $NC\underline{H}_2CH_2C(O)$), 3.17–3.30 (m, 120H, $NHC\underline{H}_2$), 4.00–4.21 (m, 8H, $OCH_2C(O)$), 7.60 (s, 8H, Ar$\underline{H}$).

$^{13}C\{^{1}H\}$ NMR (CD_3OD, δ, ppm): 27.87, 28.23, 28.40, 30.78, 31.79, 34.40, 34.79, 38.62, 40.73, 42.04, 42.98, 50.84, 51.14, 53.50, 54.47, 54.65, 71.69, 129.22, 133.65, 148.80, 157.92, 169.86, 174.70, 175.13.

FTIR ATR (ν, cm^{-1}): 3278 (N-H), 3065 (N-H), 1636 (C(O)NH, amide I), 1539 (C(O)NH, amide II), 1244 (C(O)NH, amide III), 1087 ($C_{Ph}OCH_2$).

ESI HRMS, Calculated $[M + 16H + Na]^{17+}$ *m*/*z* = 457.3709. Found $[M + 16H + Na]^{17+}$ *m*/*z* = 457.3261.

1.4 Preparation of PAMAM-calix-dendrimer samples

Ammonium salt forms of **PAMAM-calix-dendrimers** were used for the study of the interactions of the dendrimers with DNA. The solutions were prepared by adding calculated volume of 0.25 M (for the first and second generations) and 0.5 M (for the third generation) hydrochloric acid to the dendrimers. The volume of hydrochloric acid was chosen to ensure reaction of all primary and tertiary amino groups of the compounds.

1.5. UV-Vis spectroscopy

UV-Vis spectra were recorded on the Shimadzu UV-3600 spectrophotometer (Kyoto, Japan) using the 1 cm quartz cuvette at 293 K in the 200–550 nm wavelength range in 10 mM Tris-HCl buffer (pH = 7.4). Calf thymus DNA was used as received. The purity of the DNA was checked by the ratio of the absorbance A260/A280 > 1.8, indicating the DNA was sufficiently free from protein. The concentration of DNA was 20 μM in nucleotide base pairs. The concentrations of **PAMAM-calix-dendrimers** were 10 μM. UV-Vis spectra were recorded 30 min after preparation.

1.6. Fluorescence spectroscopy

Fluorescence spectra were recorded on the Fluorolog 3 luminescent spectrometer (Horiba Jobin Yvon, Longjumeau, France). The excitation wavelength was selected as 525 nm. The emission scan range was 550–700 nm. Excitation and emission slits were 5 nm. Quartz cuvettes with an optical path length of 1 mm were used. Fluorescence spectra were automatically corrected by the Fluorescence program. The spectra were recorded at

293 K in 10 mM Tris-HCl buffer (pH = 7.4). The concentration of ethidium bromide (EB) was 5 μM. The concentration of DNA was 18 μM in nucleotide base pairs. The concentrations of **PAMAM-calix-dendrimers** ranged from 0.5 to 60 μM. First, the DNA/EB systems were prepared. The mixtures were incubated for 30 min, after which **PAMAM-calix-dendrimers** were added. The fluorescence spectra were recorded 1 h after preparation. The values of **PAMAM-calix-dendrimers** concentrations (C_{50}, μM) were determined at a 50% decrease in the emission intensity (F_{50}) of the DNA/EB system from the initial one was observed (F_{50}=(F_0–F_{min})/2).

1.7. Circular dichroism (CD)

Changes in the intensity of the CD signal were obtained using the Jasco J-1500 spectropolarimeter (Tokyo, Japan) in the quartz cuvette with the 10 mm optical path length. The CD spectra of DNA (20 μM in nucleotide base pairs) with/without **PAMAM-calix-dendrimers** (10 μM) were recorded at 293 K in the 223–325 nm wavelength range. The CD spectra of the DNA/EB (20 μM in nucleotide base pairs / 10 μM) systems with/without **PAMAM-calix-dendrimers** (10 μM) were recorded at 293 K in the 223–400 nm wavelength range. The experiment was carried out in 10 mM Tris-HCl buffer (pH = 7.4). The CD spectra were recorded 30 min after addition the dendrimers. The scanning rate was 20 nm/min.

1.8. Dynamic light scattering (DLS)

The size distribution of particles formed as a result of self-association of the dendrimers and their association with DNA was studied by DLS using the Zetasizer Nano ZS (Malvern) in polystyrene cuvettes at 293 K. The solutions of the individual compounds and their mixtures were studied at a constant DNA concentration (10 μM and 20 μM in nucleotide base pairs) and varying concentrations of **PAMAM-calix-dendrimers** (1–500 μM for **G1** and **G2**; 0.5–30 μM for **G3-HCl**).

1.9. Electrokinetic Potentials.

Electrokinetic (ζ) potentials were determined by electrophoretic light scattering on the Zetasizer Nano ZS (Malvern Instruments, Worcestershire, UK). Samples were prepared for the DLS measurements and transferred with the syringe to the disposable folded capillary cell for measurement. The ζ potentials were measured using the Malvern M3-PALS method and averaged from five measurements.

1.10. Transmission electron microscopy (TEM)

TEM measurements were made at Interdisciplinary Center for Analytical Microscopy of Kazan Federal University. Analysis of samples was carried out using a Hitachi HT7700 Exalens atomic transmission electron microscope (Tokyo, Japan). Deionized water was used as a solvent. The aqueous solutions of DNA (0.6 μM in nucleotide base pairs) and **G3** (1 μM) as well as their mixture were studied. The solution (10 μL) was placed on a carbon-coated 3 mm copper grid and dried at room temperature using special holder for microanalysis. After drying, the grid was placed in the transmission electron microscope and analyzed at an accelerating voltage of 100 kV.

1.11. Hemolytic activity of PAMAM-calix-dendrimers

The solutions of **PAMAM-calix-dendrimers** in PBS were added to the erythrocytes (at 2% hematocrit). The samples were incubated at 37 °C for 1, 3, and 24 h, centrifuged (3000 × g, 10 min, 4 °C), and their absorbance was measured at 540 nm using spectrophotometer Jasco V-630 (Jasco, Tokyo, Japan). The percentage of hemolysis was calculated using the following formula:

$$H(\%) = \frac{A_{540}}{A_{water}} \times 100\% \qquad (1)$$

where A_{540} is sample absorption; A_{water} is positive control of red blood cells (RBC) in water (100% release of hemoglobin). Data were presented as percentage of hemolysis, mean ± SD, n = 4.

1.12. Platelets aggregation

Blood from healthy donors (men and women 20–30 years) was taken. Aggregation of platelets was studied in platelet rich plasma. For this, blood with addition of 3.8% of sodium citrate was centrifuged at 360 g for 5 min, and erythrocytes and leukocytes were removed to obtain platelet rich plasma. The concentration of platelets in platelet rich plasma was at 225000–230000 cells per μL. Aggregation of platelets was studied using an automatic aggregometer AP2110 (SOLAR, Belarus). In the assay, 400 μL of platelets rich plasma was added to a thermostated (37 °C) plastic tube at a final platelet concentration of 2.25–2.30×10^4 cells per μL. **PAMAM-calix-dendrimers** (25 and 50 μg/mL) were then added to the platelet rich plasma to induce platelets aggregation. The kinetics of aggregation was observed for 15 min with a measurement step of 1 min. Data are expressed as mean ± SD of 6 independent experiments. Significance was assessed using the one-way analysis of variance (ANOVA) with the post-hoc Newman-Keuls multiple comparisons test.

2. NMR, IR and mass spectra

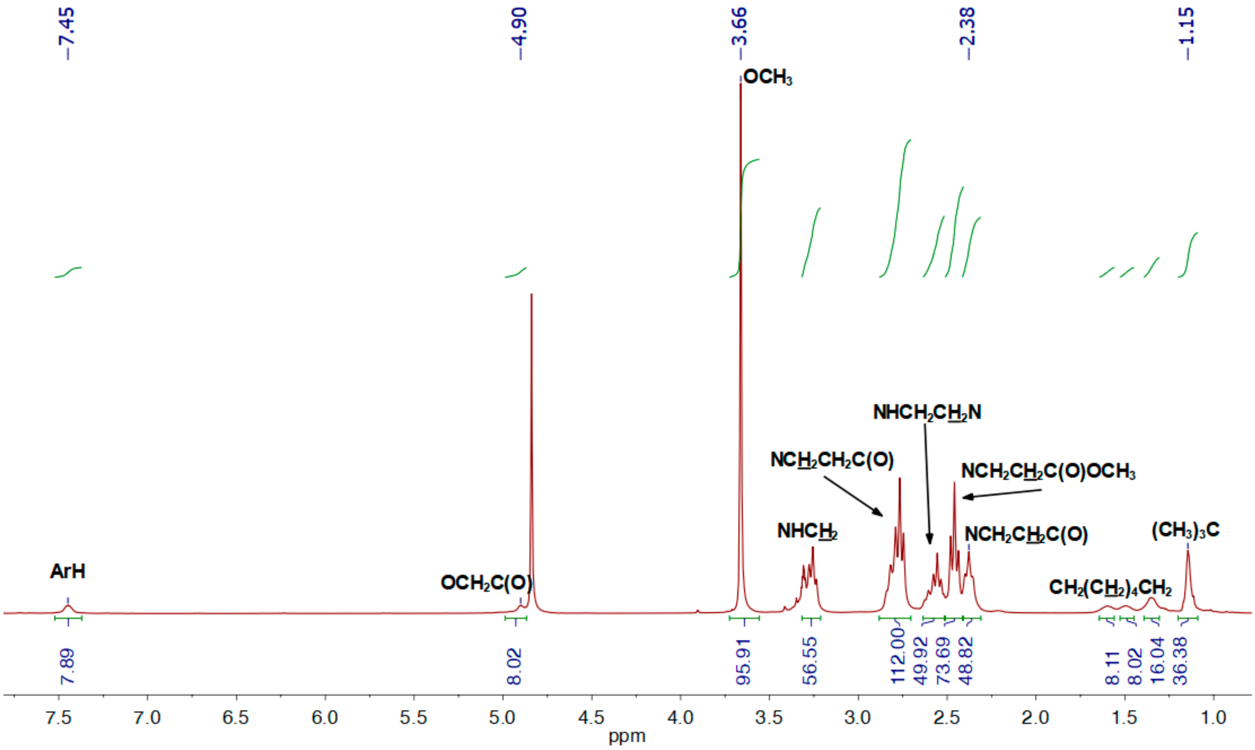

Figure S1. ^{1}H NMR spectrum of **G2.5-cone**, CD_3OD, 298 K, 400 MHz.

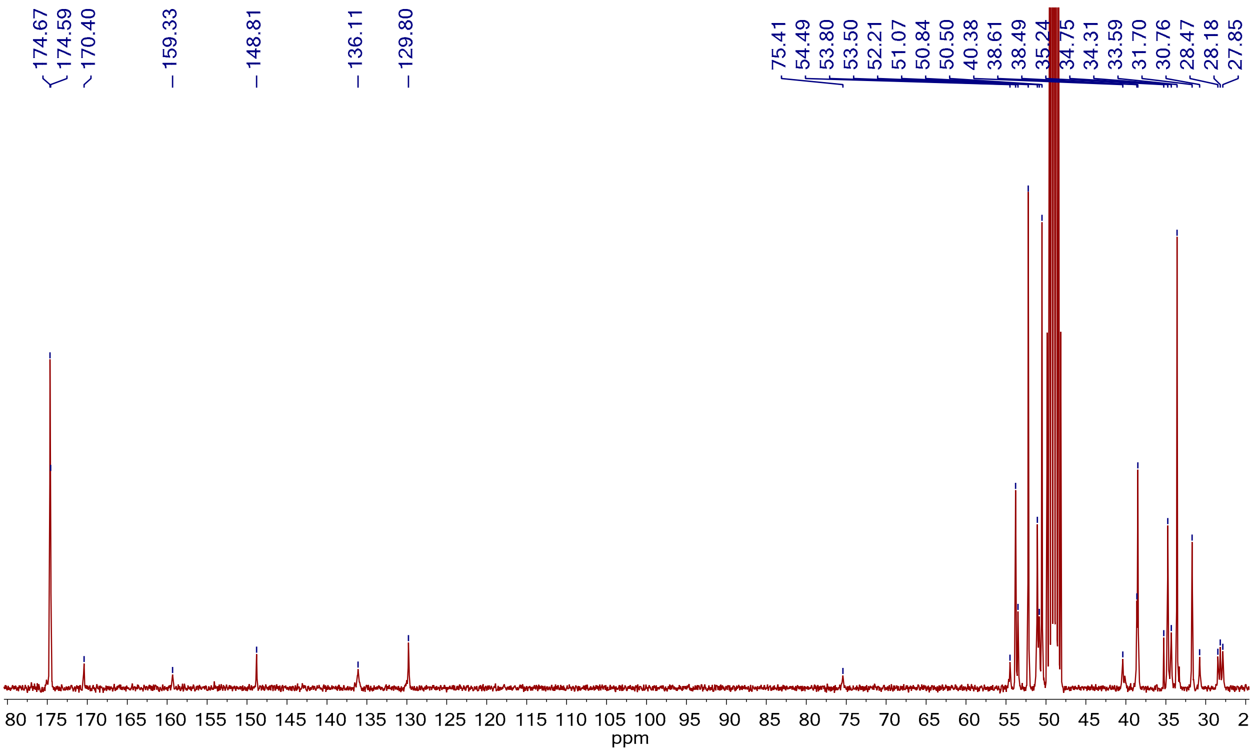

Figure S2. $^{13}C\{^{1}H\}$ NMR spectrum of **G2.5-cone**, CD_3OD, 298 K, 100 MHz.

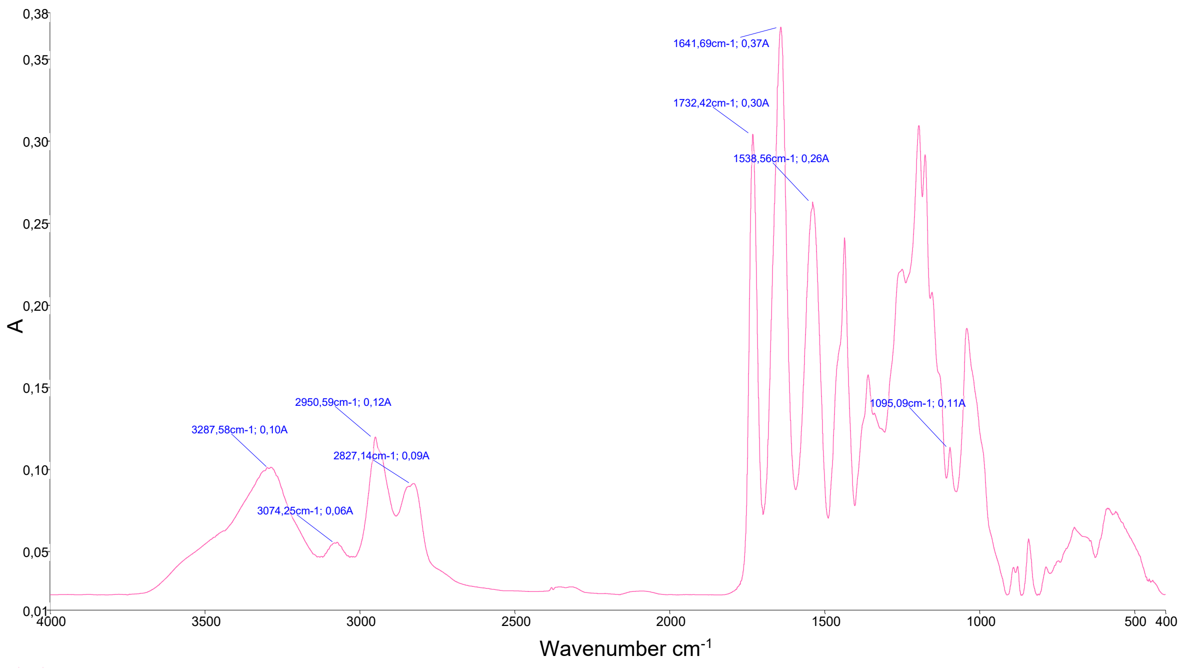

Figure S3. FTIR-ATR spectrum of **G2.5-cone**.

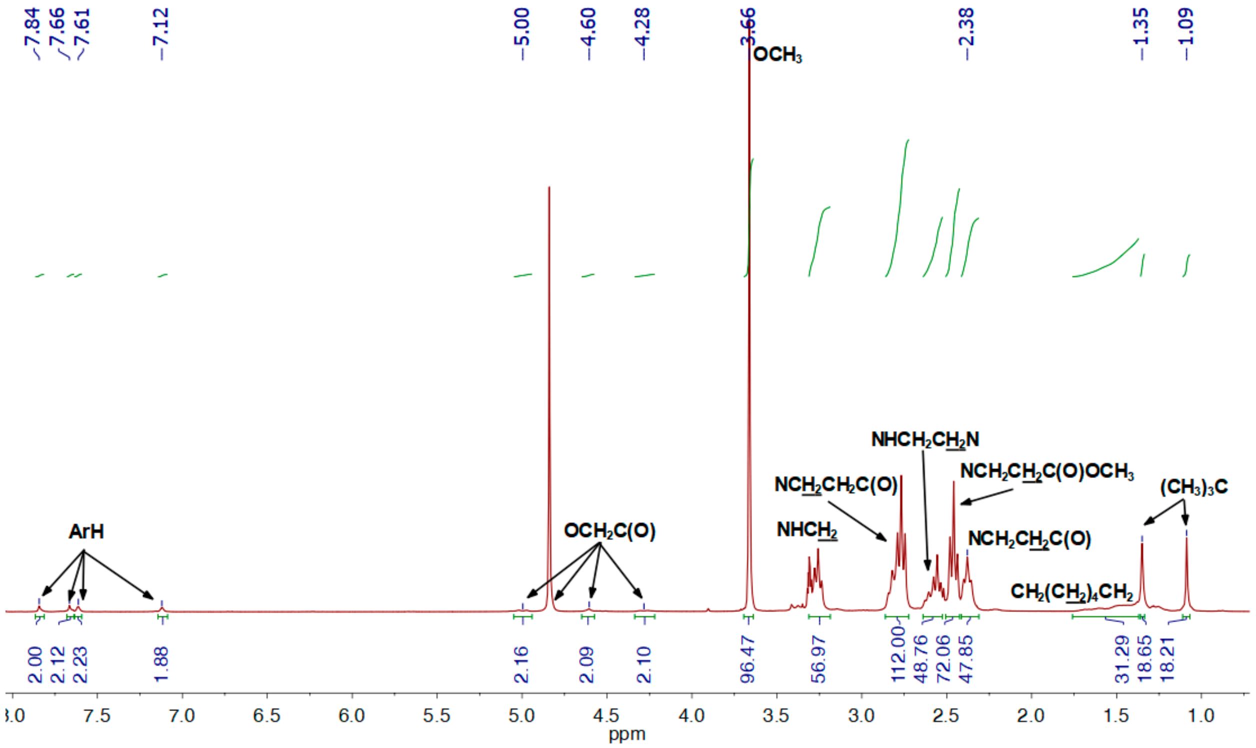

Figure S4. ^{1}H NMR spectrum of **G2.5-paco**, CD_3OD, 298 K, 400 MHz.

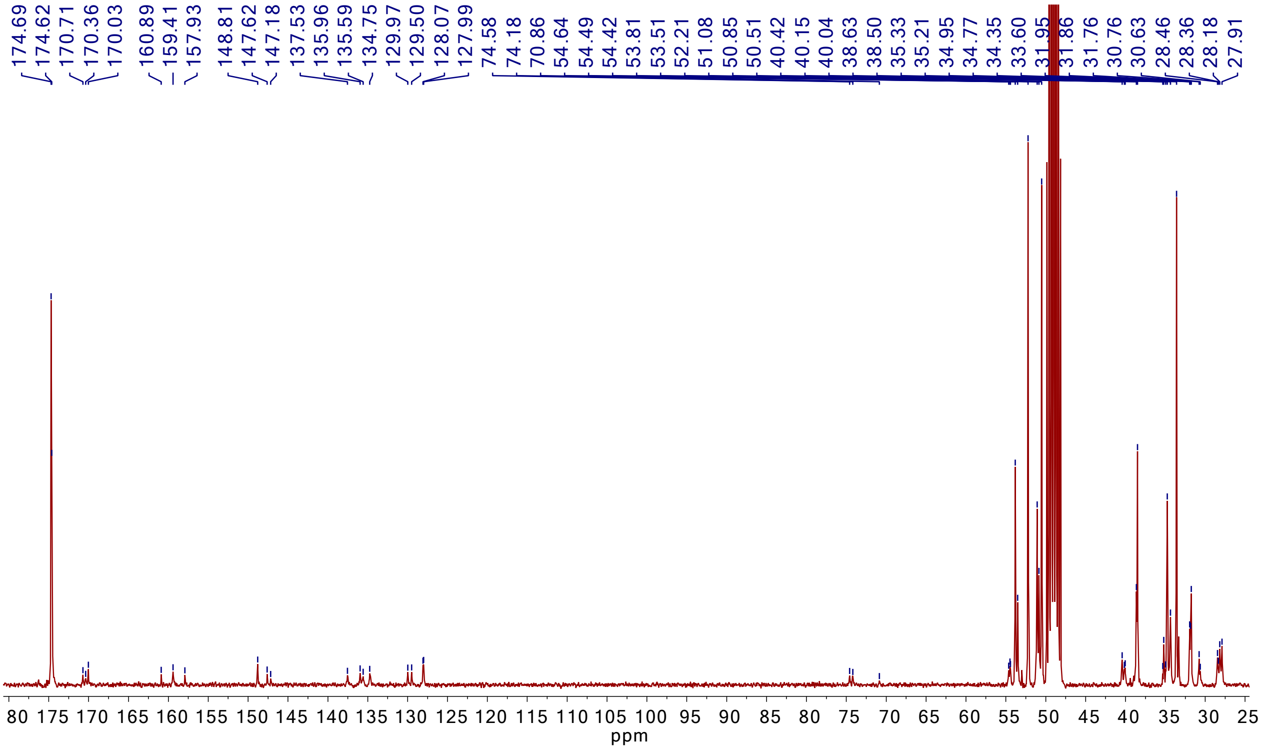

Figure S5. $^{13}C\{^{1}H\}$ NMR spectrum of **G2.5-paco**, CD_3OD, 298 K, 100 MHz.

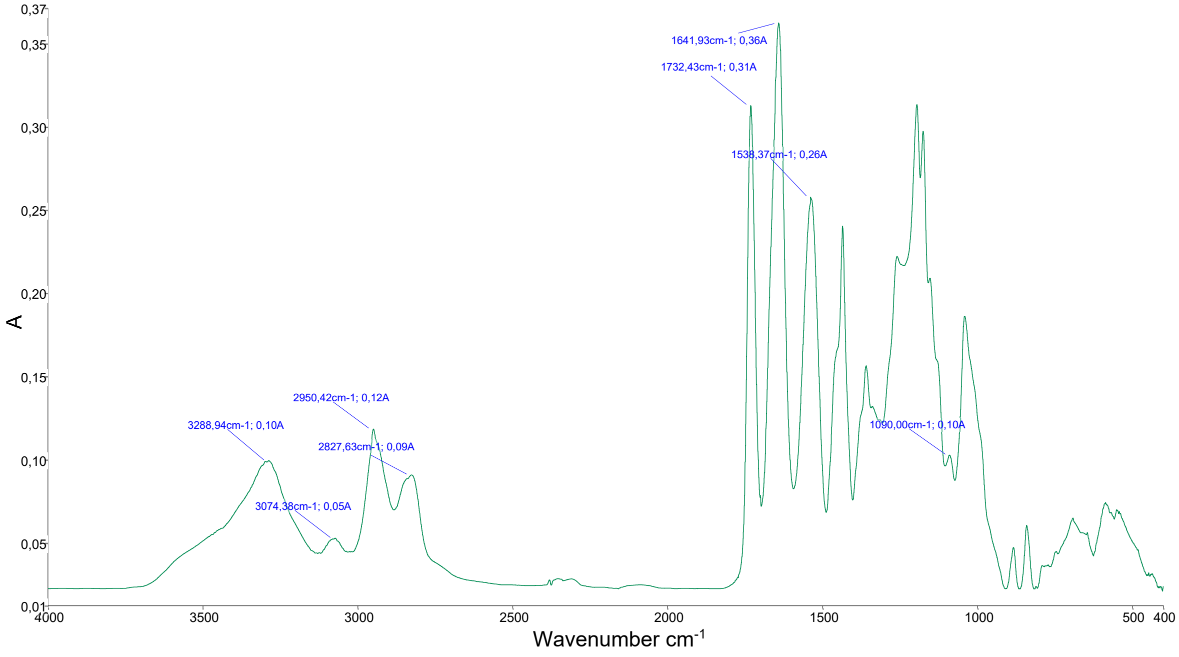

Figure S6. FTIR-ATR spectrum of **G2.5-paco**.

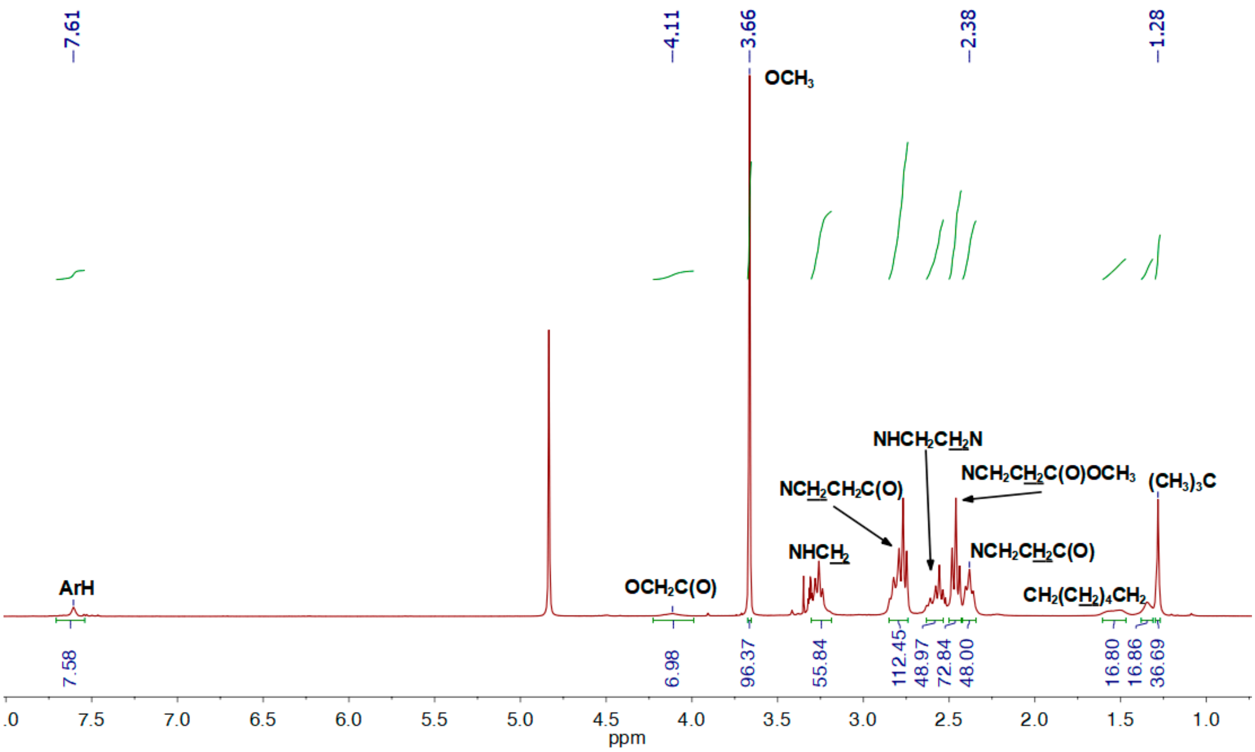

Figure S7. ^{1}H NMR spectrum of **G2.5-alt**, CD_3OD, 298 K, 400 MHz.

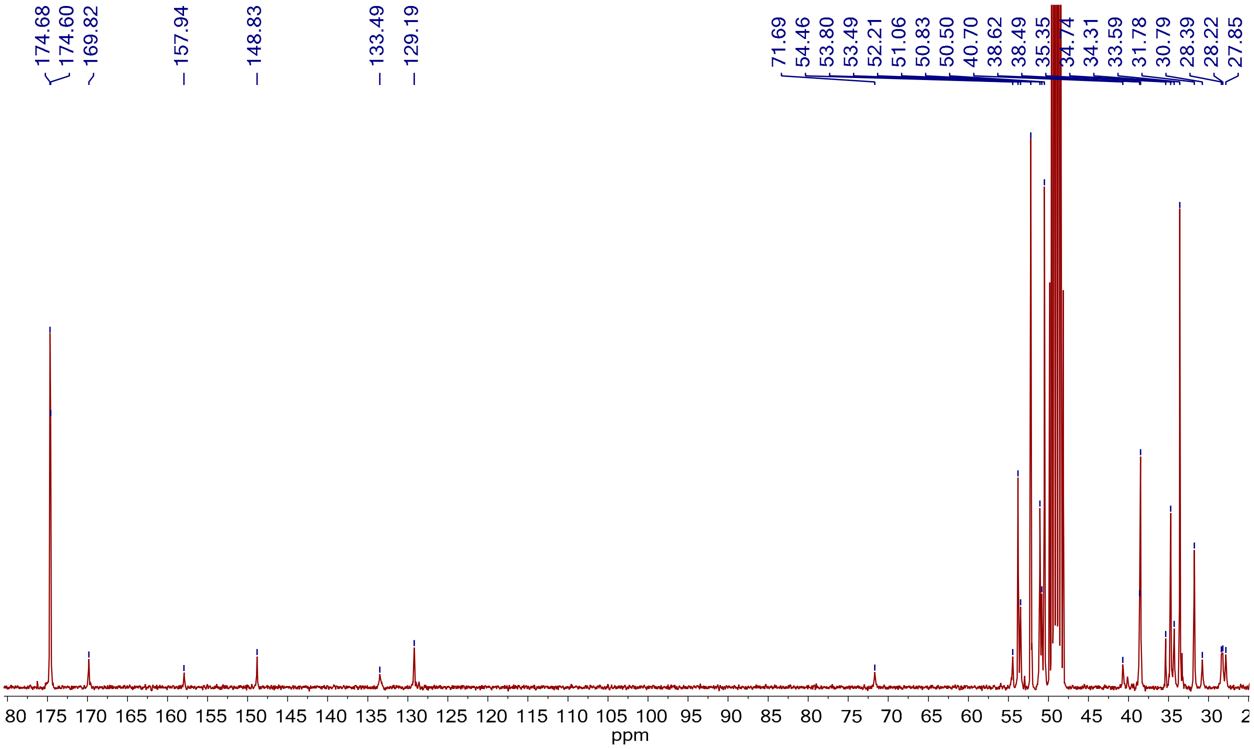

Figure S8. $^{13}C\{^1H\}$ NMR spectrum of **G2.5-alt**, CD_3OD, 298 K, 100 MHz.

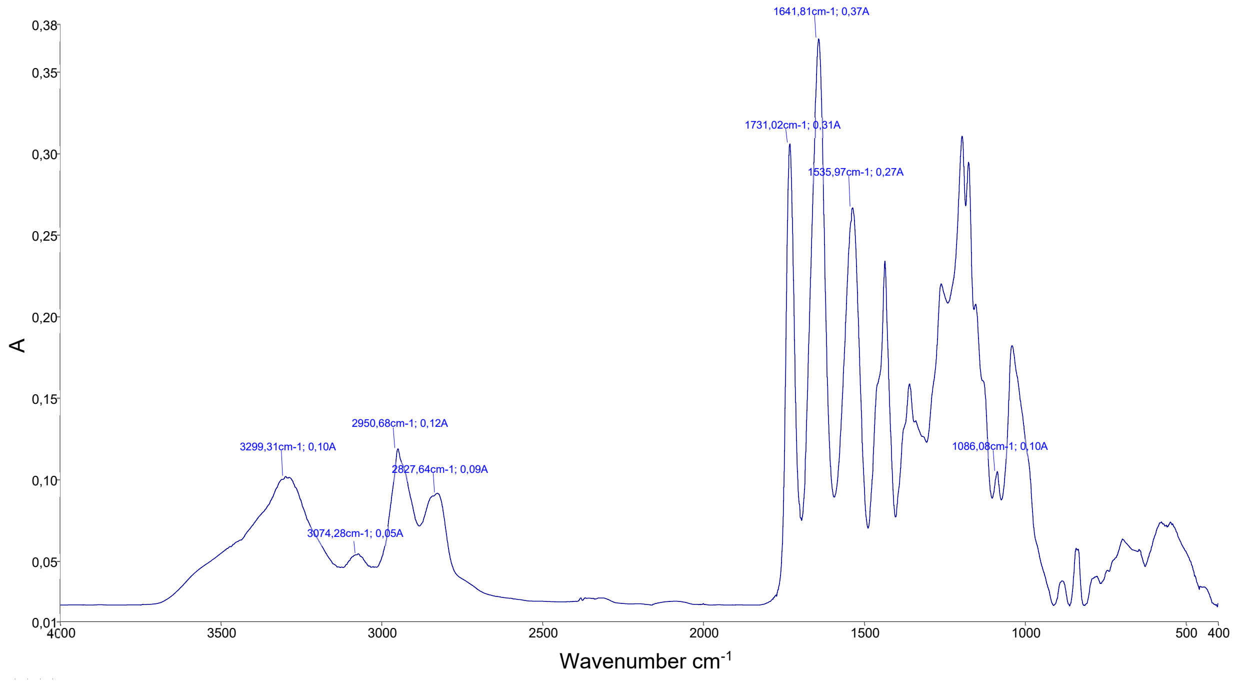

Figure S9. FTIR-ATR spectrum of **G2.5-alt**.

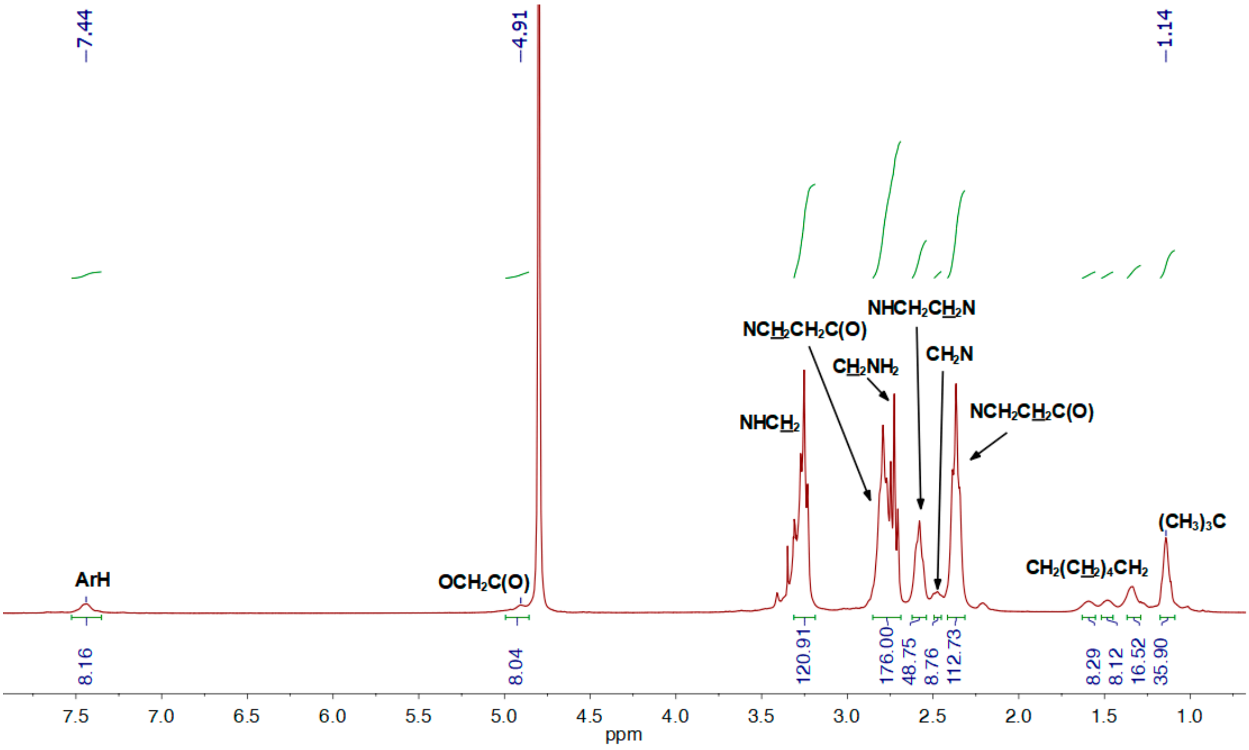

Figure S10. ^{1}H NMR spectrum of **G3-cone**, CD_3OD, 298 K, 400 MHz.

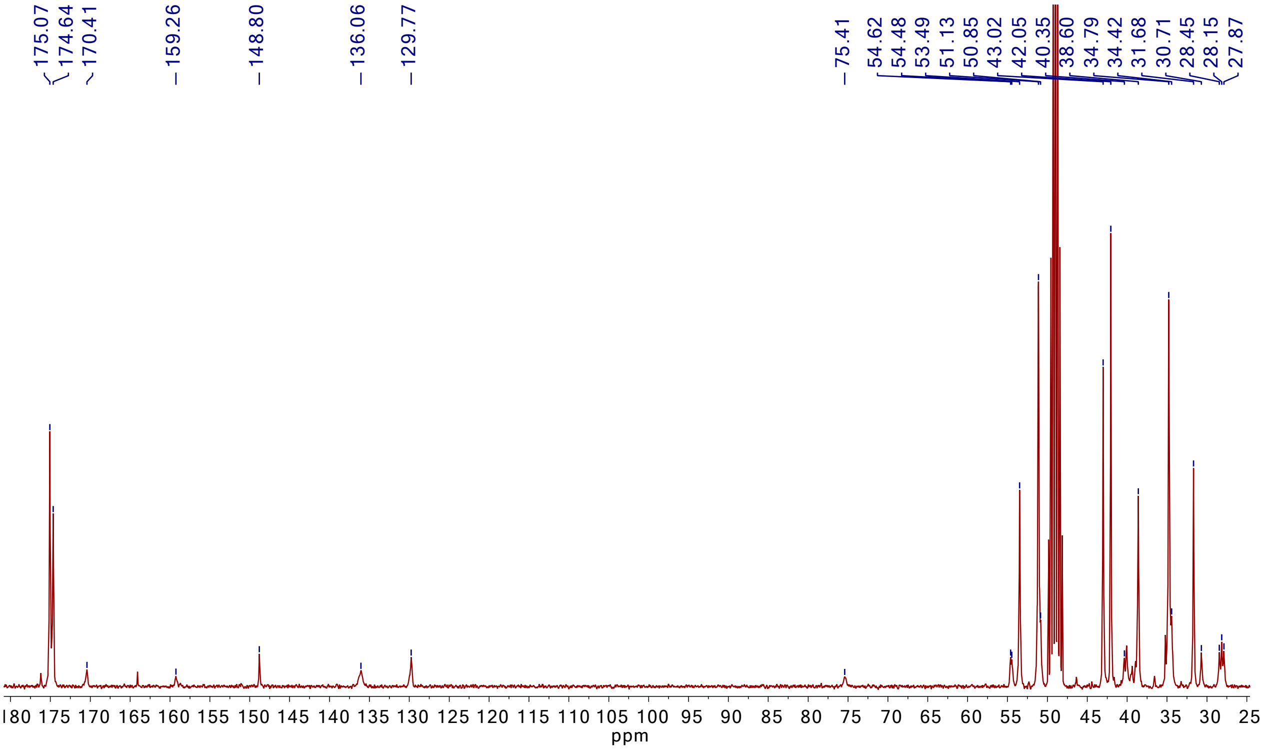

Figure S11. $^{13}C\{^{1}H\}$ NMR spectrum of **G3-cone**, CD_3OD, 298 K, 100 MHz.

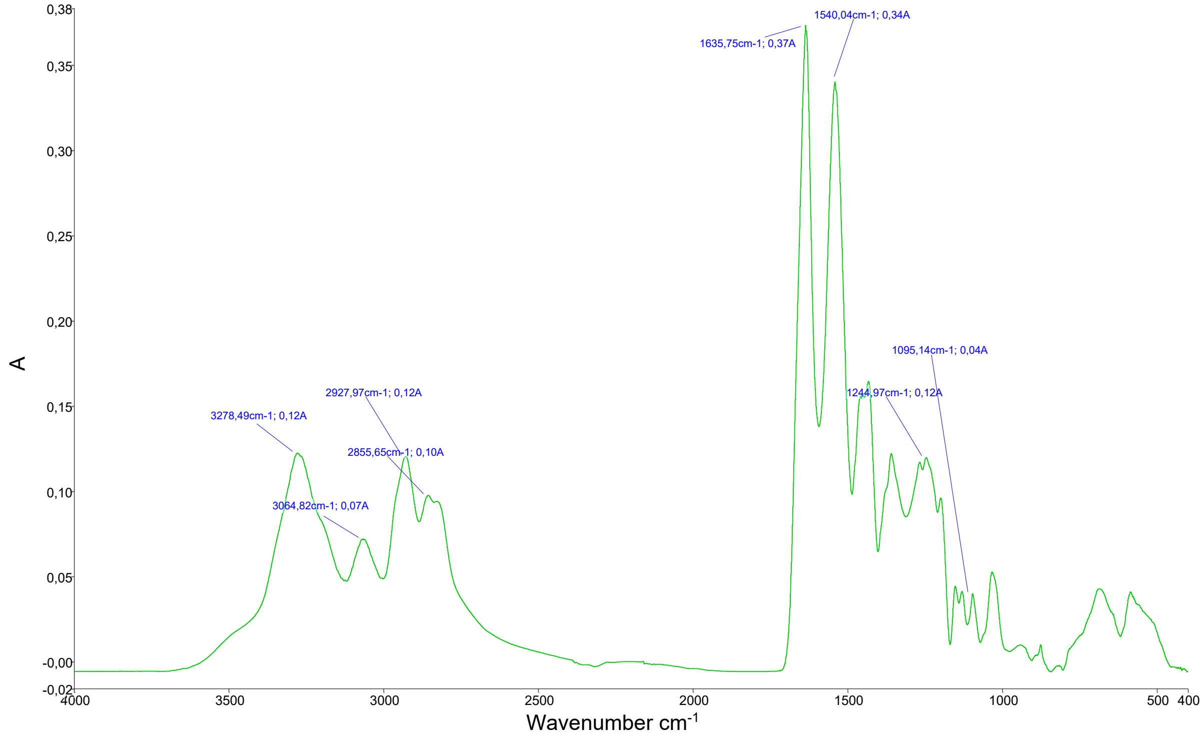

Figure S12. FTIR-ATR spectrum of **G3-cone**.

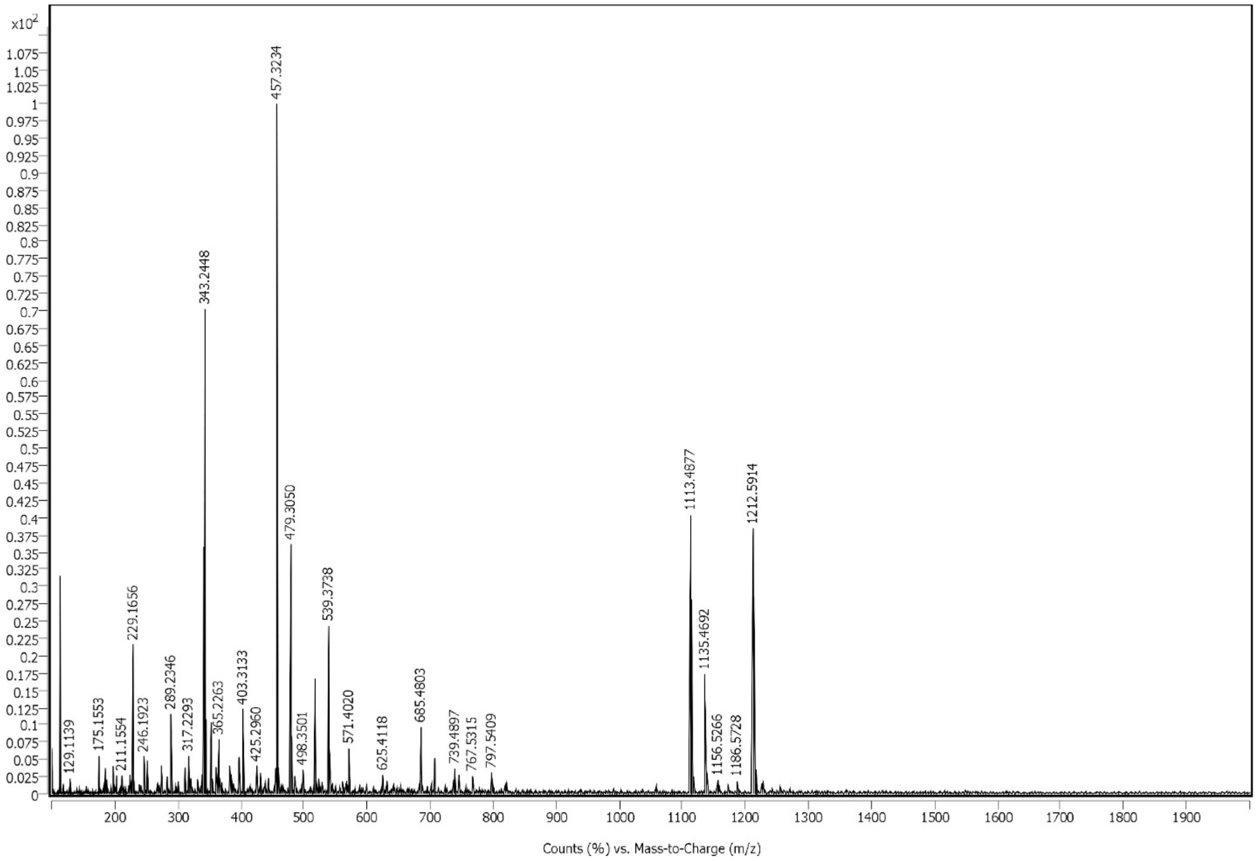

Figure S13. Mass spectrum (HR ESI) of **G3-cone**.

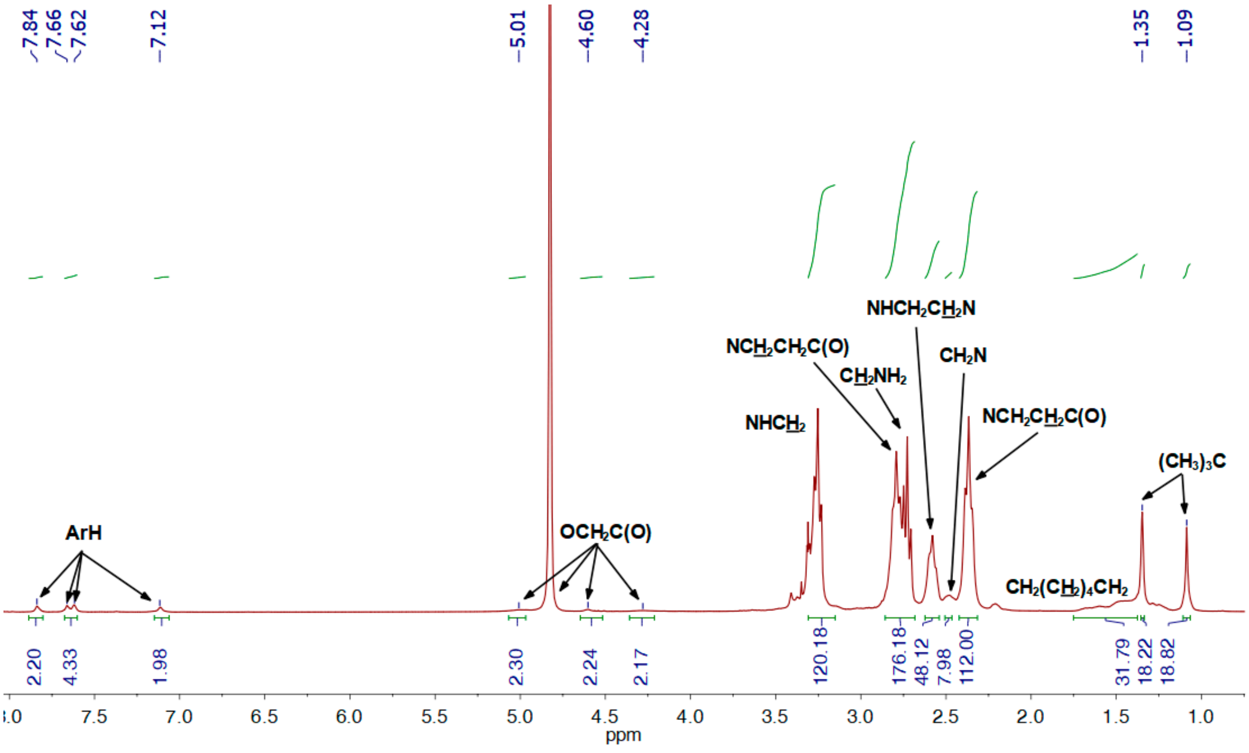

Figure S14. ¹H NMR spectrum of **G3-paco**, CD_3OD, 298 K, 400 MHz.

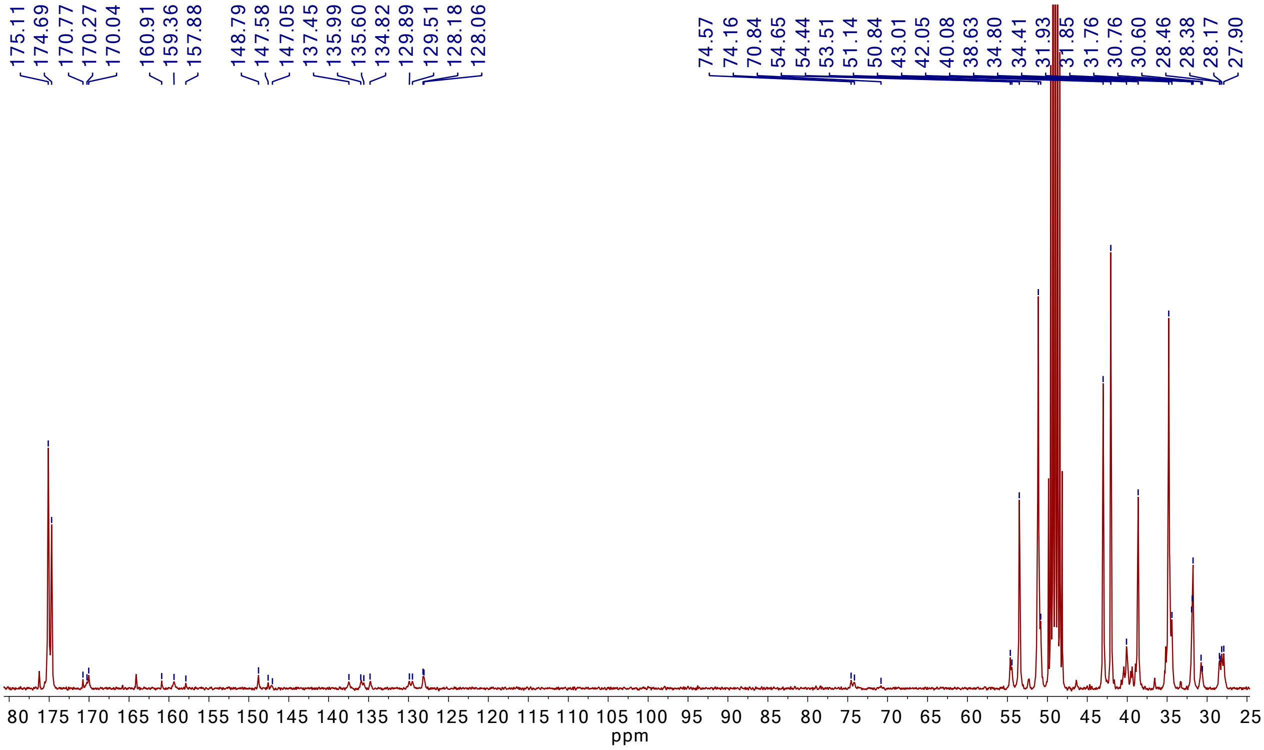

Figure S15. $^{13}C\{^{1}H\}$ NMR spectrum of **G3-paco**, CD_3OD, 298 K, 100 MHz.

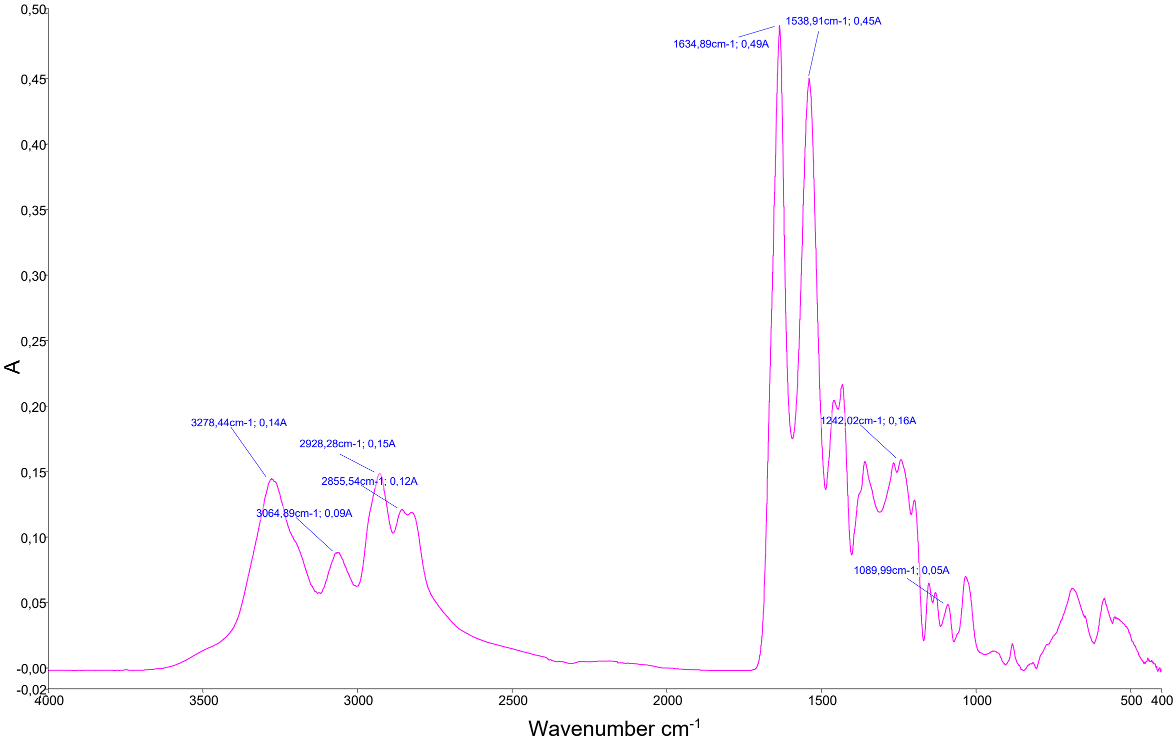

Figure S16. FTIR-ATR spectrum of **G3-paco**.

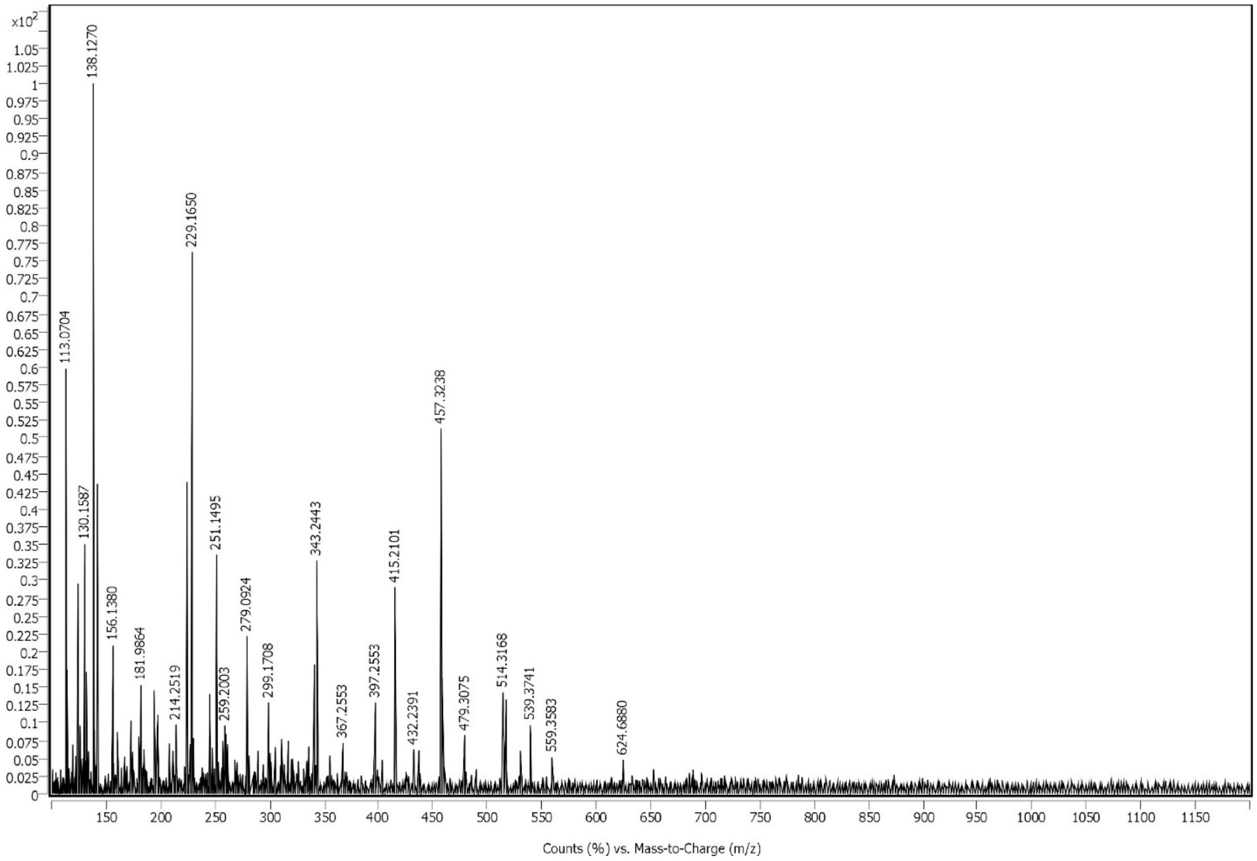

Figure S17. Mass spectrum (HR ESI) of **G3-paco**.

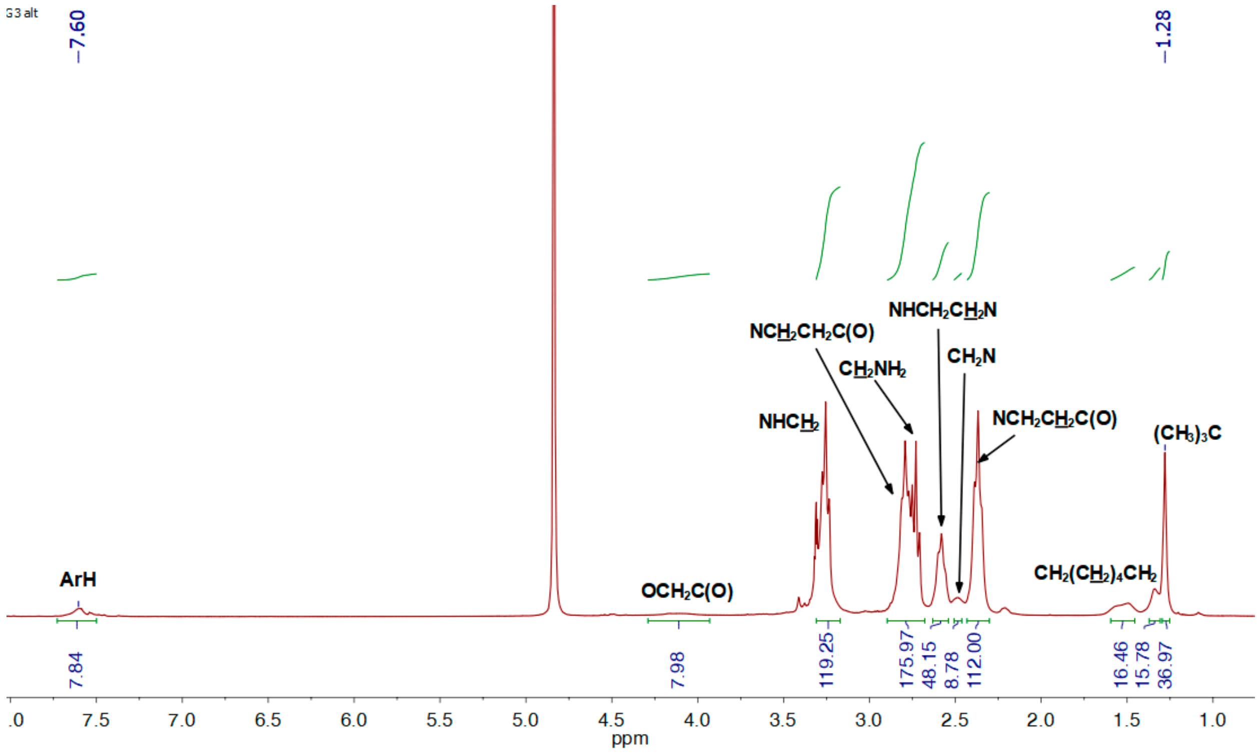

Figure S18. ^{1}H NMR spectrum of **G3-alt**, CD_3OD, 298 K, 400 MHz.

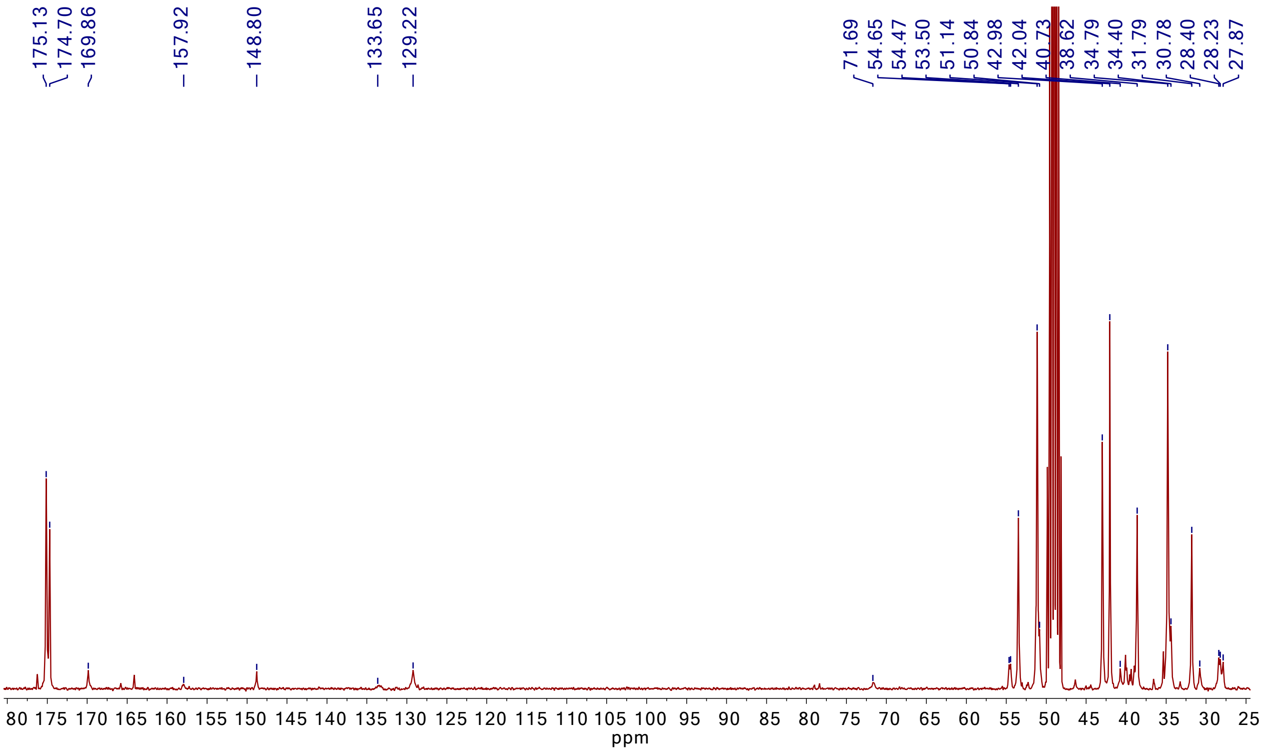

Figure S19. $^{13}C\{^{1}H\}$ NMR spectrum of **G3-alt**, CD_3OD, 298 K, 100 MHz.

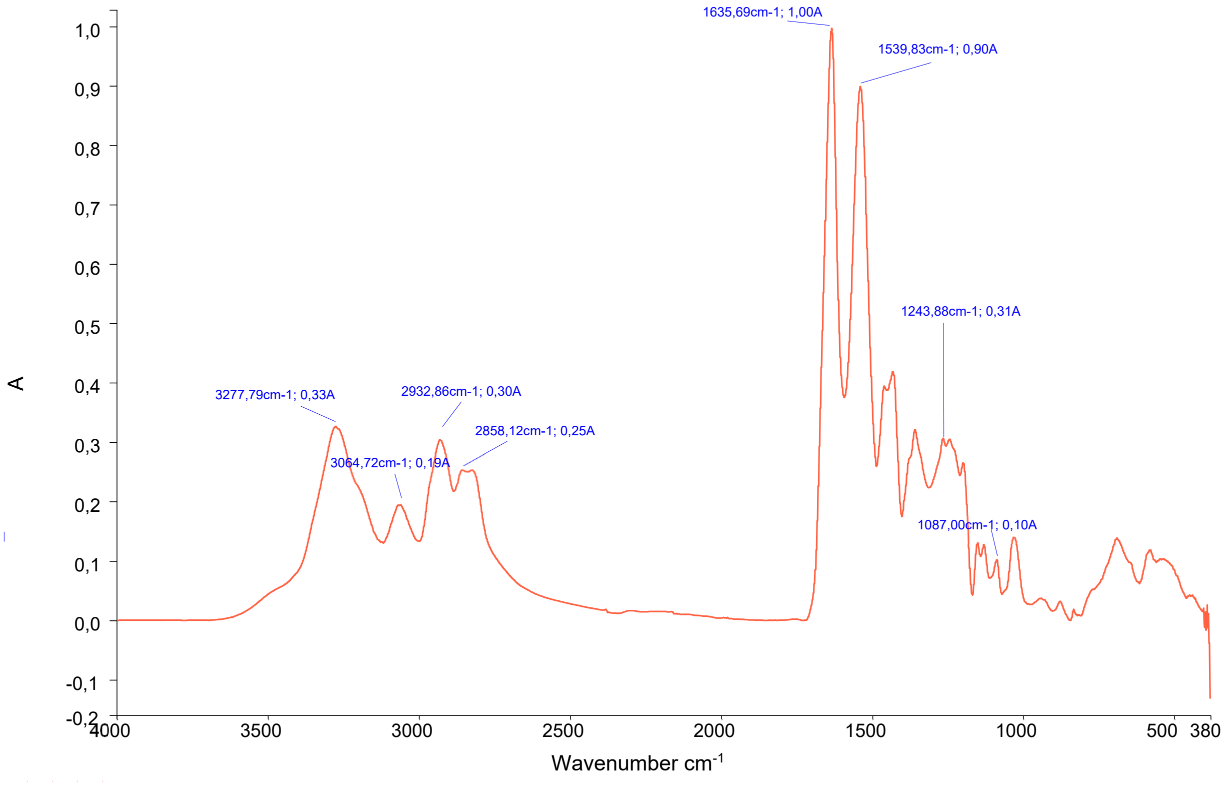

Figure S20. FTIR-ATR spectrum of **G3-alt**.

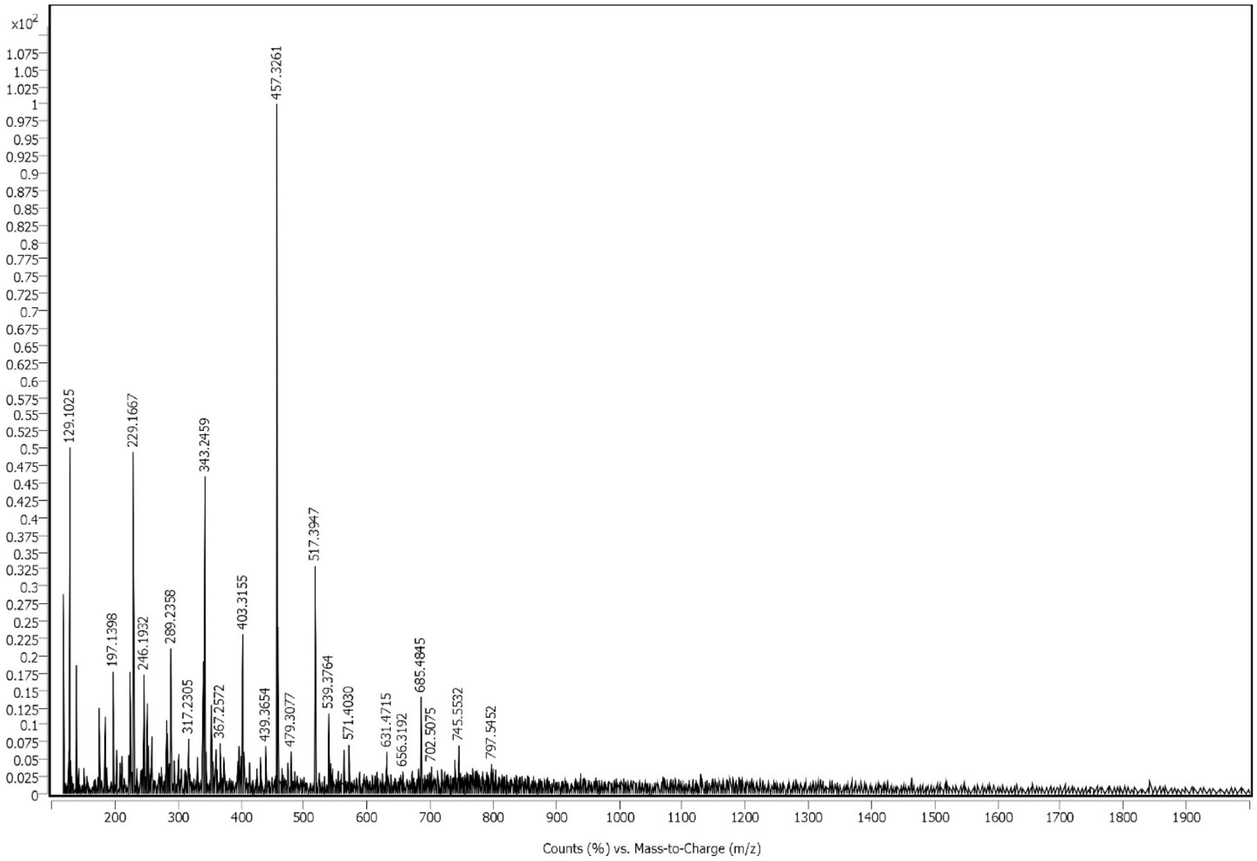

Figure S21. Mass spectrum (HR ESI) of **G3-alt**.

3. Complexation investigation

3.1. UV-Vis spectra

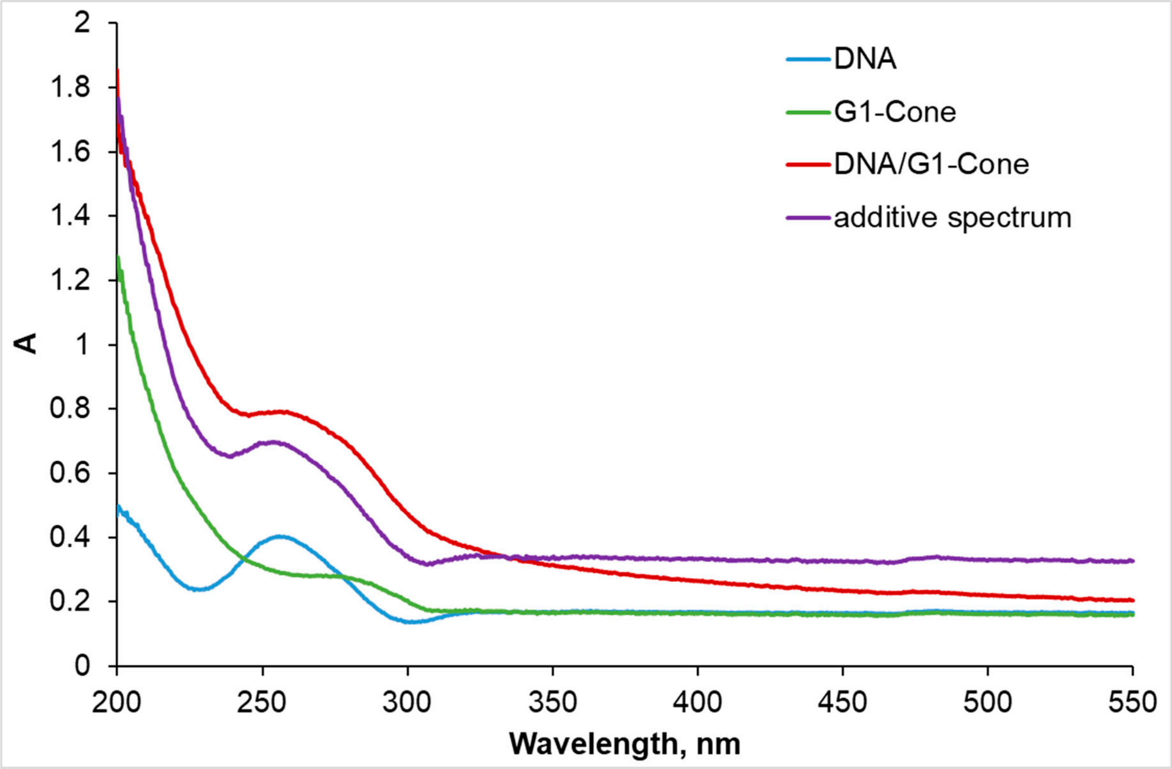

Figure S22. UV-Vis spectra of DNA (20 μM in nucleotide base pairs), **G1-cone** (10 μM), and their mixture (10 mM Tris-HCl, pH = 7.4, 293 K).

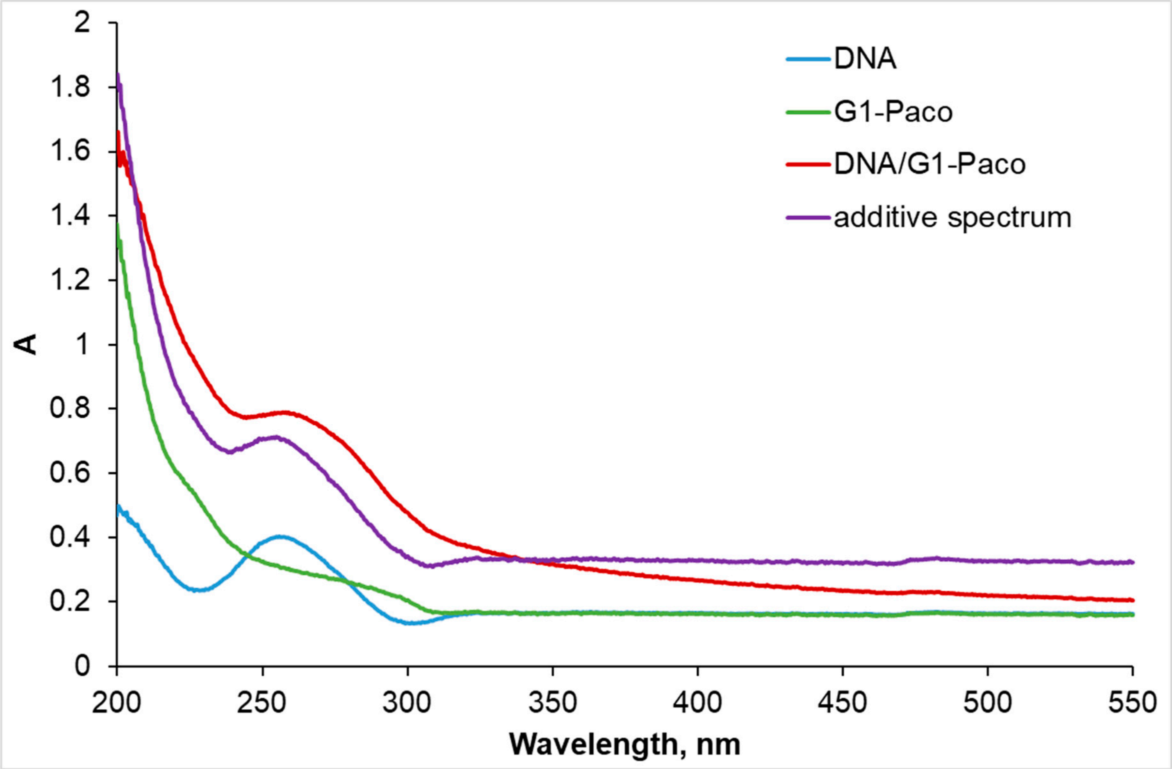

Figure S23. UV-Vis spectra of DNA (20 μM in nucleotide base pairs), **G1-paco** (10 μM), and their mixture (10 mM Tris-HCl, pH = 7.4, 293 K).

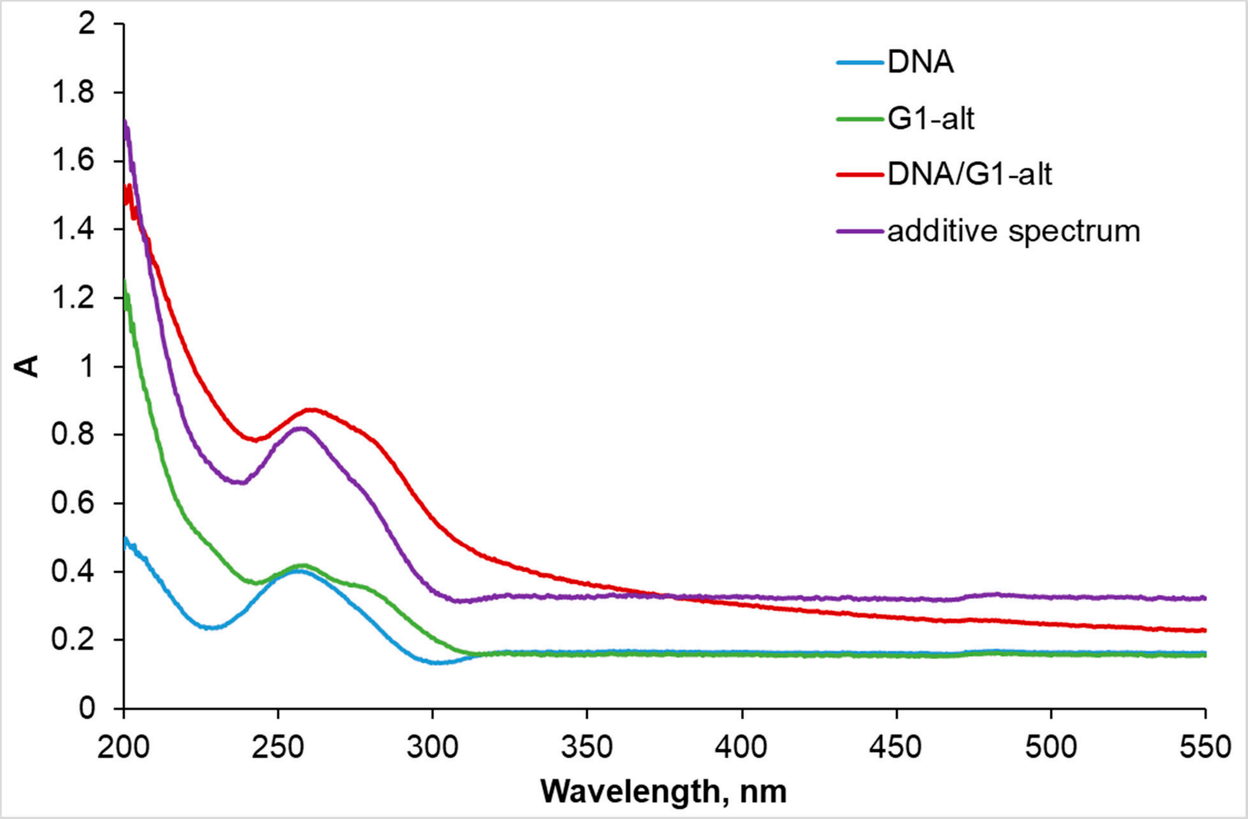

Figure S24. UV-Vis spectra of DNA (20 μM in nucleotide base pairs), **G1-alt** (10 μM), and their mixture (10 mM Tris-HCl, pH = 7.4, 293 K).

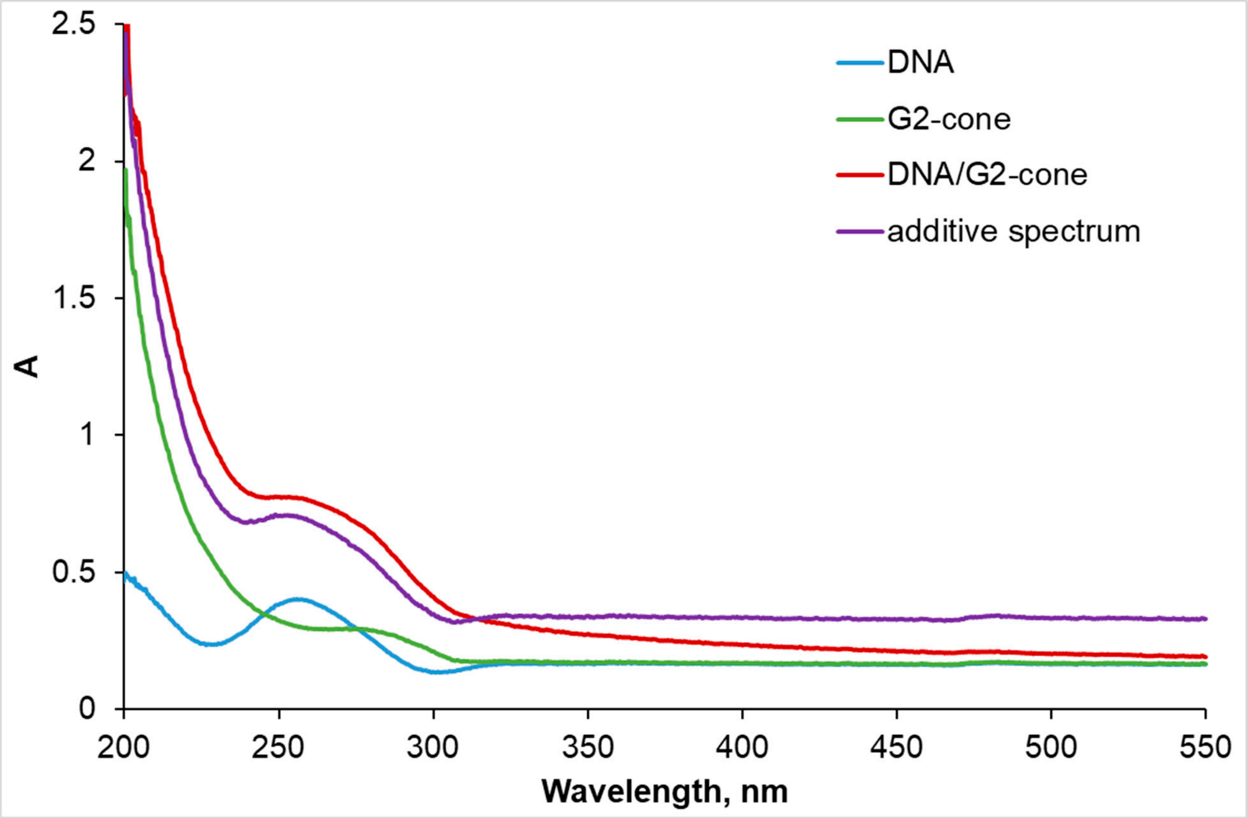

Figure S25. UV-Vis spectra of DNA (20 μM in nucleotide base pairs), **G2-cone** (10 μM), and their mixture (10 mM Tris-HCl, pH = 7.4, 293 K).

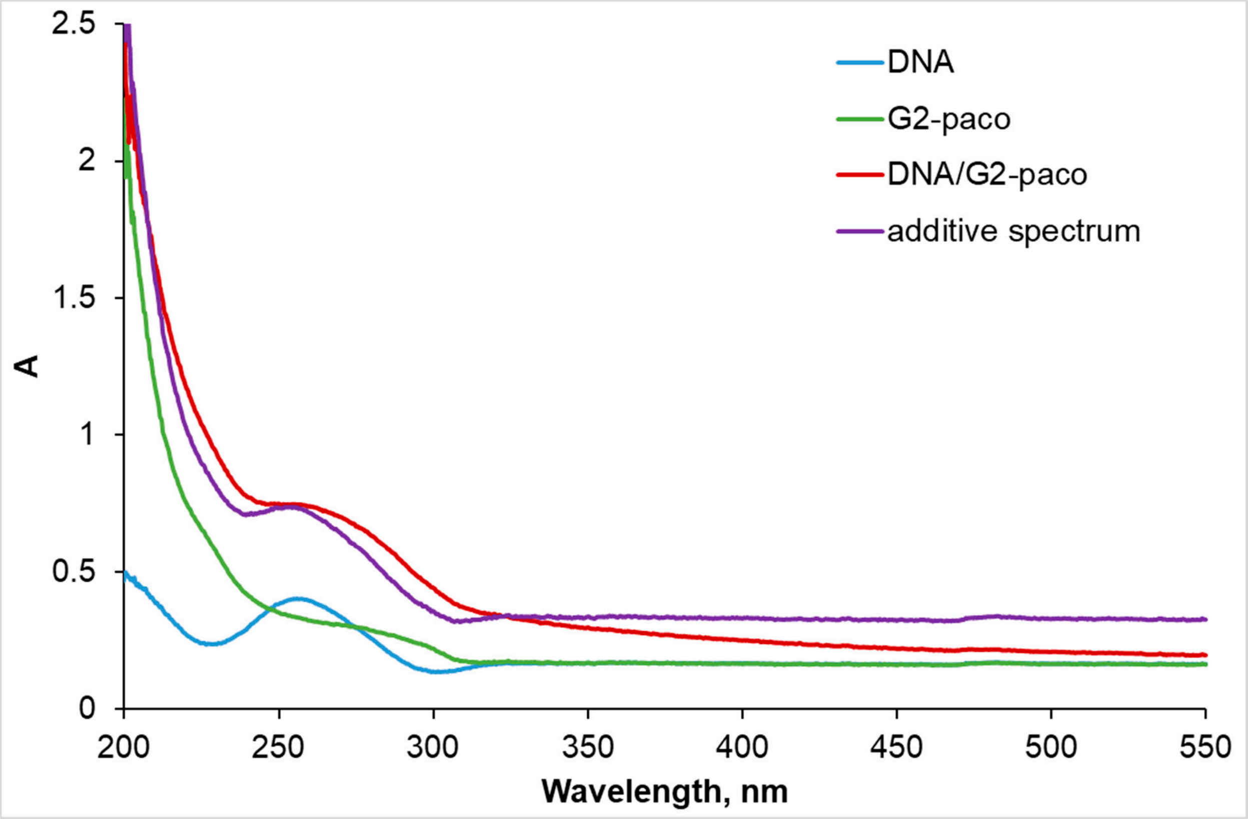

Figure S26. UV-Vis spectra of DNA (20 μM in nucleotide base pairs), **G2-paco** (10 μM), and their mixture (10 mM Tris-HCl, pH = 7.4, 293 K).

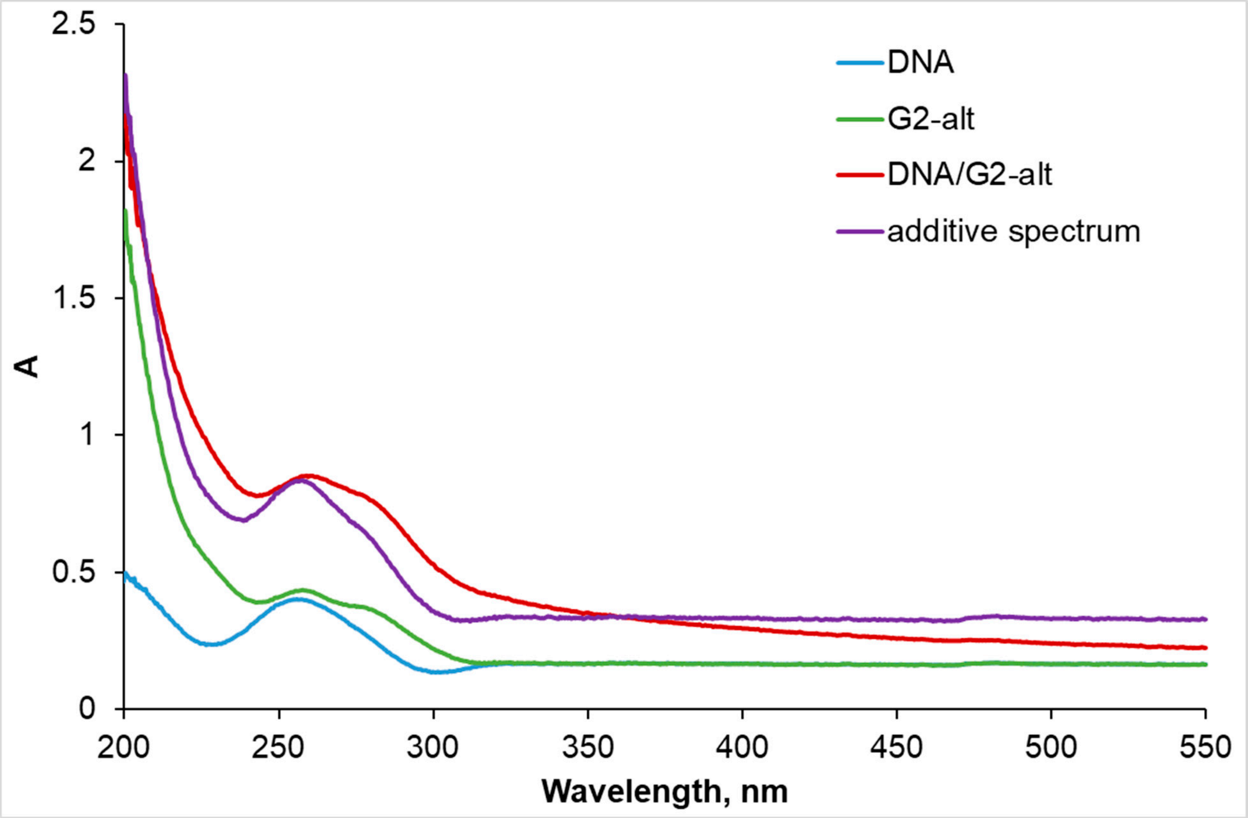

Figure S27. UV-Vis spectra of DNA (20 μM in nucleotide base pairs), **G2-alt** (10 μM), and their mixture (10 mM Tris-HCl, pH = 7.4, 293 K).

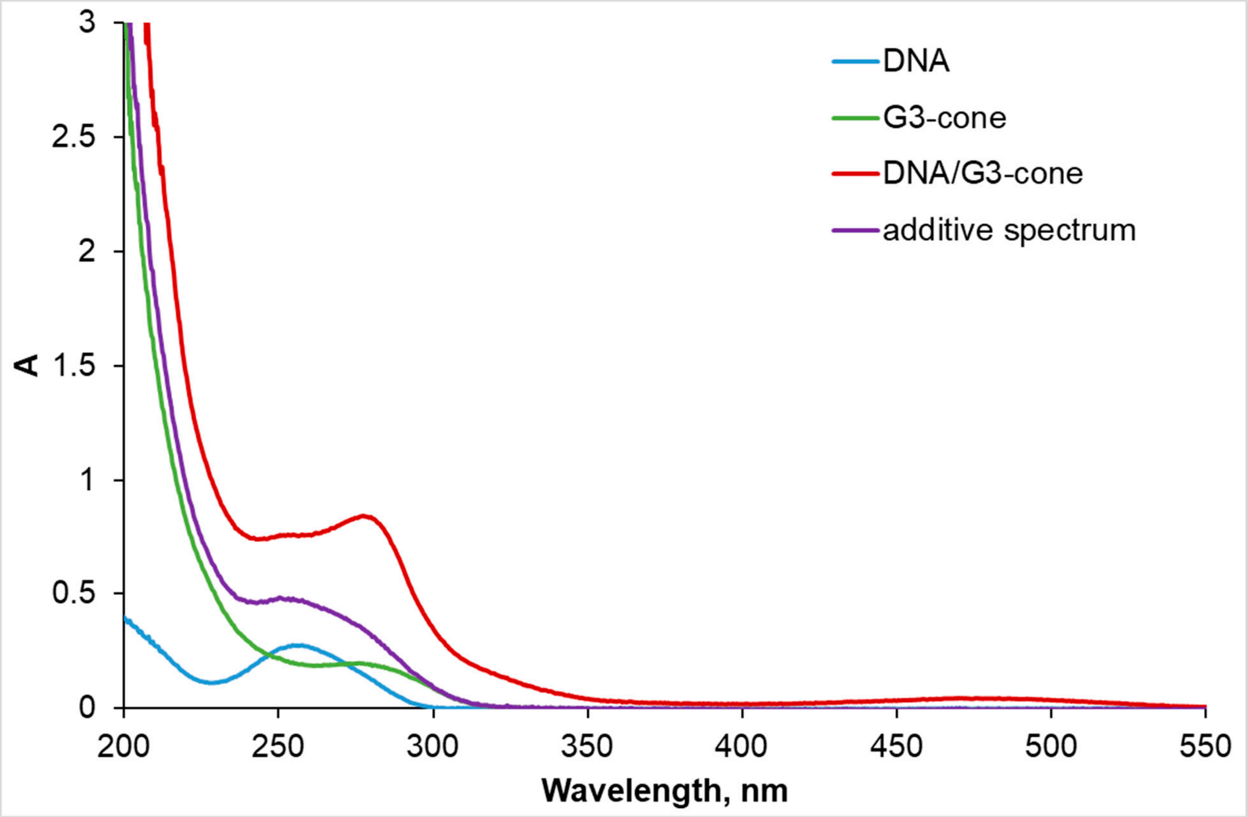

Figure S28. UV-Vis spectra of DNA (20 μM in nucleotide base pairs), **G3-cone** (10 μM), and their mixture (10 mM Tris-HCl, pH = 7.4, 293 K).

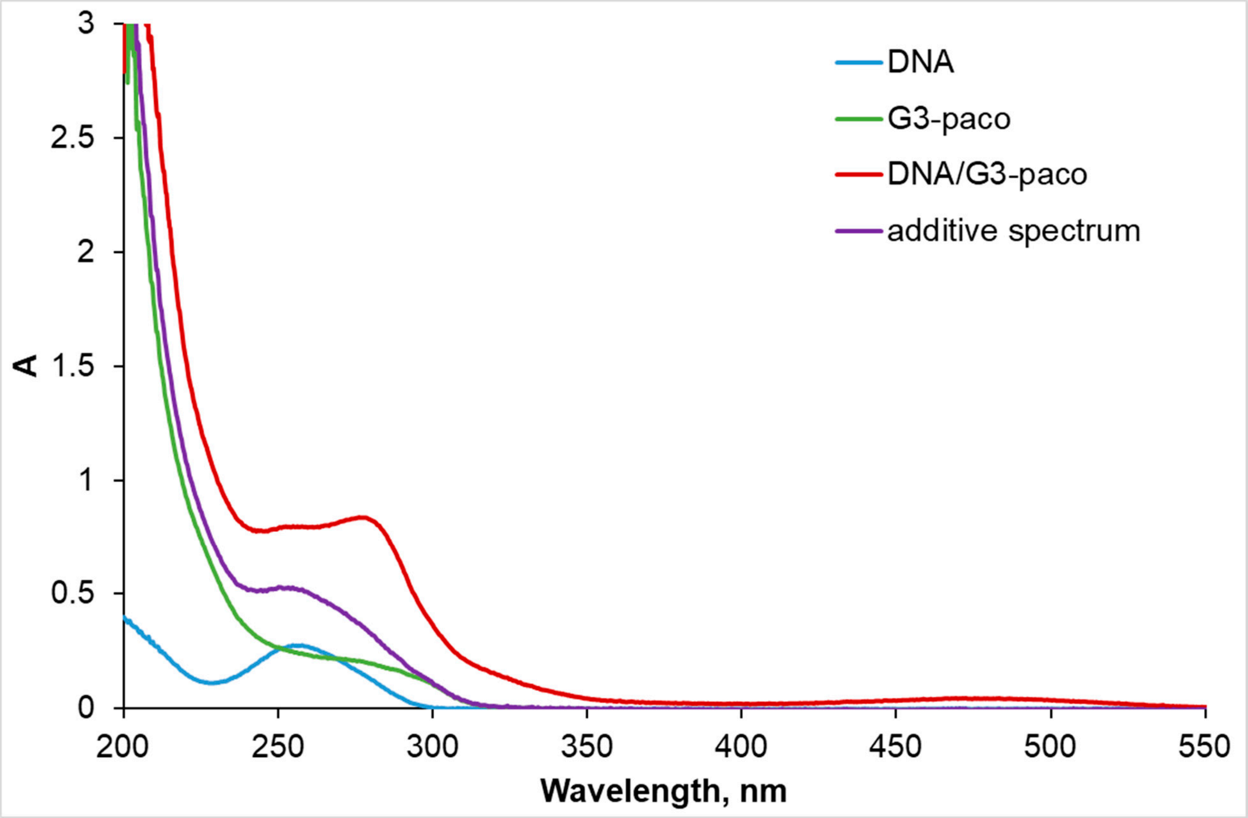

Figure S29. UV-Vis spectra of DNA (20 μM in nucleotide base pairs), **G3-paco** (10 μM), and their mixture (10 mM Tris-HCl, pH = 7.4, 293 K).

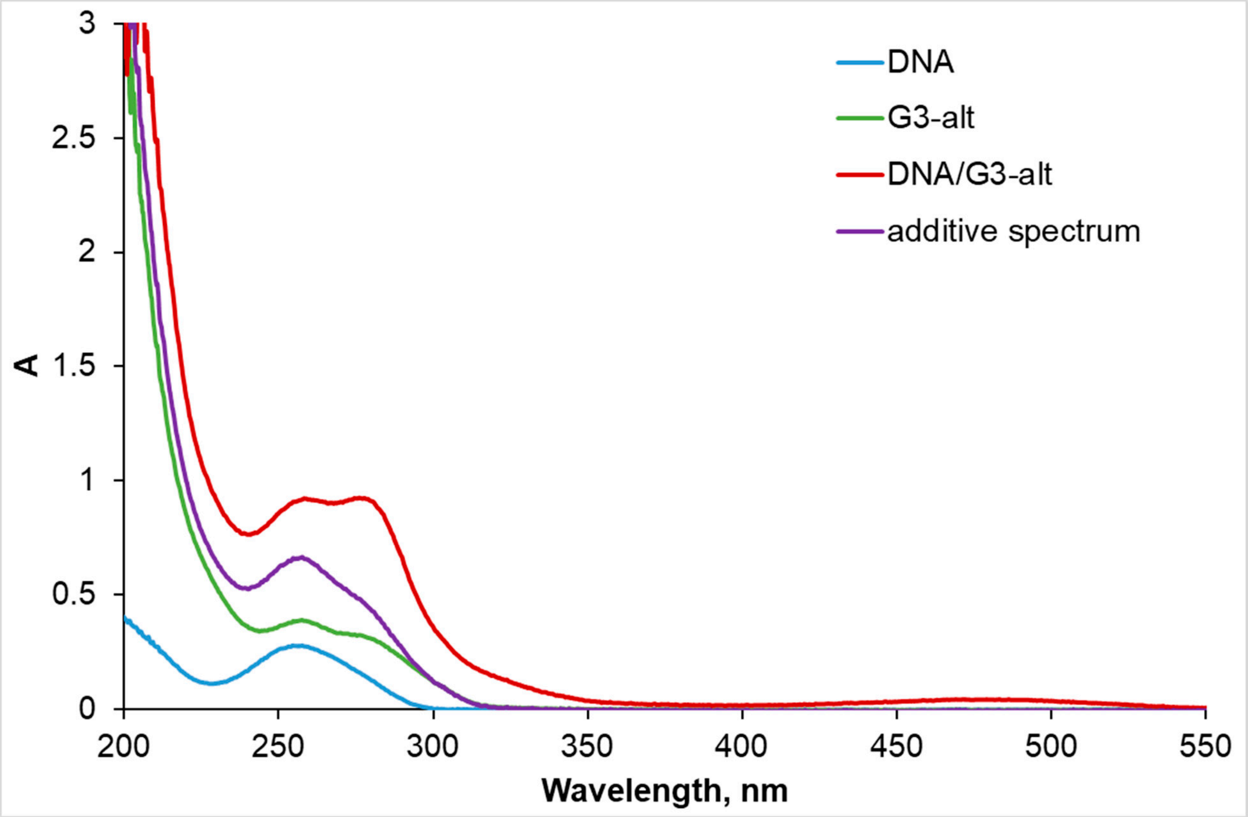

Figure S30. UV-Vis spectra of DNA (20 μM in nucleotide base pairs), **G3-alt** (10 μM), and their mixture (10 mM Tris-HCl, pH = 7.4, 293 K).

3.2. Fluorescence spectroscopy data

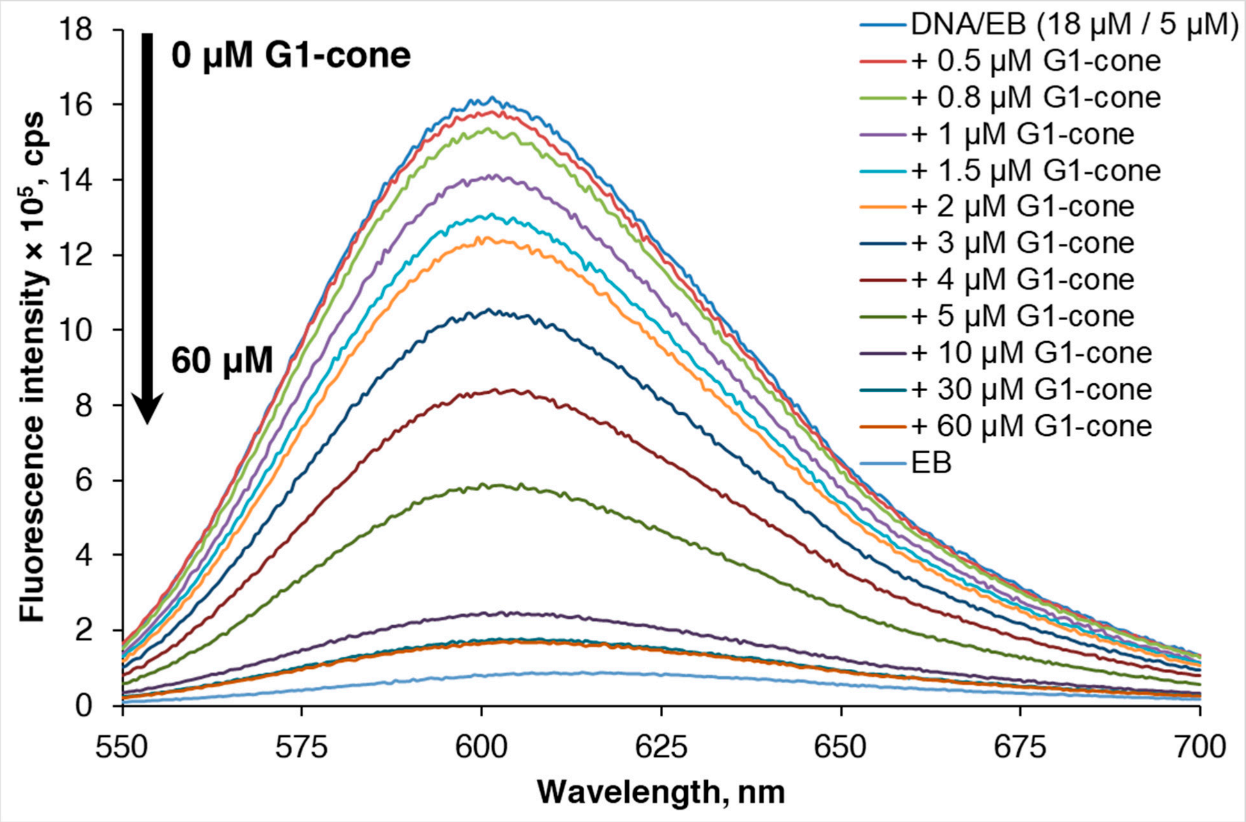

Figure S31. Fluorescence spectra of the DNA/EB complex (18 µM in nucleotide base pairs / 5 µM) in the presence of **G1-cone** (0–60 µM) (10 mM Tris-HCl, pH = 7.4, 293 K). λ_{ex} = 525 nm.

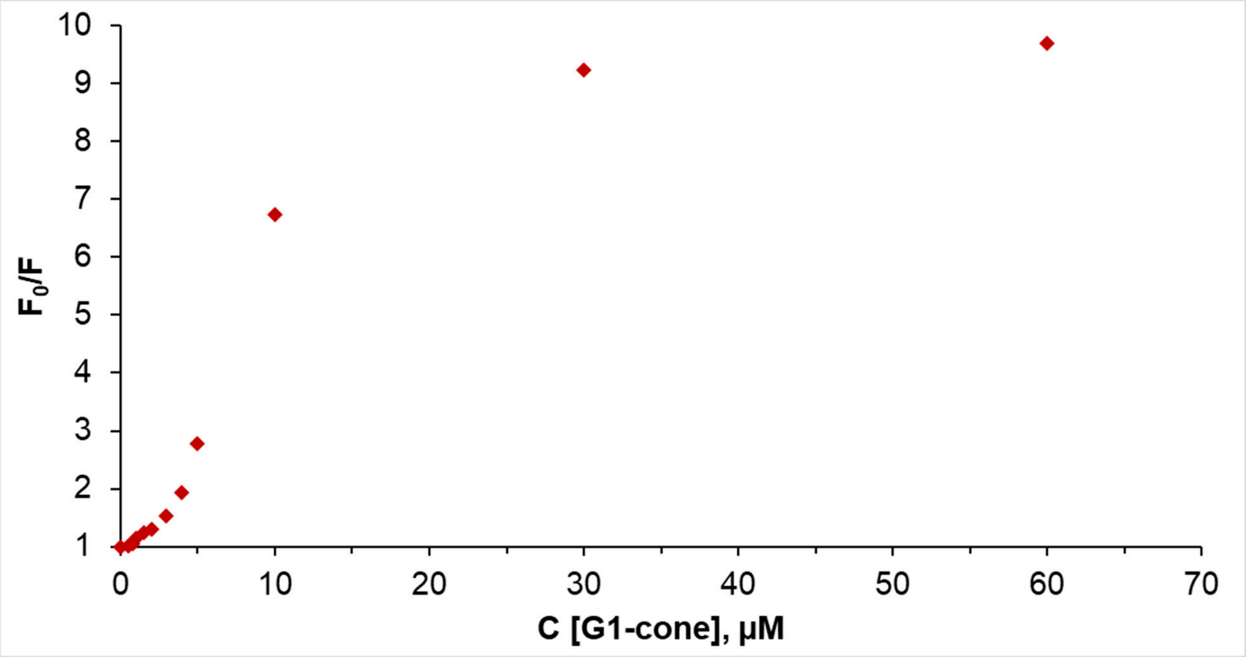

Figure S32. Stern-Volmer plot of the DNA/EB/**G1-cone** system (10 mM Tris-HCl, pH = 7.4, 293 K).

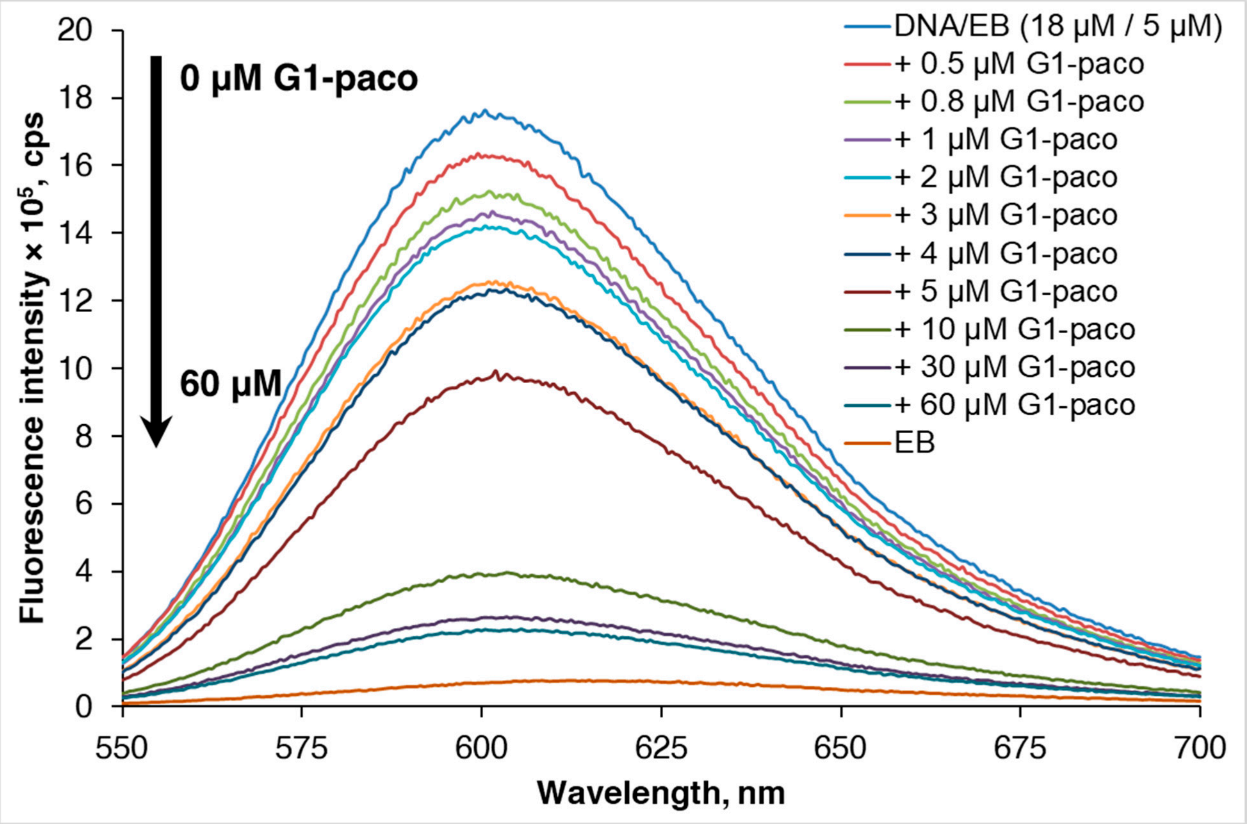

Figure S33. Fluorescence spectra of the DNA/EB complex (18 μM in nucleotide base pairs / 5 μM) in the presence of **G1-paco** (0–60 μM) (10 mM Tris-HCl, pH = 7.4, 293 K). λ_{ex} = 525 nm.

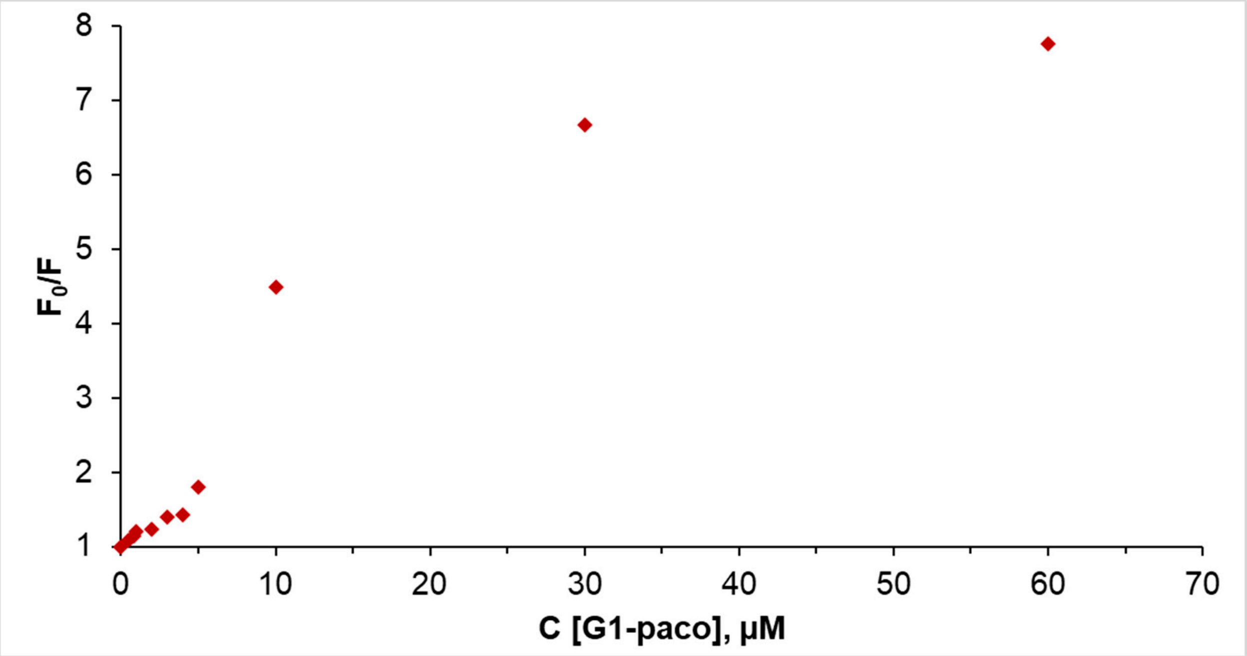

Figure S34. Stern-Volmer plot of the DNA/EB/**G1-paco** system (10 mM Tris-HCl, pH = 7.4, 293 K).

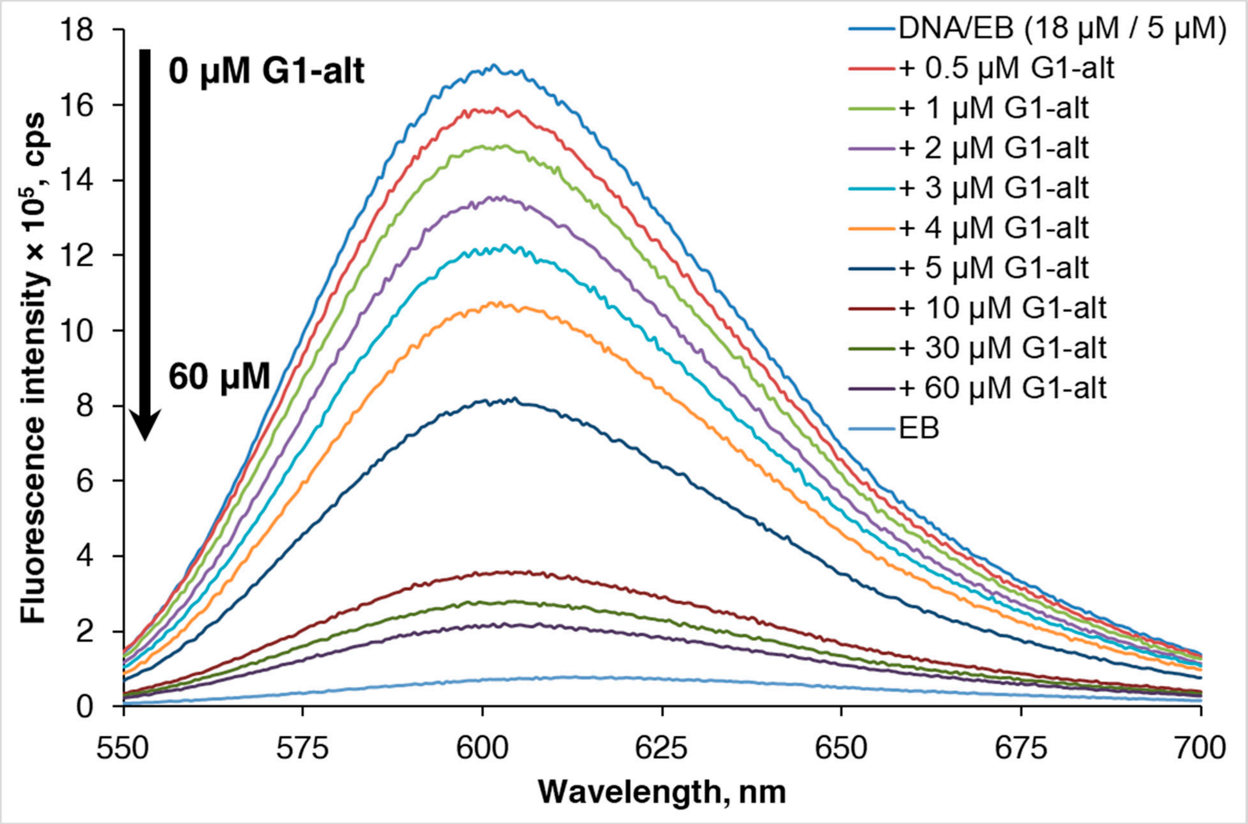

Figure S35. Fluorescence spectra of the DNA/EB complex (18 μM in nucleotide base pairs / 5 μM) in the presence of **G1-alt** (0–60 μM) (10 mM Tris-HCl, pH = 7.4, 293 K). λ_{ex} = 525 nm.

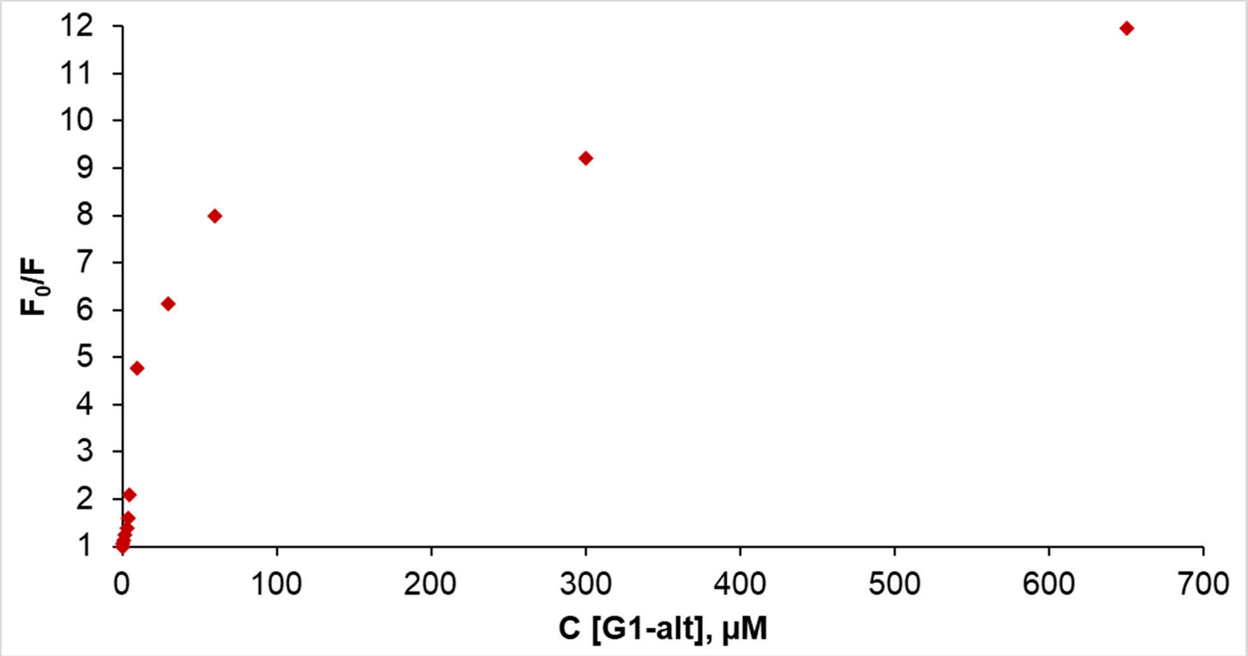

Figure S36. Stern-Volmer plot of the DNA/EB/**G1-alt** system (10 mM Tris-HCl, pH = 7.4, 293 K).

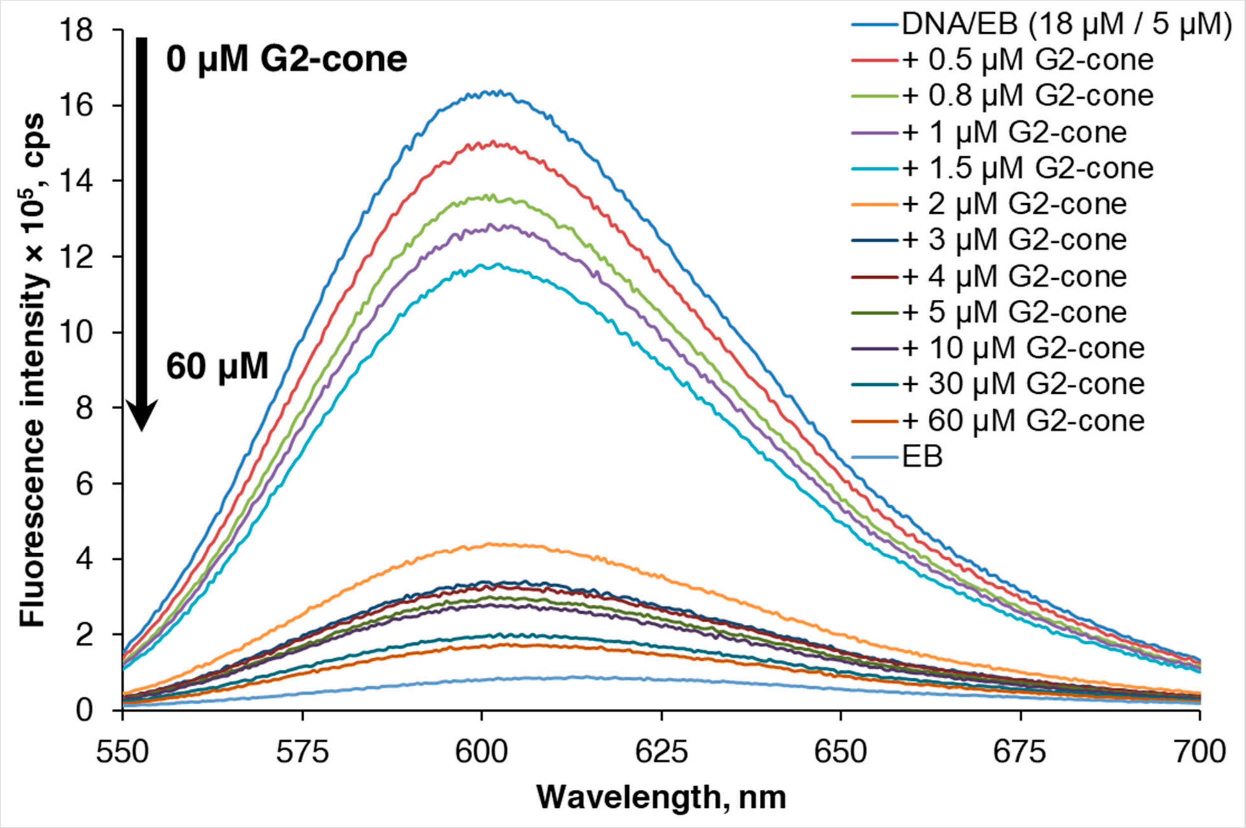

Figure S37. Fluorescence spectra of the DNA/EB complex (18 μM in nucleotide base pairs / 5 μM) in the presence of **G2-cone** (0–60 μM) (10 mM Tris-HCl, pH = 7.4, 293 K). λ_{ex} = 525 nm.

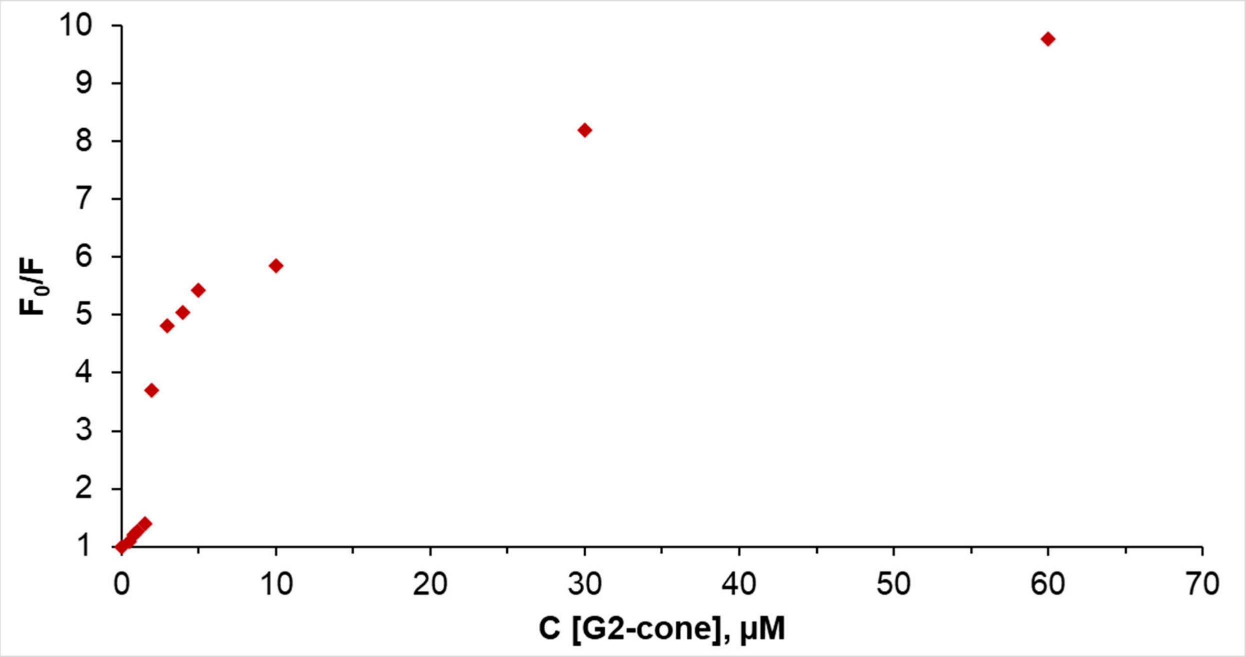

Figure S38. Stern-Volmer plot of the DNA/EB/**G2-cone** system (10 mM Tris-HCl, pH = 7.4, 293 K).

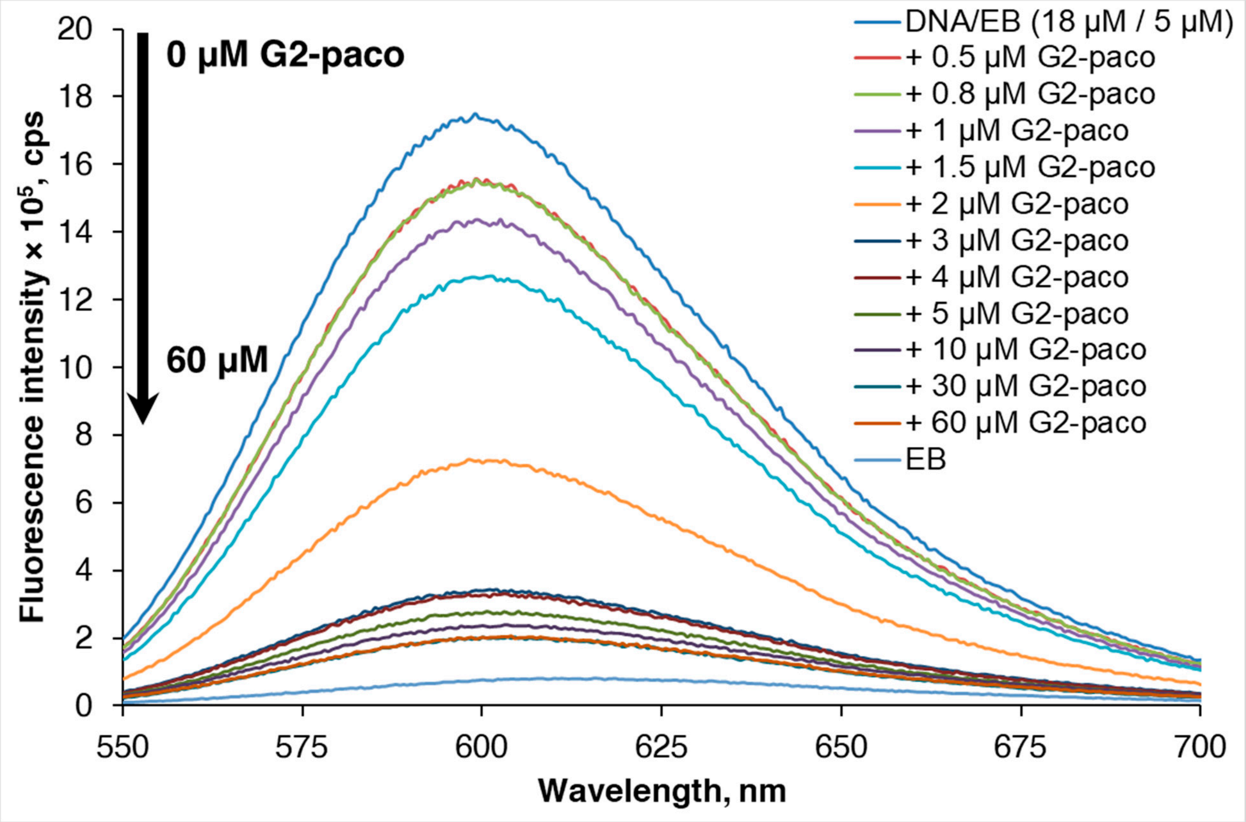

Figure S39. Fluorescence spectra of the DNA/EB complex (18 μM in nucleotide base pairs / 5 μM) in the presence of **G2-paco** (0–60 μM) (10 mM Tris-HCl, pH = 7.4, 293 K). λ_{ex} = 525 nm.

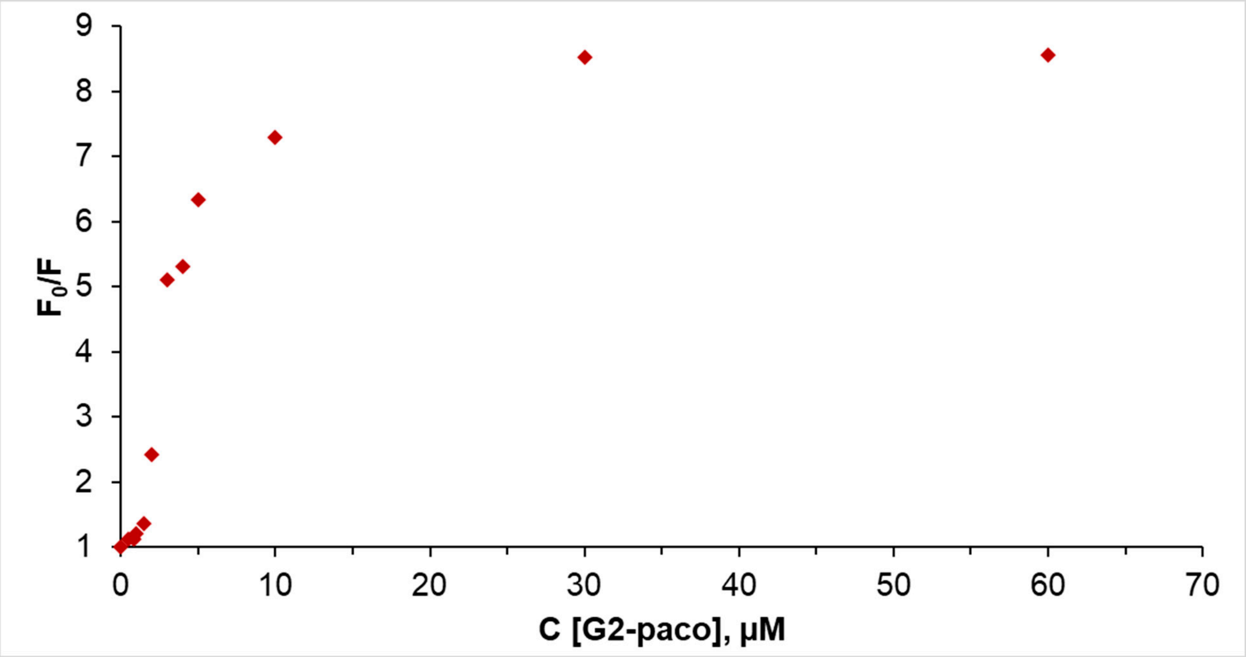

Figure S40. Stern-Volmer plot of the DNA/EB/**G2-paco** system (10 mM Tris-HCl, pH = 7.4, 293 K).

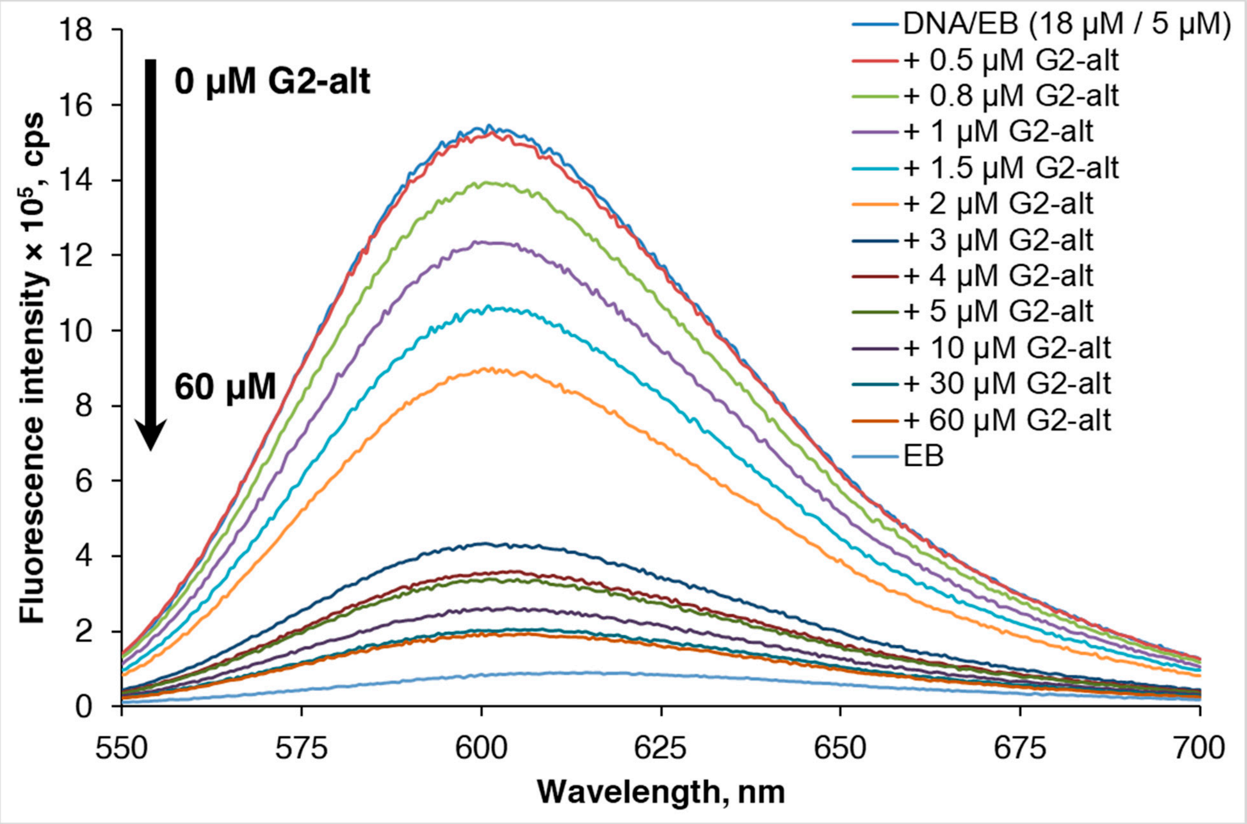

Figure S41. Fluorescence spectra of the DNA/EB complex (18 μM in nucleotide base pairs / 5 μM) in the presence of **G2-alt** (0–60 μM) (10 mM Tris-HCl, pH = 7.4, 293 K). λ_{ex} = 525 nm.

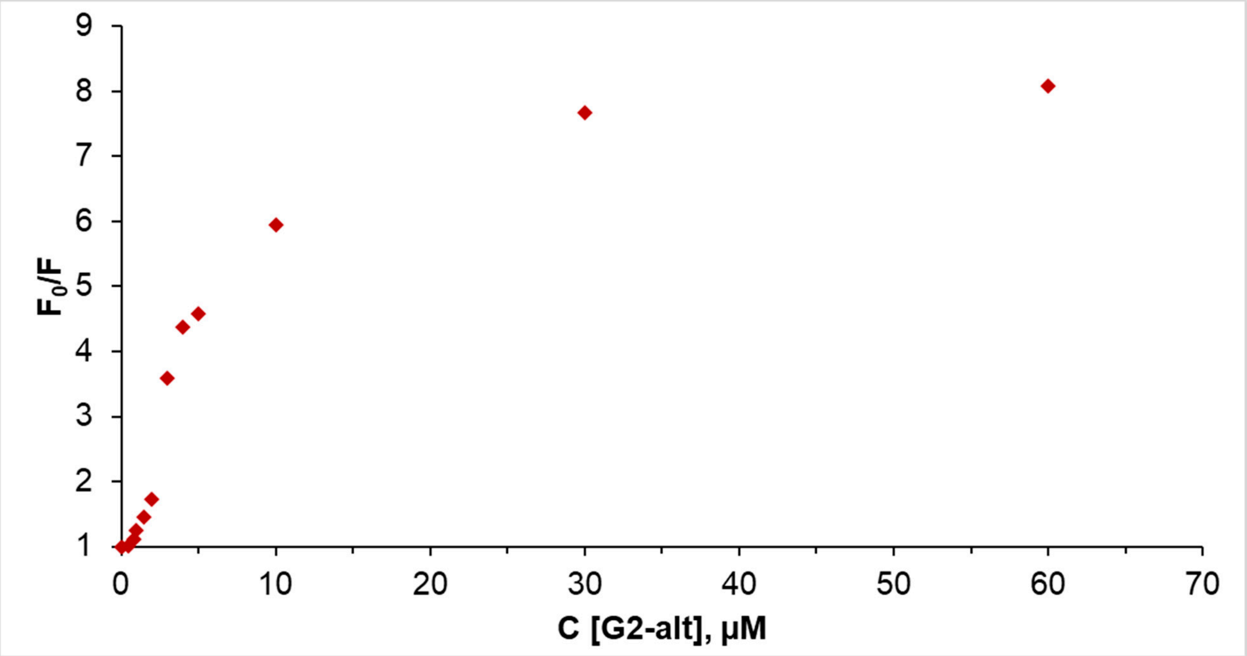

Figure S42. Stern-Volmer plot of the DNA/EB/**G2-alt** system (10 mM Tris-HCl, pH = 7.4, 293 K).

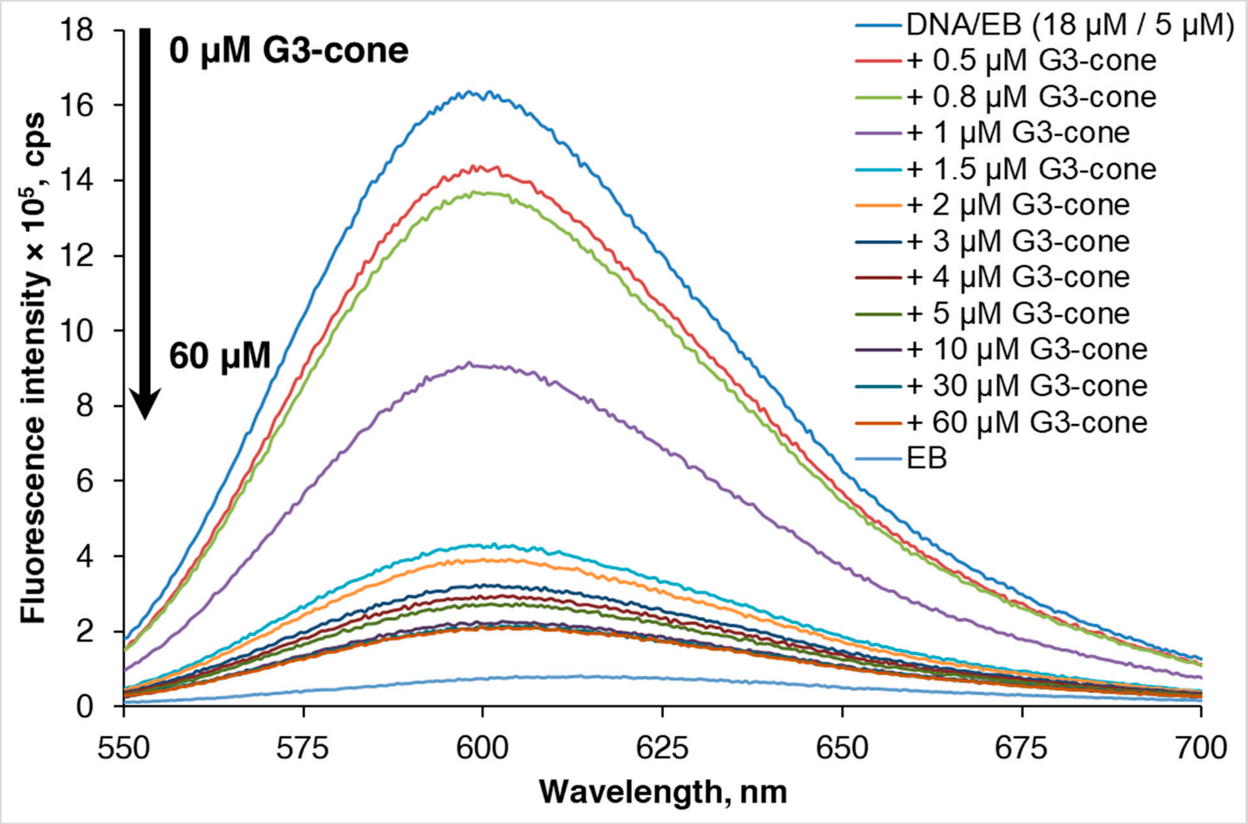

Figure S43. Fluorescence spectra of the DNA/EB complex (18 μM in nucleotide base pairs / 5 μM) in the presence of **G3-cone** (0–60 μM) (10 mM Tris-HCl, pH = 7.4, 293 K). λ_{ex} = 525 nm.

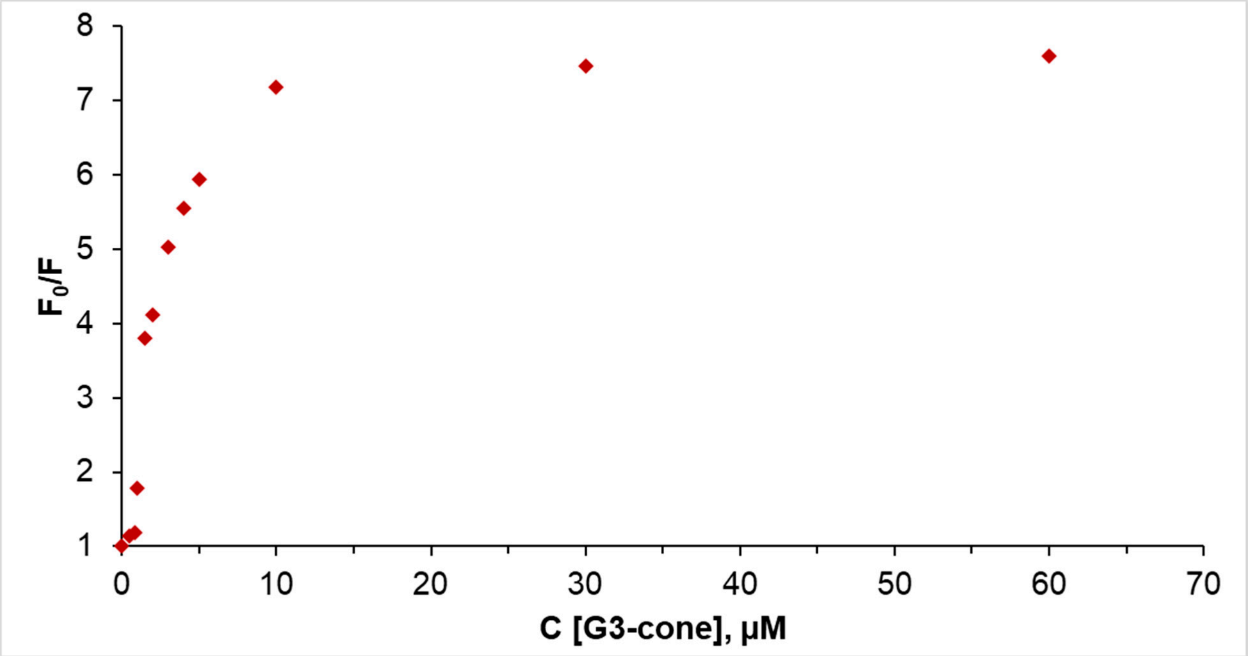

Figure S44. Stern-Volmer plot of the DNA/EB/**G3-cone** system (10 mM Tris-HCl, pH = 7.4, 293 K).

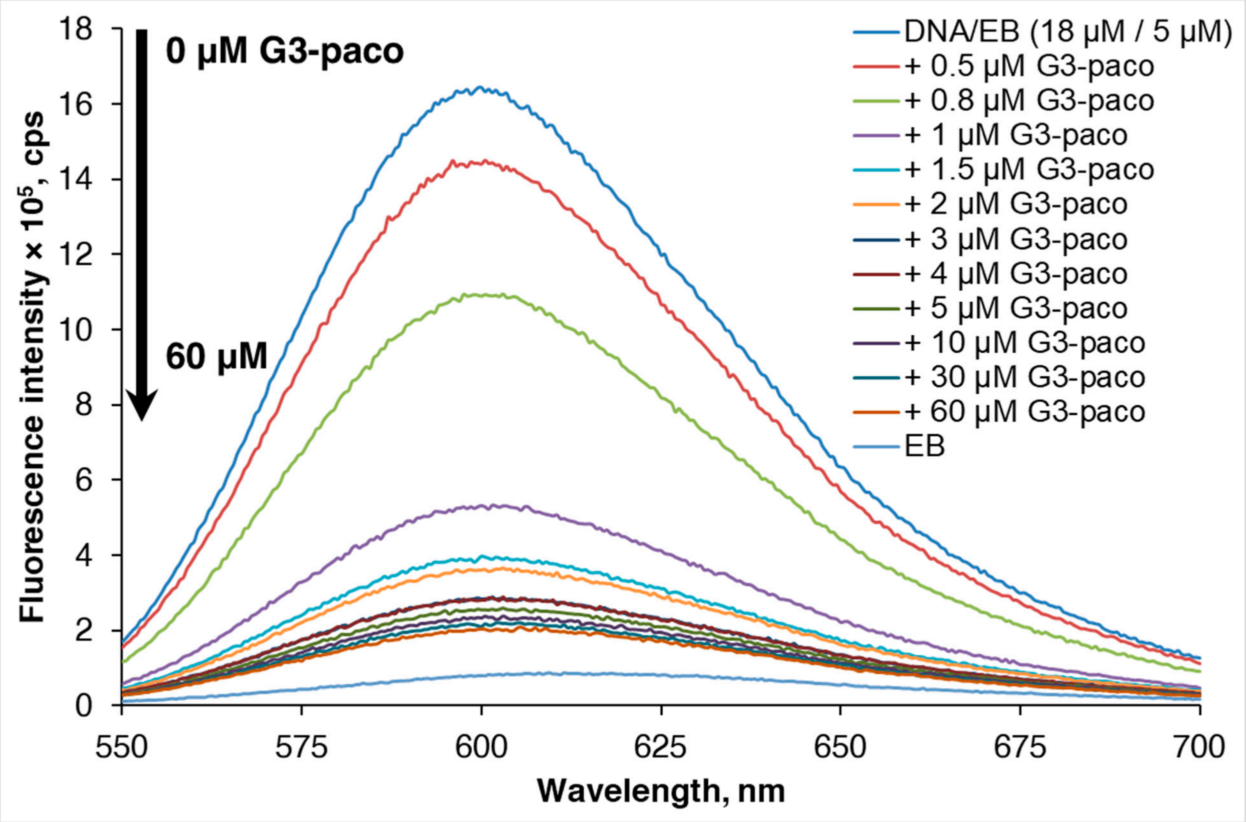

Figure S45. Fluorescence spectra of the DNA/EB complex (18 μM in nucleotide base pairs / 5 μM) in the presence of **G3-paco** (0–60 μM) (10 mM Tris-HCl, pH = 7.4, 293 K). λ_{ex} = 525 nm.

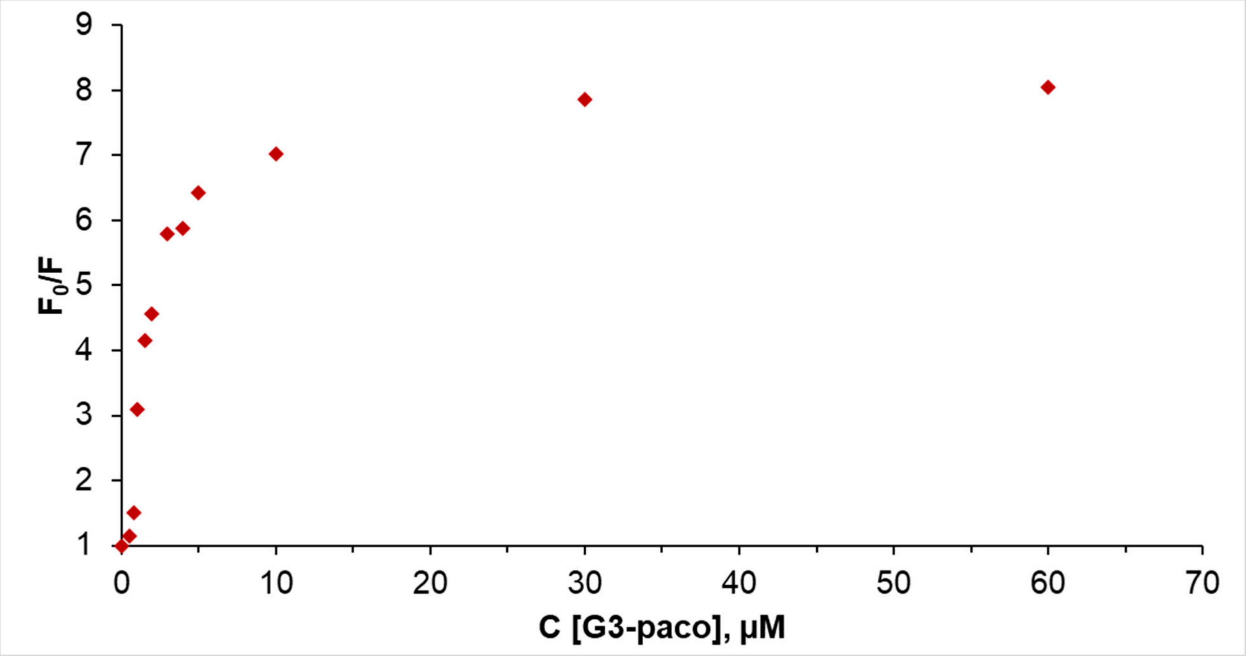

Figure S46. Stern-Volmer plot of the DNA/EB/**G3-paco** system (10 mM Tris-HCl, pH = 7.4, 293 K).

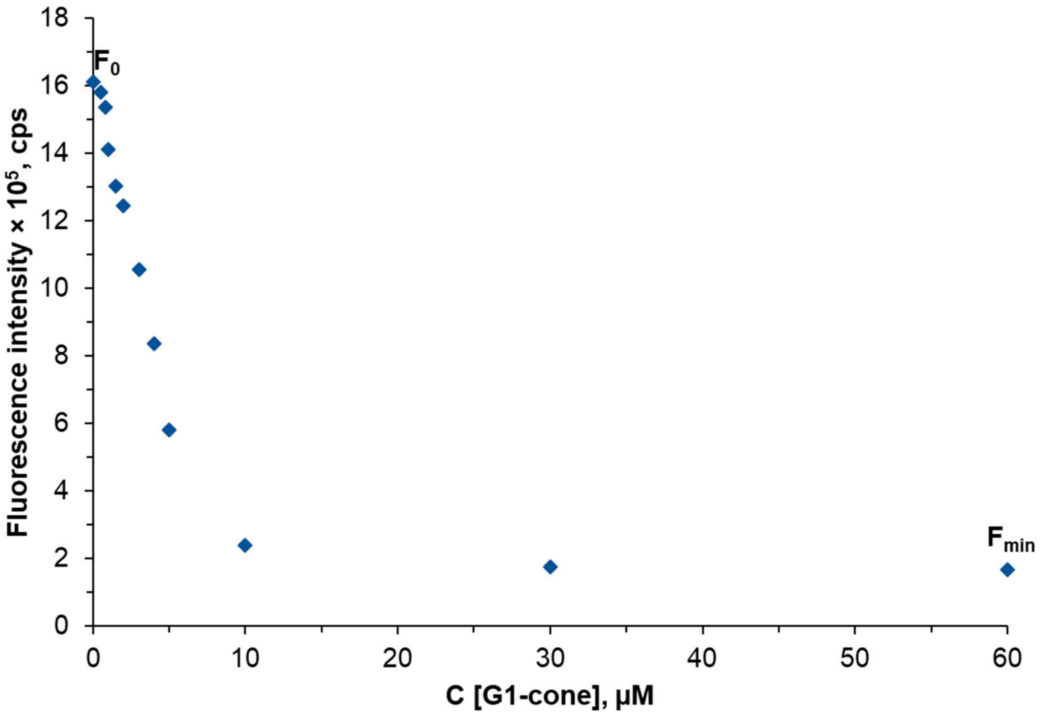

Figure S47. Determination of C_{50} by titration curve of the DNA/EB complex (18 μM in nucleotide base pairs / 5 μM) in the presence of **G1-cone** (0–60 μM) (10 mM Tris-HCl, pH = 7.4, 293 K).

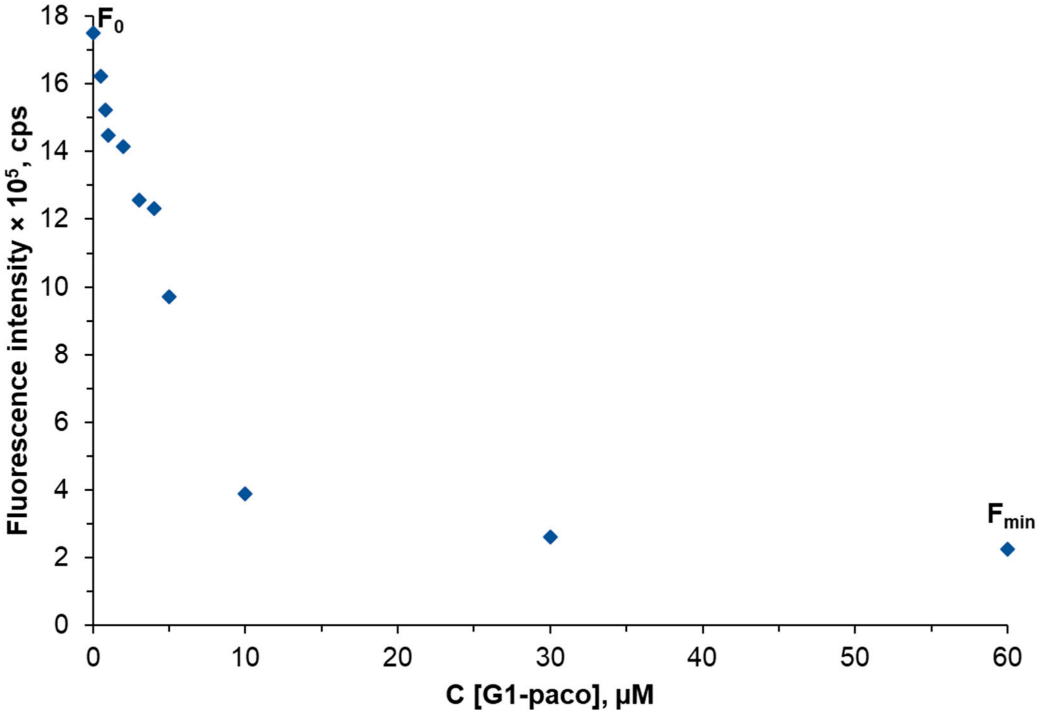

Figure S48. Determination of C_{50} by titration curve of the DNA/EB complex (18 μM in nucleotide base pairs / 5 μM) in the presence of **G1-paco** (0–60 μM) (10 mM Tris-HCl, pH = 7.4, 293 K).

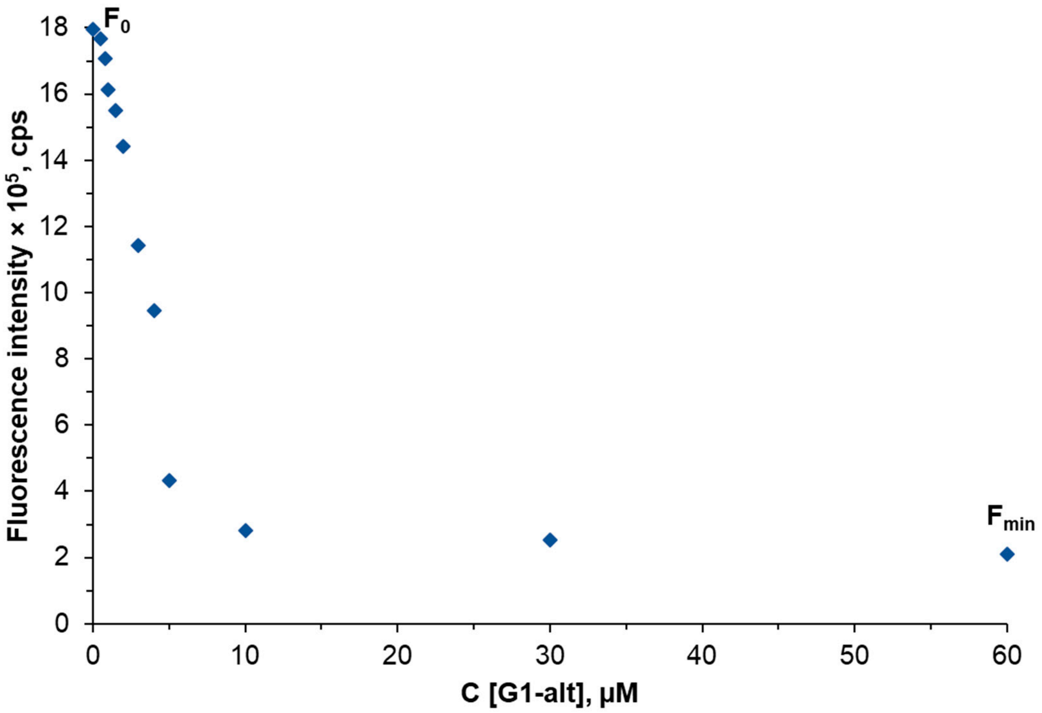

Figure S49. Determination of C_{50} by titration curve of the DNA/EB complex (18 μM in nucleotide base pairs / 5 μM) in the presence of **G1-alt** (0–60 μM) (10 mM Tris-HCl, pH = 7.4, 293 K).

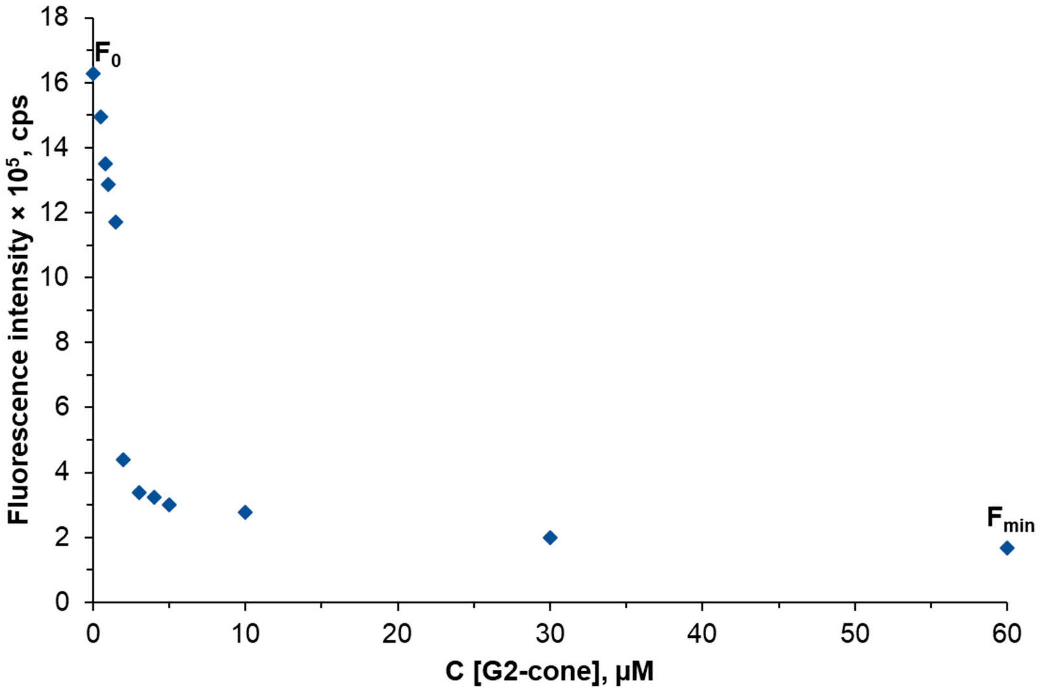

Figure S50. Determination of C_{50} by titration curve of the DNA/EB complex (18 μM in nucleotide base pairs / 5 μM) in the presence of **G2-cone** (0–60 μM) (10 mM Tris-HCl, pH = 7.4, 293 K).

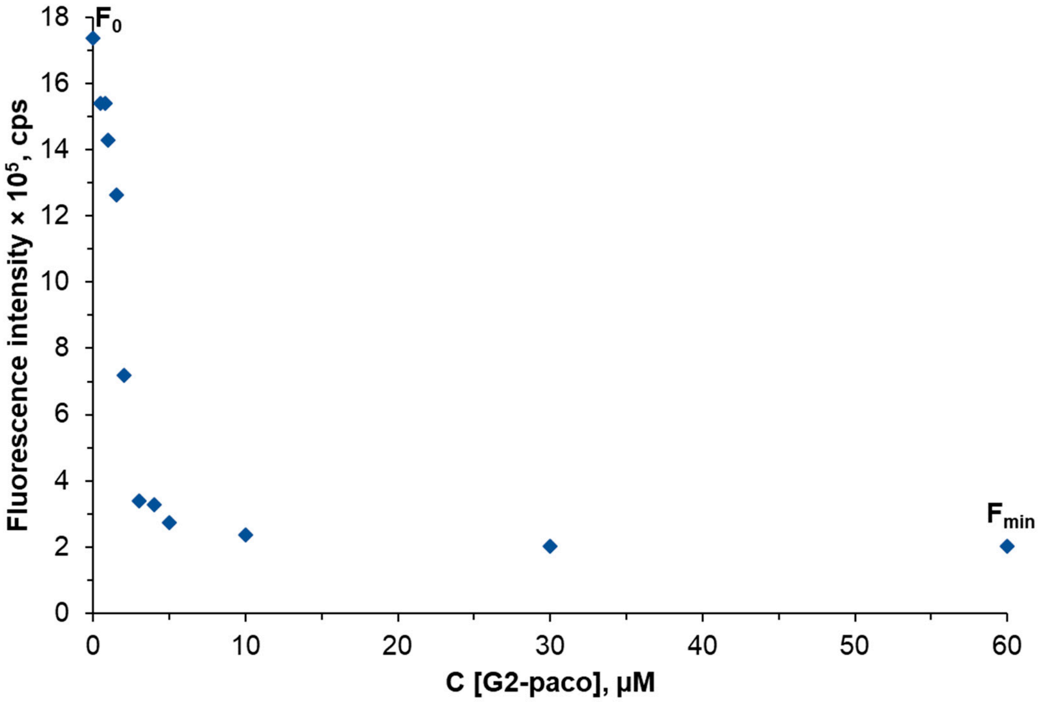

Figure S51. Determination of C_{50} by titration curve of the DNA/EB complex (18 μM in nucleotide base pairs / 5 μM) in the presence of **G2-paco** (0–60 μM) (10 mM Tris-HCl, pH = 7.4, 293 K).

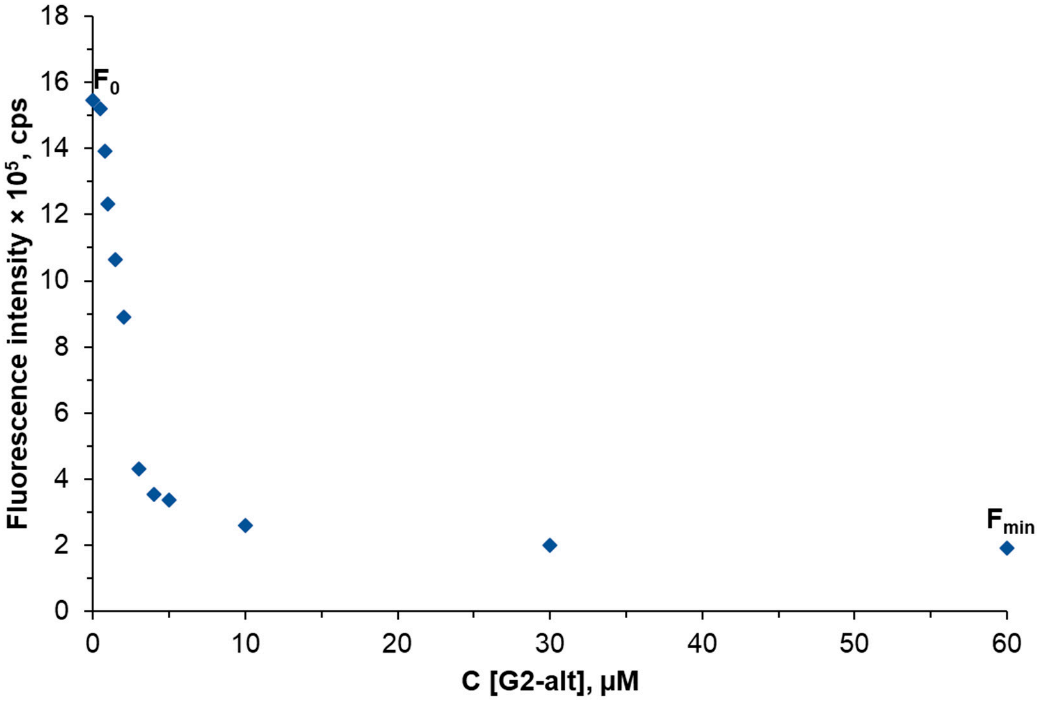

Figure S52. Determination of C_{50} by titration curve of the DNA/EB complex (18 μM in nucleotide base pairs / 5 μM) in the presence of **G2-alt** (0–60 μM) (10 mM Tris-HCl, pH = 7.4, 293 K).

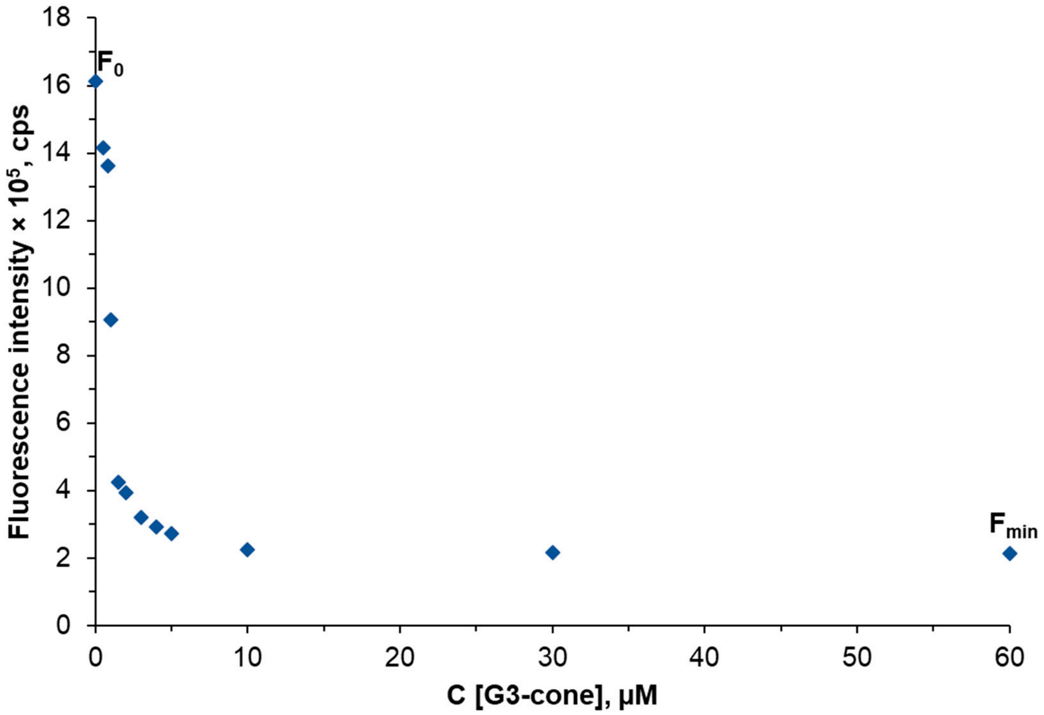

Figure S53. Determination of C_{50} by titration curve of the DNA/EB complex (18 μM in nucleotide base pairs / 5 μM) in the presence of **G3-cone** (0–60 μM) (10 mM Tris-HCl, pH = 7.4, 293 K).

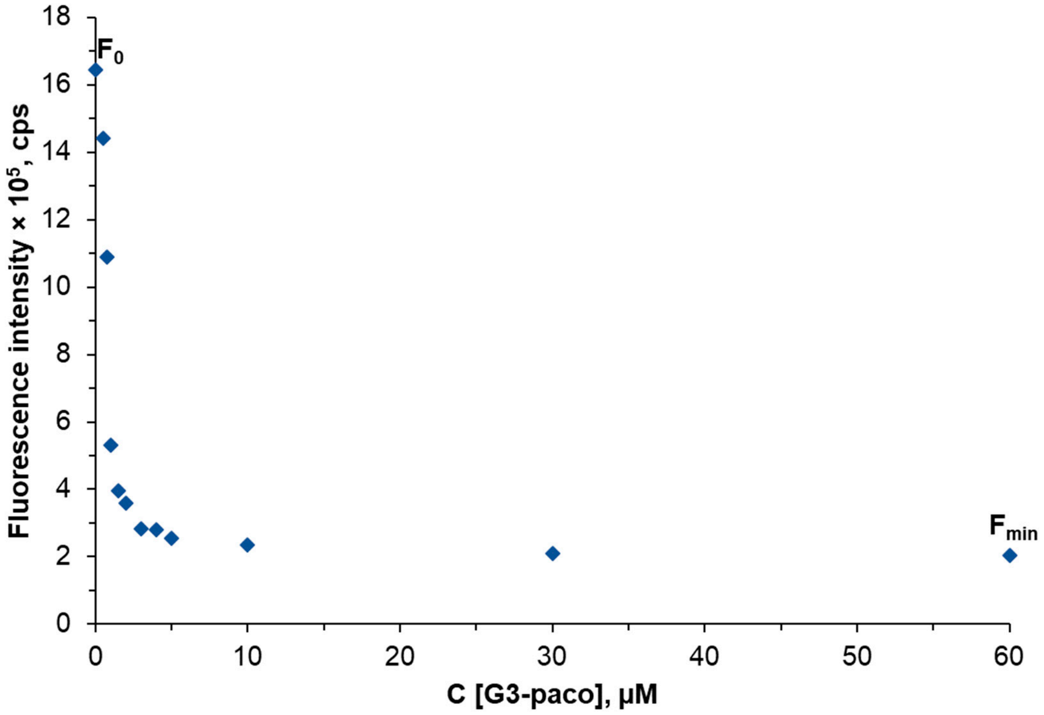

Figure S54. Determination of C_{50} by titration curve of the DNA/EB complex (18 μM in nucleotide base pairs / 5 μM) in the presence of **G3-paco** (0–60 μM) (10 mM Tris-HCl, pH = 7.4, 293 K).

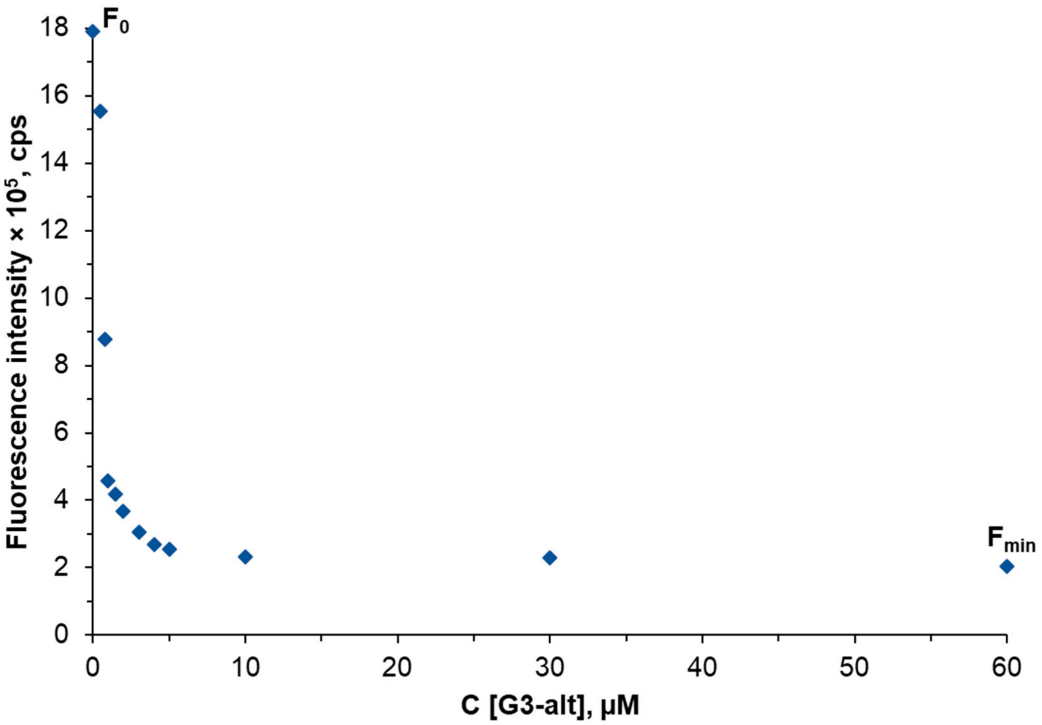

Figure S55. Determination of C_{50} by titration curve of the DNA/EB complex (18 μM in nucleotide base pairs / 5 μM) in the presence of **G3-alt** (0–60 μM) (10 mM Tris-HCl, pH = 7.4, 293 K).

3.3. DLS data

Table S1. Characteristics of light scattering (average hydrodynamic diameter of associates *d*, polydispersity index PDI) of the particles obtained by **G1 PAMAM-calix-dendrimers** and calf thymus DNA in a buffer solution (Tris-HCl buffer, 10 mM, pH 7.4).

System	C, μM	d, nm	PDI
calf thymus DNA	20	450±88.4	0.49±0.12
**G1-cone**	500	6.8±0.24	0.27±0.05
**G1-cone**	100	100±148.5	0.42±0.13
**G1-cone**	50	445±57.25	0.50±0.13
**G1-cone**	10	320±53	0.52±0.1
**G1-cone**	5	406±63.8	0.66±0.23
**G1-cone**	1	344±38	0.49±0.09
DNA/ **G1-cone**	20/500	526±95.4	0.29±0.03
DNA / **G1-cone**	20/100	265.6±31.7	0.31±0.04
DNA/ **G1-cone**	20/50	160±5.1	0.21±0.01
DNA/ **G1-cone**	20/10	2361±1469	0.59±0.08
DNA/ **G1-cone**	20/5	1094±278	0.48±0.1
DNA/ **G1-cone**	20/1	233±26.5	0.32±0.03
**G1-paco**	500	175.1±47	0.54±0.172
**G1-paco**	100	204.8±28.6	0.39±0.164
**G1-paco**	50	207.1±24.8	0.41±0.06
**G1-paco**	10	391.1±77.02	0.61±0.1
**G1-paco**	5	459.5±231.2	0.67±0.16
**G1-paco**	1	332.1±157.3	0.53±0.06
DNA / **G1-paco**	20/500	121.1±2.2	0.19±0.06
DNA / **G1-paco**	20/100	211.9±57.2	0.27±0.04
DNA / **G1-paco**	20/50	217±14.36	0.18±0.01
DNA / **G1-paco**	20/10	1946±1239	0.47±0.05
DNA / **G1-paco**	20/5	1262±51.84	0.44±0.05
DNA / **G1-paco**	20/1	1925±982.7	0.54±0.12
**G1-alt**	500	756.6±91.7	0.50±0.06
**G1-alt**	100	1258±200.7	0.34±0.04
**G1-alt**	50	1267±100.3	0.40±0.05
**G1-alt**	10	1144±172.2	0.51±0.1
**G1-alt**	5	1116±39	0.45±0.01
**G1-alt**	1	429.7±54.93	0.57±0.11
DNA **/ G1-alt**	20/500	280.4±43.1	0.72±0.30
DNA **/ G1-alt**	20/100	3606±940.8	0.44±0.06
DNA **/ G1-alt**	20/50	3227±959.1	0.55±0.06
DNA **/ G1-alt**	20/10	1340±77.7	0.43±0.06
DNA **/ G1-alt**	20/5	644.4±40.4	0.62±0.10
DNA **/ G1-alt**	20/1	473.3±93.2	0.56±0.02

Table S2. Characteristics of light scattering (average hydrodynamic diameter of associates *d*, polydispersity index PDI) of the particles obtained by **G2 PAMAM-calix-dendrimers** and calf thymus DNA in a buffer solution (Tris-HCl buffer, 10 mM, pH 7.4).

System	C, μM	d, nm	PDI
calf thymus DNA	20	450±88.4	0.49±0.12
**G2-cone**	500	159.8±9.7	0.52±0.03
**G2-cone**	100	156.3±20.25	0.56±0.1
**G2-cone**	50	219.9±9.8	0.51±0.09
**G2-cone**	10	525.3±392.4	0.62±0.3
**G2-cone**	5	405.5±54.9	0.49±0.07
**G2-cone**	1	412.5±93.7	0.54±0.06
DNA **/ G2-cone**	20/500	499.7±162.4	0.35±0.03
DNA **/ G2-cone**	20/100	615.9±292.6	0.53±0.07
DNA **/ G2-cone**	20/50	567.4±25.2	0.30±0.02
DNA **/ G2-cone**	20/10	718.7±40.63	0.57±0.09
DNA **/ G2-cone**	20/5	962.4±533.7	0.48±0.15
DNA **/ G2-cone**	20/1	695.6±50.36	0.40±0.04
**G2-paco**	500	142.3±9.4	0.59±0.17
**G2-paco**	100	173.7±39.3	0.58±0.17
**G2-paco**	50	212.8±42.3	0.56±0.10
**G2-paco**	10	282.5±54	0.40±0.13
**G2-paco**	5	347±39	0.49±0.25
**G2-paco**	1	262.3±9.4	0.77±0.36
DNA / **G2-paco**	20/500	148±11.3	0.26±0.03
DNA / **G2-paco**	20/100	1373±560	0.54±0.04
DNA / **G2-paco**	20/50	166±12	0.25±0.01
DNA / **G2-paco**	20/10	1763±176	0.35±0.06
DNA / **G2-paco**	20/5	1184±176	0.45±0.05
DNA / **G2-paco**	20/1	404±23	0.29±0.02
**G2-alt**	500	140±11	0.55±0.07
**G2-alt**	100	189±22	0.57±0.1
**G2-alt**	50	132.2±32.7	0.46±0.12
**G2-alt**	10	206±81.3	0.4±0.08
**G2-alt**	5	315.3±5.8	0.46±0.13
**G2-alt**	1	325±37.3	0.42±0.07
DNA / **G2-alt**	20/500	206±11.1	0.20±0.04
DNA / **G2-alt**	20/100	326.3±38.7	0.36±0.04
DNA / **G2-alt**	20/50	408.5±26	0.38±0.07
DNA / **G2-alt**	20/10	761±96.4	0.35±0.14
DNA / **G2-alt**	20/5	788.4±96	0.41±0.12
DNA / **G2-alt**	20/1	643±66.2	0.36±0.05

Table S3. Characteristics of light scattering (average hydrodynamic diameter of associates *d*, polydispersity index PDI) of the particles obtained by **G3 PAMAM-calix-dendrimers** and calf thymus DNA in a buffer solution (Tris-HCl buffer, 10 mM, pH 7.4).

System	C, μM	d, nm	PDI
calf thymus DNA	20	450±88.4	0.49±0.12
calf thymus DNA	10	542±200	0.53±0.18
**G3-cone**	10	542±254	0.61±0.16
**G3-cone**	5	988±242	0.86±0.11
**G3-cone**	2	863±363	0.76±0.15
DNA / **G3-cone**	20/0.16	281±11	0.26±0.02
DNA / **G3-cone**	20/0.32	490±38	0.38±0.04
DNA / **G3-cone**	20/1	263±34	0.31±0.01
DNA / **G3-cone**	20/2	182±2	0.16±0.01
DNA / **G3-cone**	10/1	171±12	0.25±0.01
DNA / **G3-cone**	10/2	102±1	0.17±0.01
DNA / **G3-cone**	10/3.33	122±2	0.18±0.01
DNA / **G3-cone**	10/5	138±8	0.22±0.02
DNA / **G3-cone**	10/10	122±2	0.21±0.02
DNA / **G3-cone**	10/0.16	390±23	0.26±0.04
DNA / **G3-cone**	10/0.32	819±56	0.48±0.05
**G3-alt**	10	623±305	0.67±0.17
**G3-alt**	5	762±452	0.74±0.19
**G3-alt**	2	610±239	0.72±0.12
DNA / **G3-alt**	20/2	202±43	0.30±0.03
DNA / **G3-alt**	20/1	532±69	0.42±0.05
DNA / **G3-alt**	20/0.32	268±8	0.35±0.01
DNA / **G3-alt**	20/0.16	303±32	0.32±0.05
DNA / **G3-alt**	10/0.16	312±15	0.26±0.01
DNA / **G3-alt**	10/0.32	260±34	0.28±0.03
DNA / **G3-alt**	10/1	219±36	0.33±0.03
DNA / **G3-alt**	10/10	244±17	0.36±0.01
DNA / **G3-alt**	10/2	147±2	0.25±0.01
DNA / **G3-alt**	10/3.33	119±1	0.15±0.01
DNA / **G3-alt**	10/5	106±1	0.17±0.01
**G3-paco**	10	774±460	0.73±0.20
**G3-paco**	5	712±187	0.70±0.10
**G3-paco**	2	610±239	0.72±0.12
DNA / **G3-paco**	20/0.16	244±19	0.27±0.01
DNA / **G3-paco**	20/0.32	265±9	0.34±0.03
DNA / **G3-paco**	20/1	221±39	0.30±0.02
DNA / **G3-paco**	20/2	185±2	0.20±0.01
DNA / **G3-paco**	10/0.16	245±15	0.26±0.01
DNA / **G3-paco**	10/0.32	245±15	0.27±0.02
DNA / **G3-paco**	10/2	123±1	0.20±0.01
DNA / **G3-paco**	10/3.33	185±9	0.23±0.02
DNA / **G3-paco**	10/5	151±8	0.20±0.01
DNA / **G3-paco**	10/1	165±8	0.17±0.01
DNA / **G3-paco**	10/10	173±9	0.22±0.01

Table S4. Electrokinetic potentials (ζ-potentials) of **G3 PAMAM-calix-dendrimers**, calf thymus DNA (10 μM in nucleotide base pairs) and their complexes in a buffer solution (Tris-HCl buffer, 10 mM, pH 7.4).

System	C, μM	ζ-potential, mV
calf thymus DNA	10	–31.3±7.8
**G3-cone**	10	+17.8±3.9
DNA / **G3-cone**	10/1	–20.0±2.3
DNA / **G3-cone**	10/2	+33.3±0.9
DNA / **G3-cone**	10/10	+40.0±1.1
**G3-alt**	10	+13.1±1.3
DNA / **G3-alt**	10/1	–28.3±1.5
DNA / **G3-alt**	10/2	+30.5±1.3
DNA / **G3-alt**	10/3.33	+37.1±1.7
**G3-paco**	10	+10.2±5.0
DNA / **G3-paco**	10/1	–19.8±0.9
DNA / **G3-paco**	10/10	+38.4±1.3

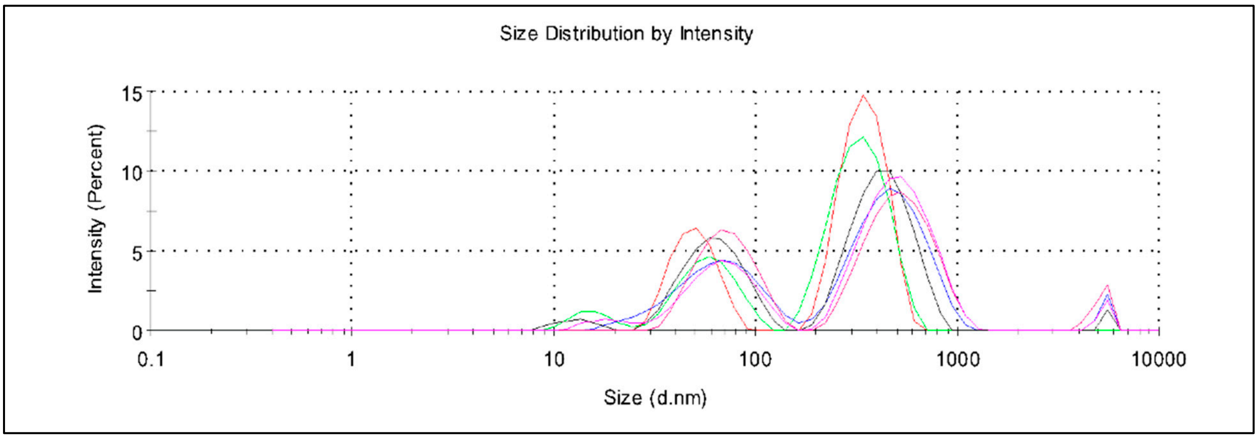

Figure S56. Size distribution (by intensity) of calf thymus DNA (20 μM in nucleotide base pairs) associates (10 mM Tris-HCl, pH = 7.4).

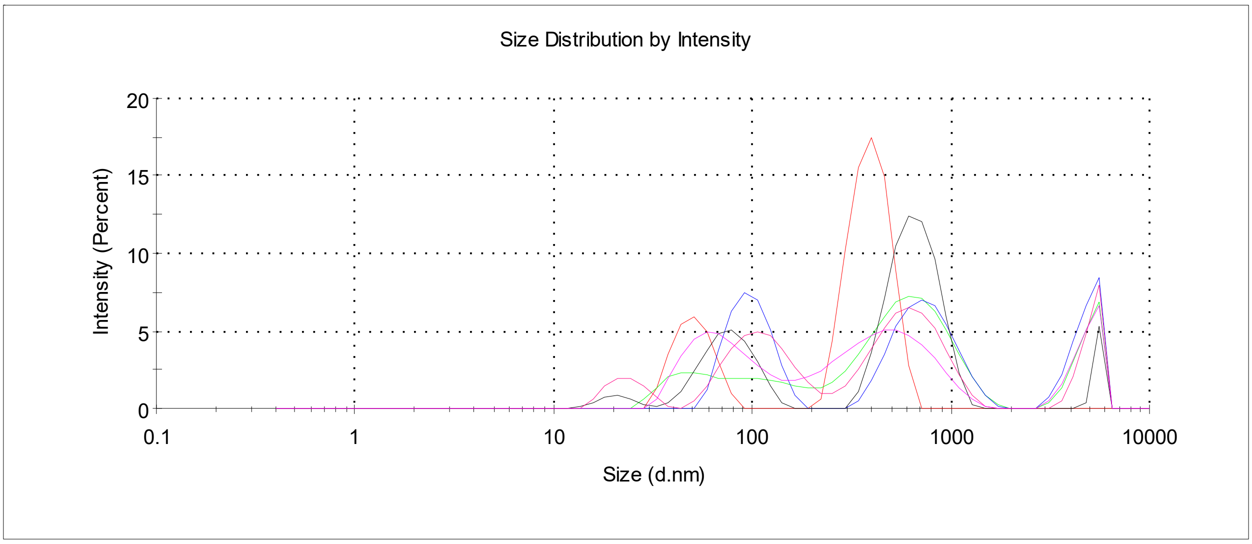

Figure S57. Size distribution (by intensity) of calf thymus DNA (10 μM in nucleotide base pairs) associates (10 mM Tris-HCl, pH = 7.4).

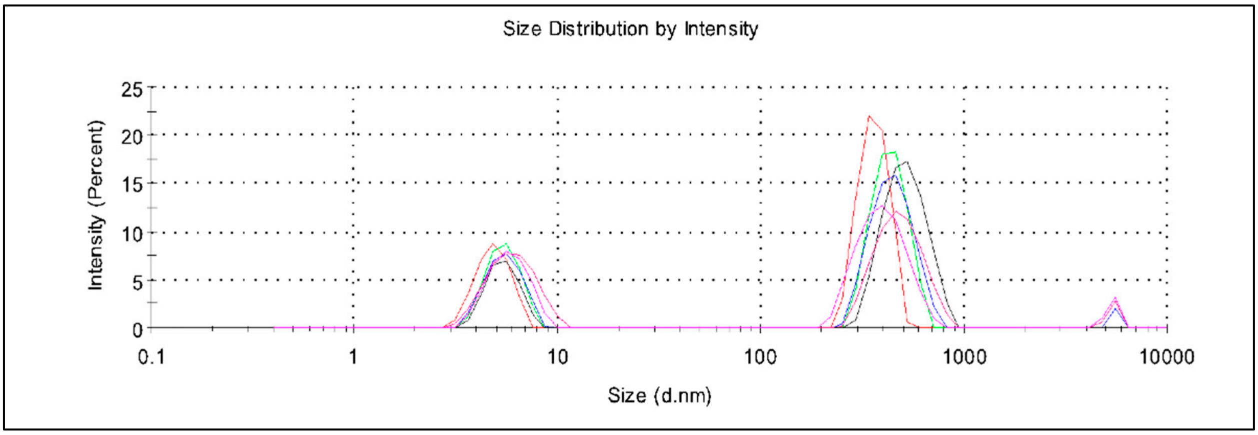

Figure S58. Size distribution (by intensity) of **G1-cone** associates (50 μM) (10 mM Tris-HCl, pH = 7.4).

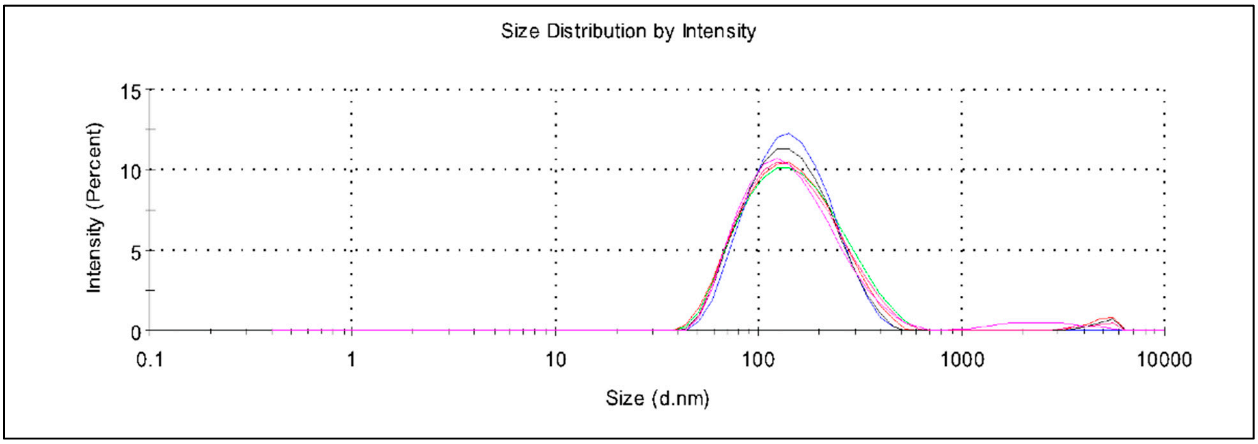

Figure S59. Size distribution (by intensity) of **G1-cone** (50 μM) / calf thymus DNA (20 mM in nucleotide base pairs) associates (10 mM Tris-HCl, pH = 7.4).

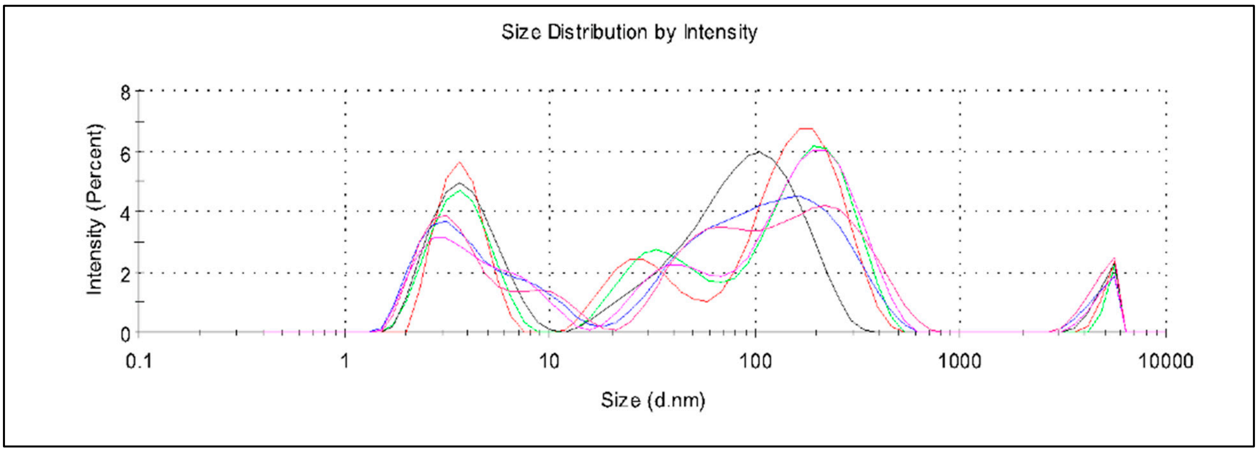

Figure S60. Size distribution (by intensity) of **G1-paco** (500 μM) associates (10 mM Tris-HCl, pH = 7.4).

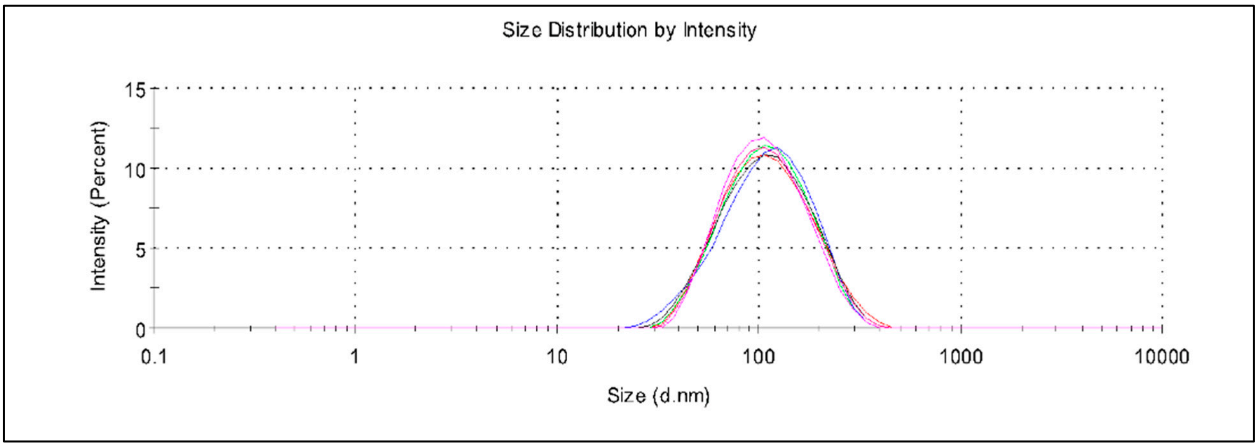

Figure S61. Size distribution (by intensity) of **G1-paco** (500 μM) / calf thymus DNA (20 μM in nucleotide base pairs) associates (10 mM Tris-HCl, pH = 7.4).

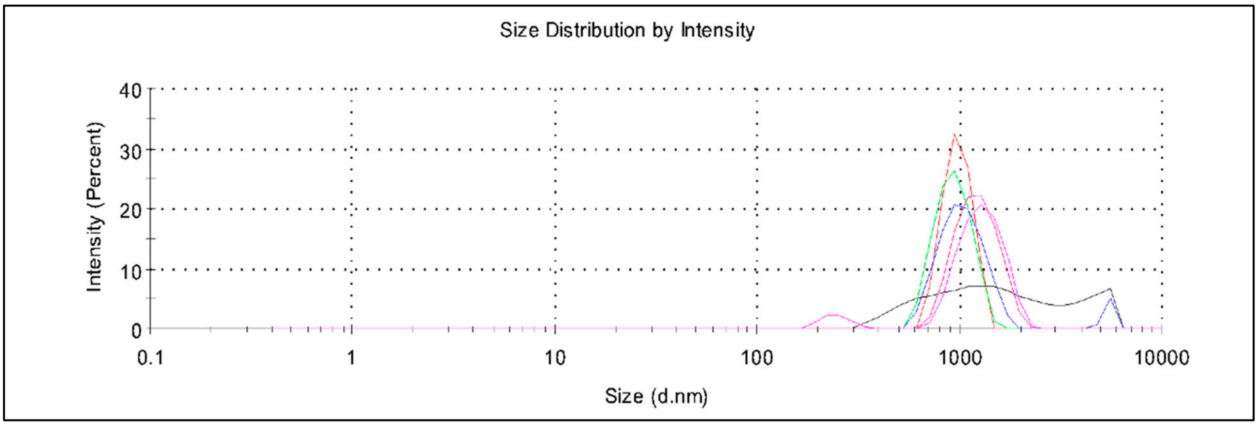

Figure S62. Size distribution (by intensity) of **G1-alt** (10 μM) associates (10 mM Tris-HCl, pH = 7.4).

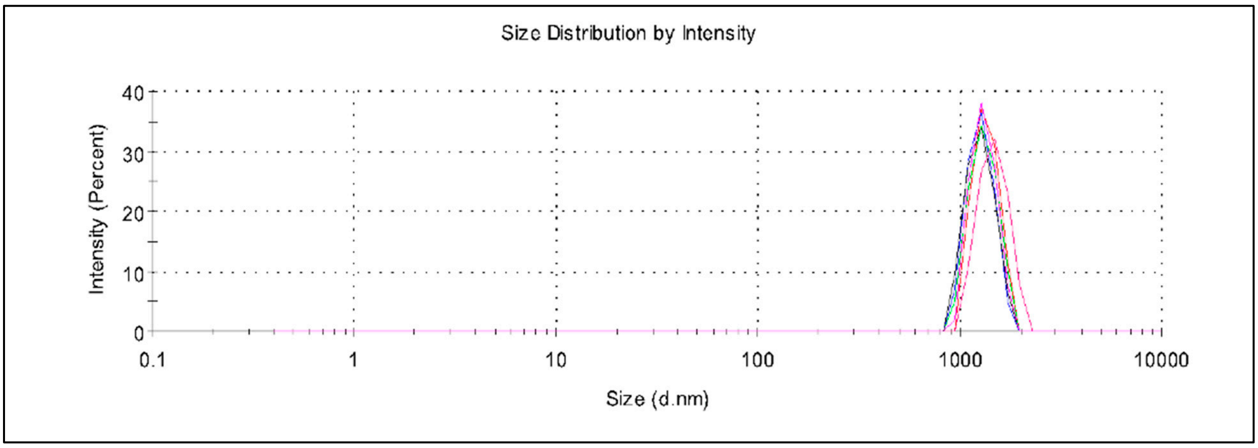

Figure S63. Size distribution (by intensity) of **G1-alt** (10 μM) / calf thymus DNA (20 μM in nucleotide base pairs) associates (10 mM Tris-HCl, pH = 7.4).

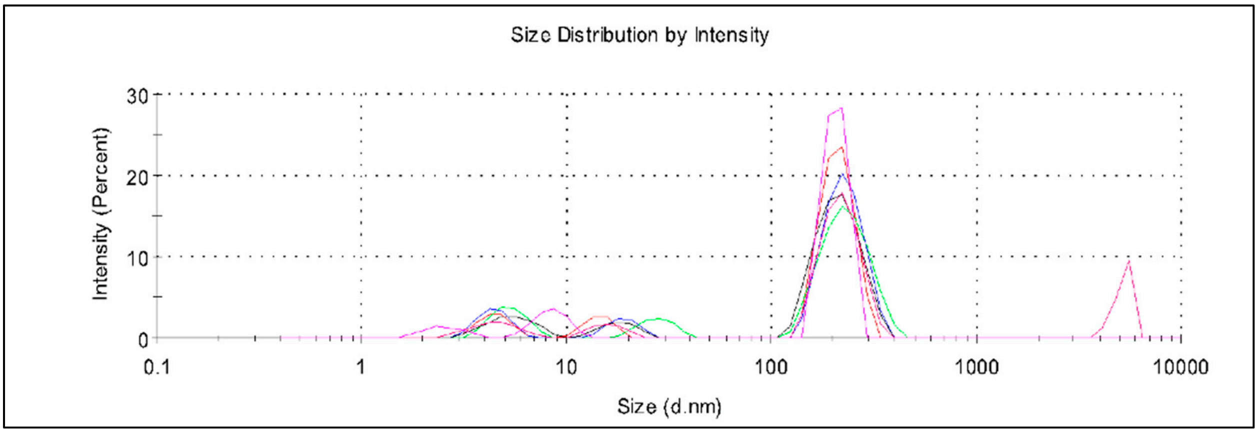

Figure S64. Size distribution (by intensity) of **G2-cone** (50 µM) associates (10 mM Tris-HCl, pH = 7.4).

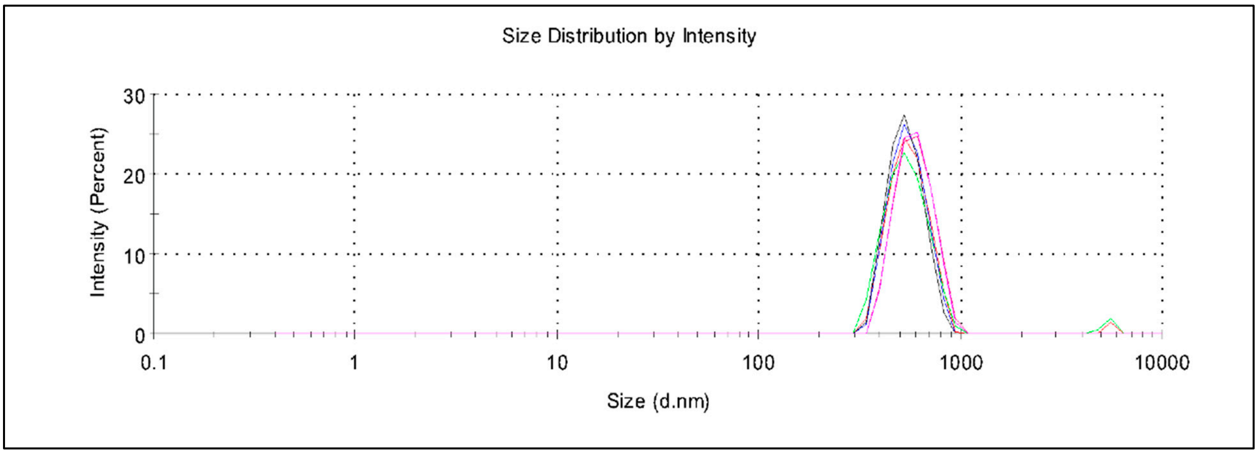

Figure S65. Size distribution (by intensity) of **G2-cone** (50 µM) / calf thymus DNA (20 µM in nucleotide base pairs) associates (10 mM Tris-HCl, pH = 7.4).

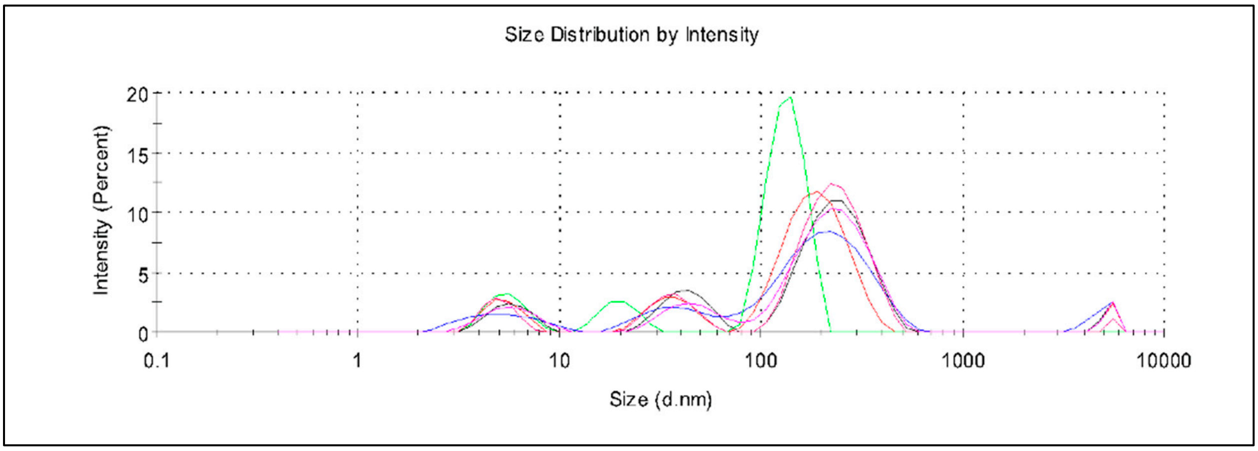

Figure S66. Size distribution (by intensity) of **G2-paco** (50 µM) associates (10 mM Tris-HCl, pH = 7.4).

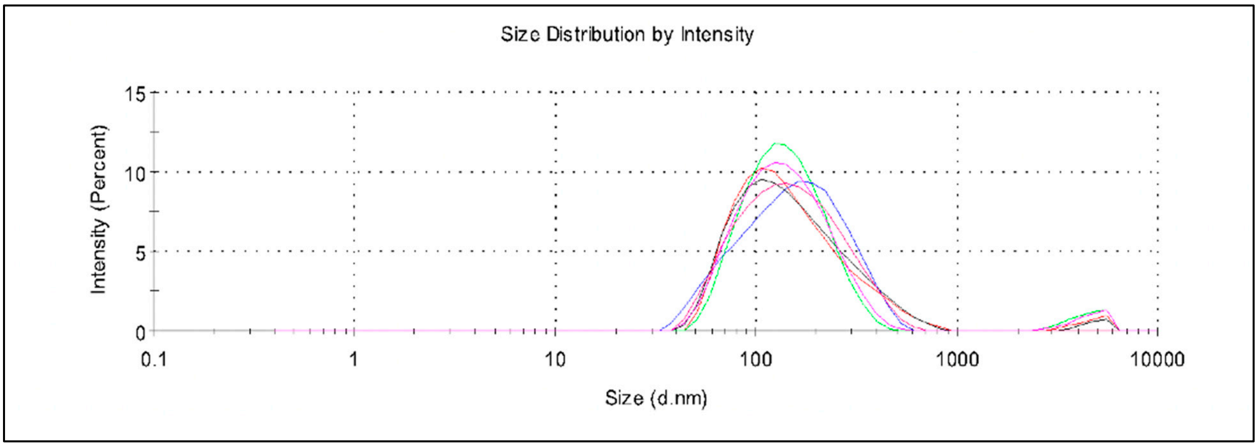

Figure S67. Size distribution (by intensity) of **G2-paco** (50 μM) / calf thymus DNA (20 μM in nucleotide base pairs) associates (10 mM Tris-HCl, pH = 7.4).

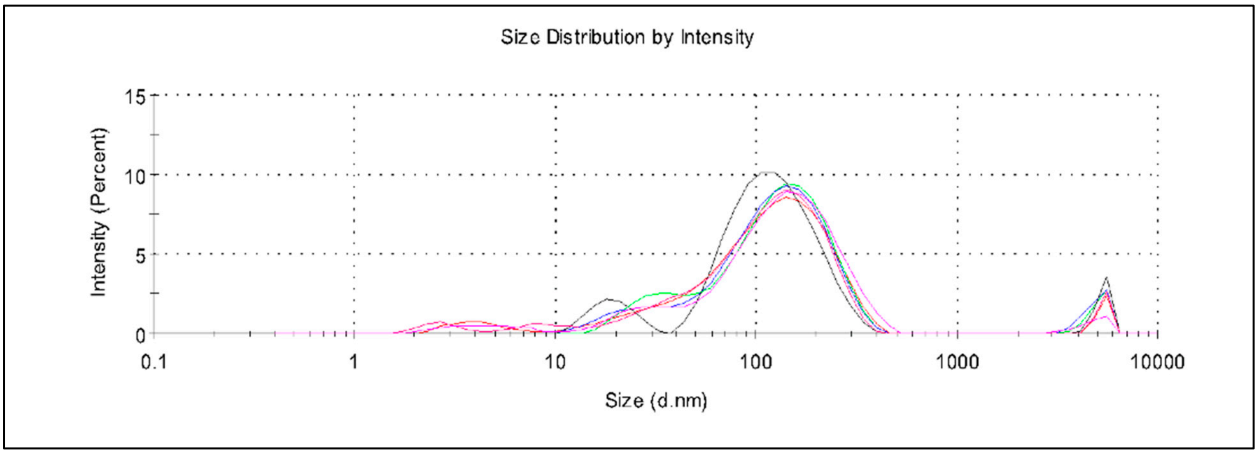

Figure S68. Size distribution (by intensity) of **G2-alt** (500 μM) associates (10 mM Tris-HCl, pH = 7.4).

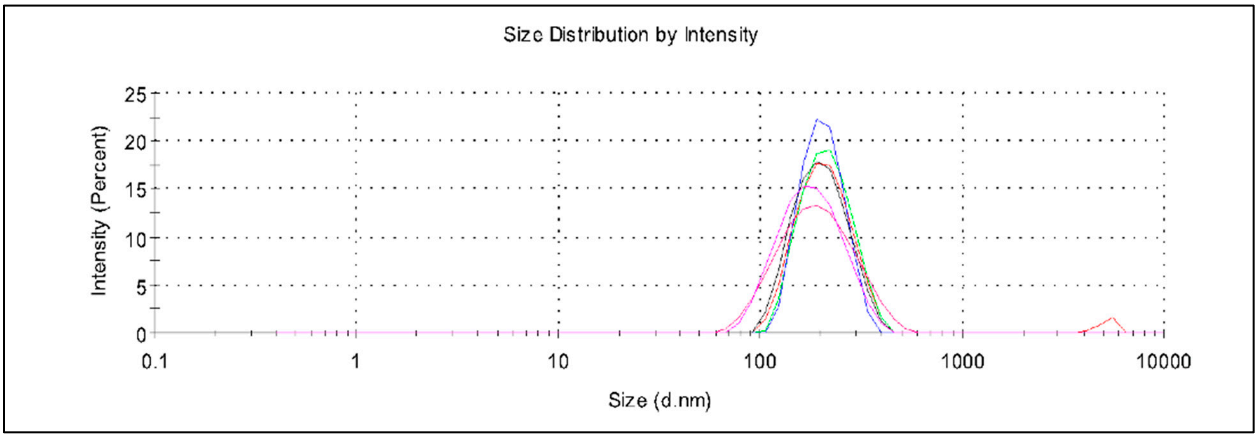

Figure S69. Size distribution (by intensity) of **G2-alt** (500 μM) / calf thymus DNA (20 μM in nucleotide base pairs) associates (10 mM Tris-HCl, pH = 7.4).

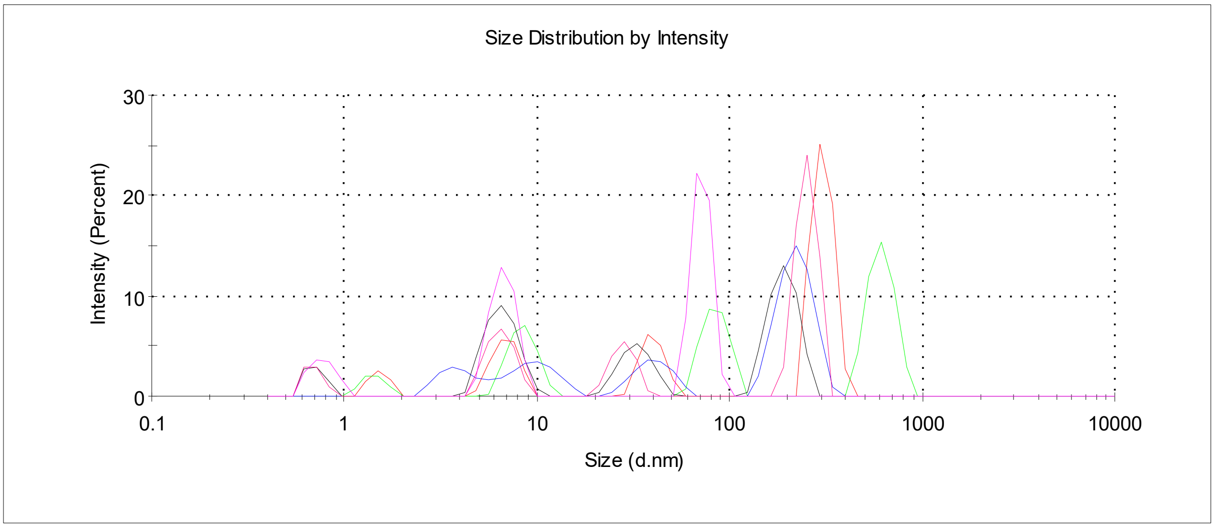

Figure S70. Size distribution (by intensity) of **G3-cone** (2 μM) associates (10 mM Tris-HCl, pH = 7.4).

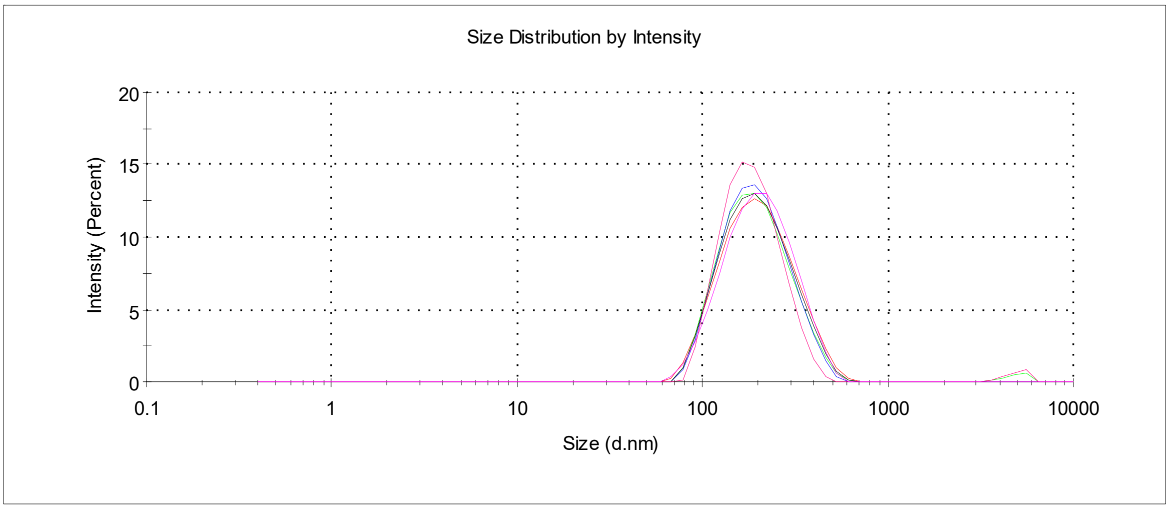

Figure S71. Size distribution (by intensity) of **G3-cone** (2 μM) / calf thymus DNA (20 μM in nucleotide base pairs) associates (10 mM Tris-HCl, pH = 7.4).

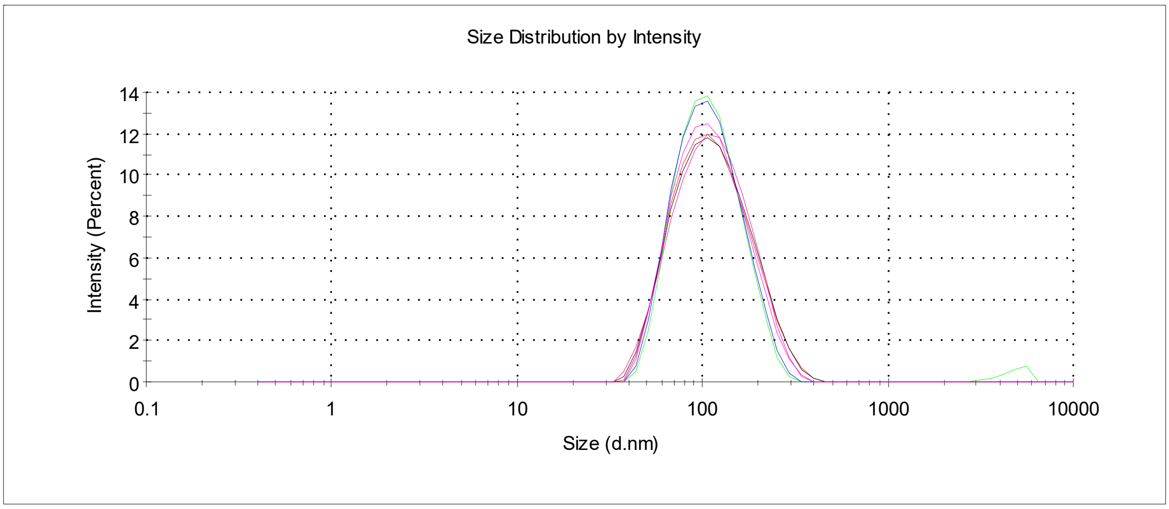

Figure S72. Size distribution (by intensity) of **G3-cone** (2 μM) / calf thymus DNA (10 μM in nucleotide base pairs) associates (10 mM Tris-HCl, pH = 7.4).

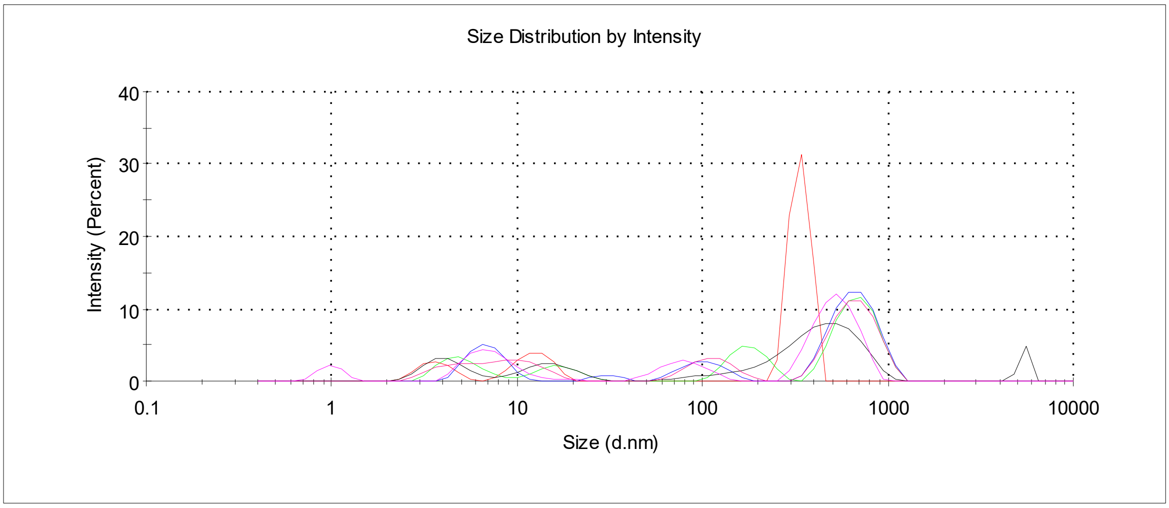

Figure S73. Size distribution (by intensity) of **G3-paco** (2 μM) associates (10 mM Tris-HCl, pH = 7.4).

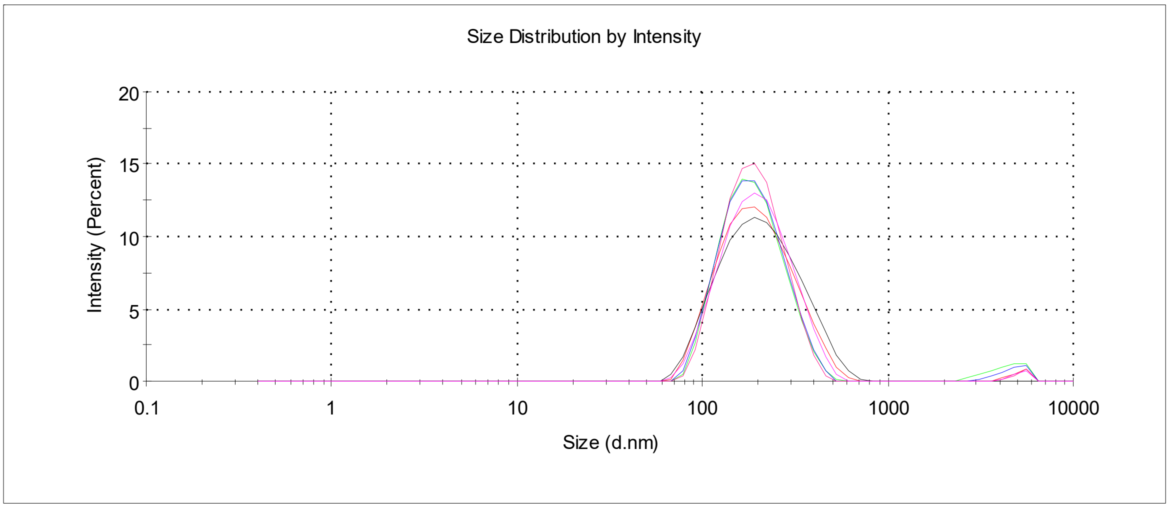

Figure S74. Size distribution (by intensity) of **G3-paco** (2 μM) / calf thymus DNA (20 μM in nucleotide base pairs) associates (10 mM Tris-HCl, pH = 7.4).

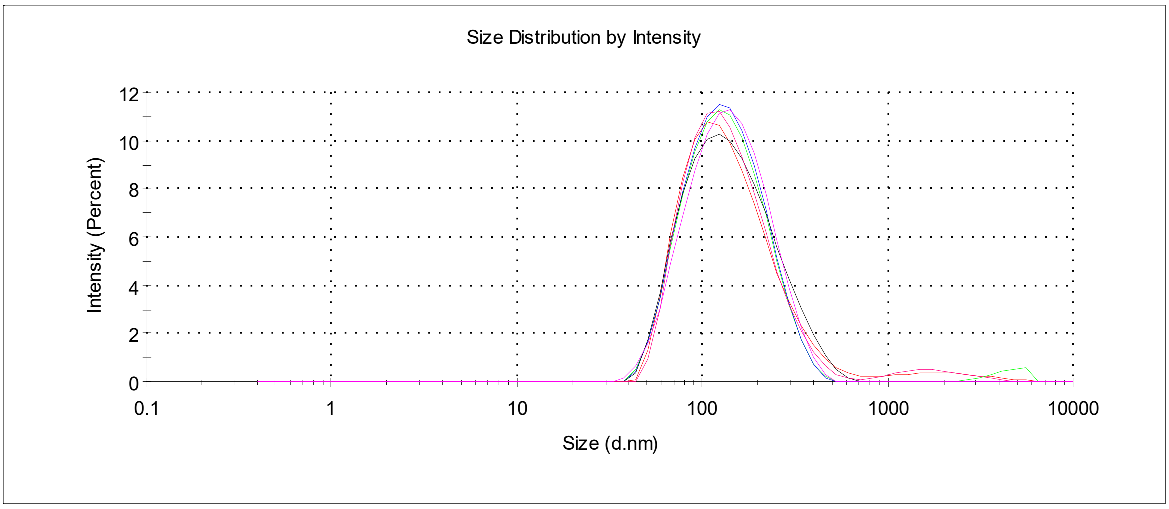

Figure S75. Size distribution (by intensity) of **G3-paco** (2 μM) / calf thymus DNA (10 μM in nucleotide base pairs) associates (10 mM Tris-HCl, pH = 7.4).

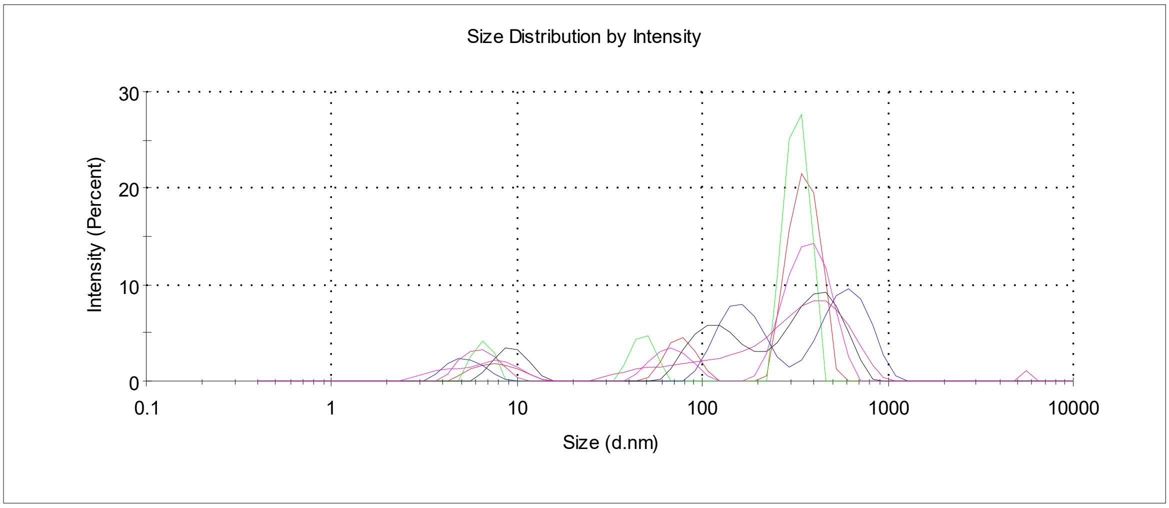

Figure S76. Size distribution (by intensity) of **G3-alt** (5 μM) associates (10 mM Tris-HCl, pH = 7.4).

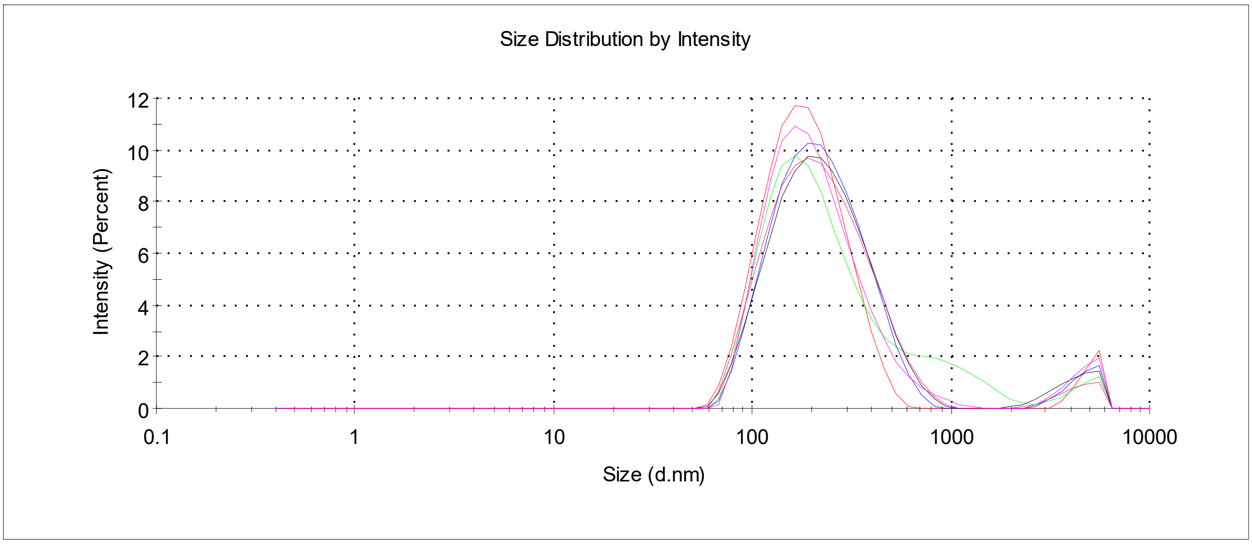

Figure S77. Size distribution (by intensity) of **G3-alt** (2 μM) / calf thymus DNA (20 μM in nucleotide base pairs) associates (10 mM Tris-HCl, pH = 7.4).

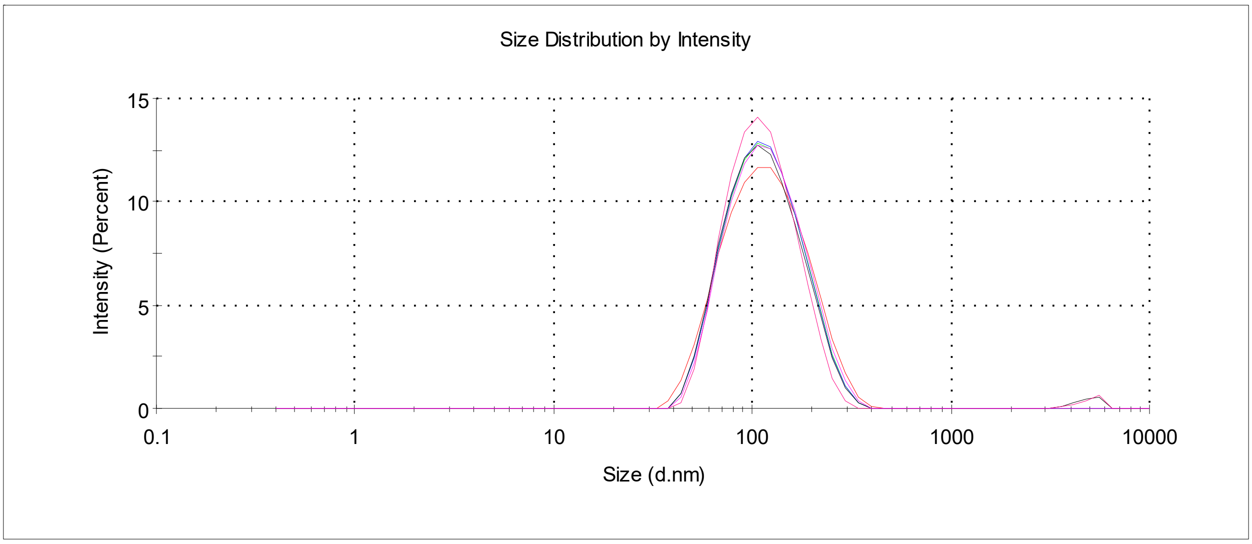

Figure S78. Size distribution (by intensity) of **G3-alt** (5 μM) / calf thymus DNA (10 μM in nucleotide base pairs) associates (10 mM Tris-HCl, pH = 7.4).

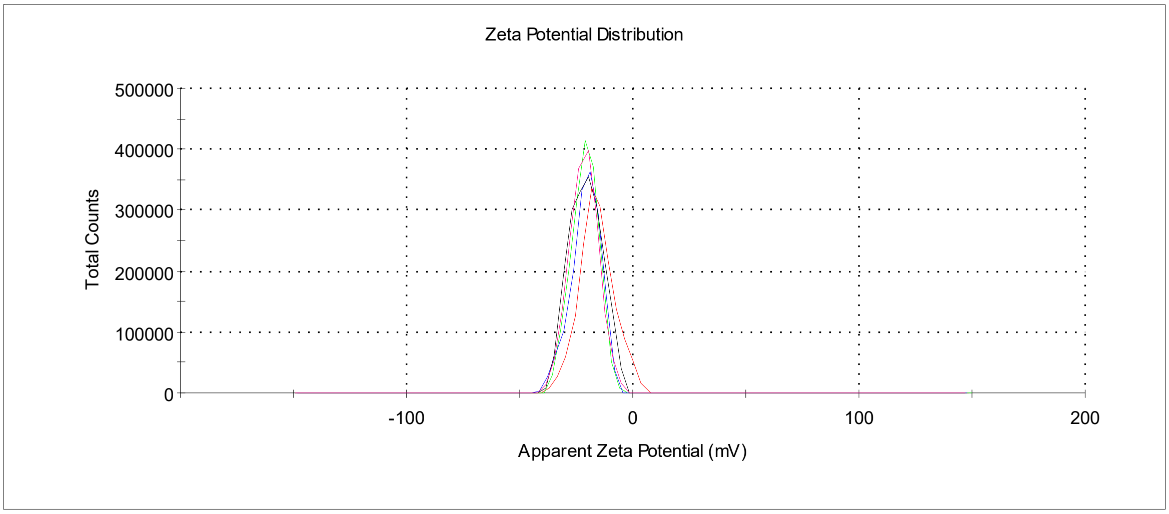

Figure S79. Zeta-potential distributions of **G3-cone** (1 μM) / calf thymus DNA (10 μM in nucleotide base pairs) associates (10 mM Tris-HCl, pH = 7.4).

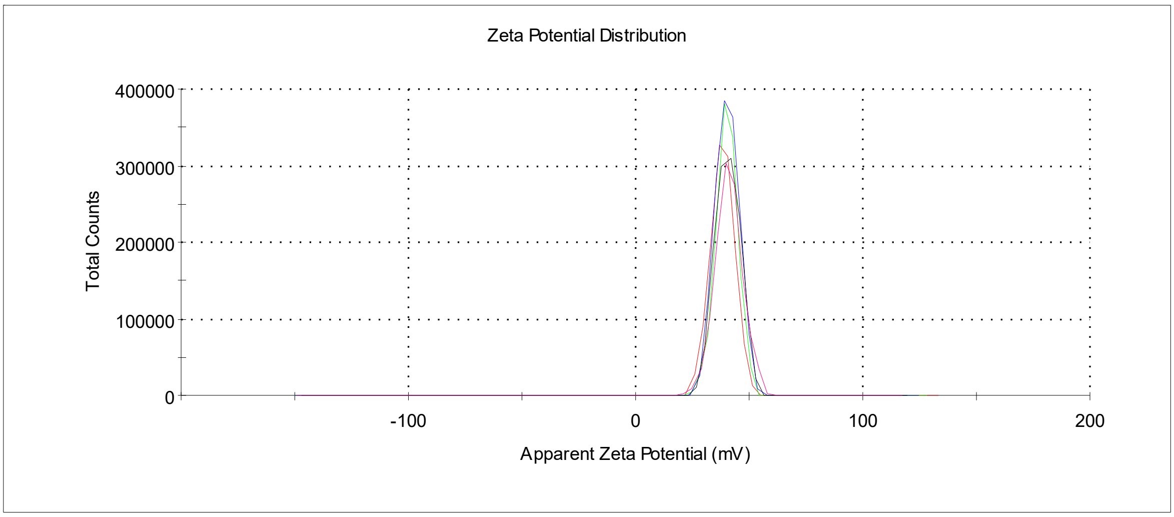

Figure S80. Zeta-potential distributions of **G3-cone** (2 μM) / calf thymus DNA (10 μM in nucleotide base pairs) associates (10 mM Tris-HCl, pH = 7.4).

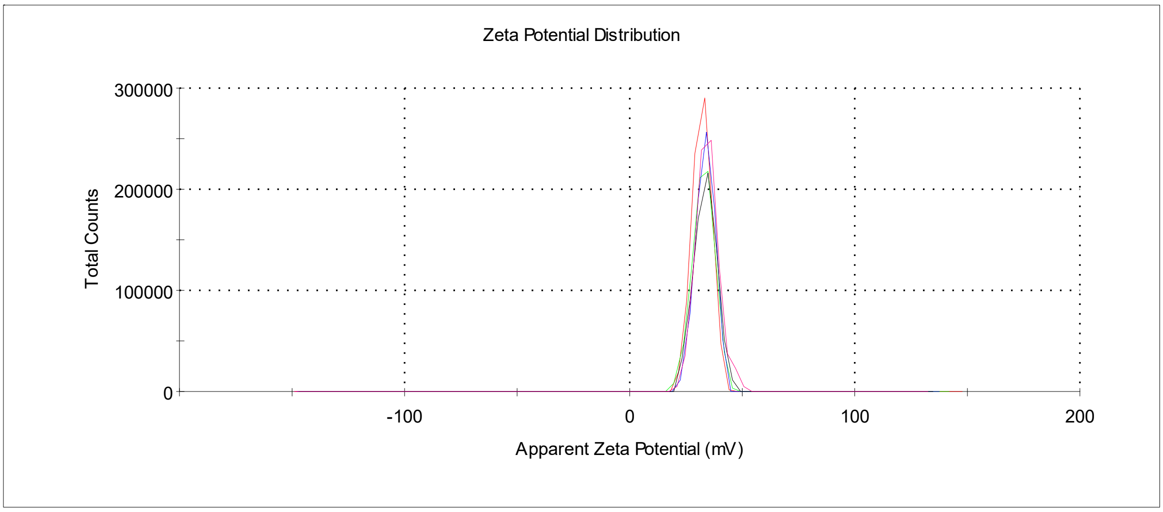

Figure S81. Zeta-potential distributions of **G3-cone** (10 μM) / calf thymus DNA (10 μM in nucleotide base pairs) associates (10 mM Tris-HCl, pH = 7.4).

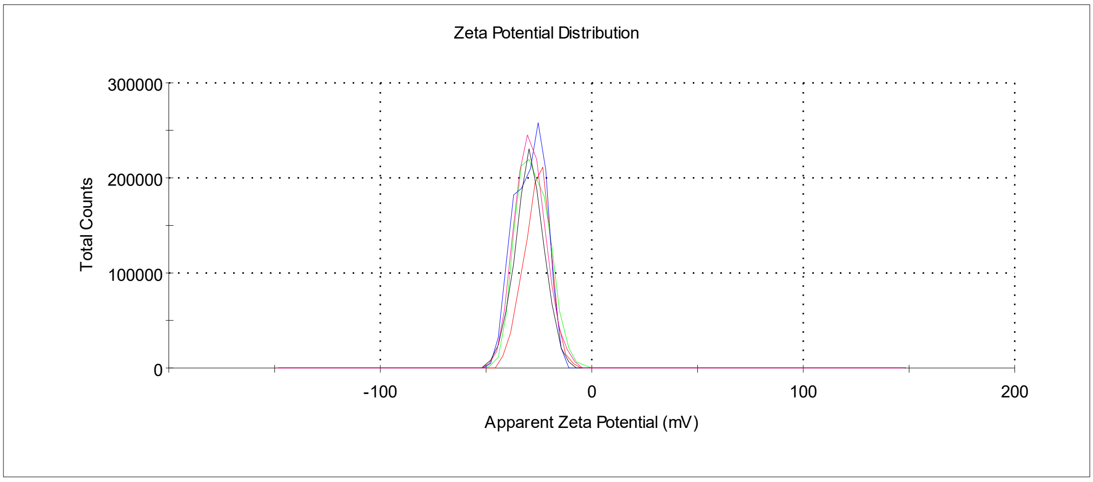

Figure S82. Zeta-potential distributions of **G3-alt** (1 μM) / calf thymus DNA (10 μM in nucleotide base pairs) associates (10 mM Tris-HCl, pH = 7.4).

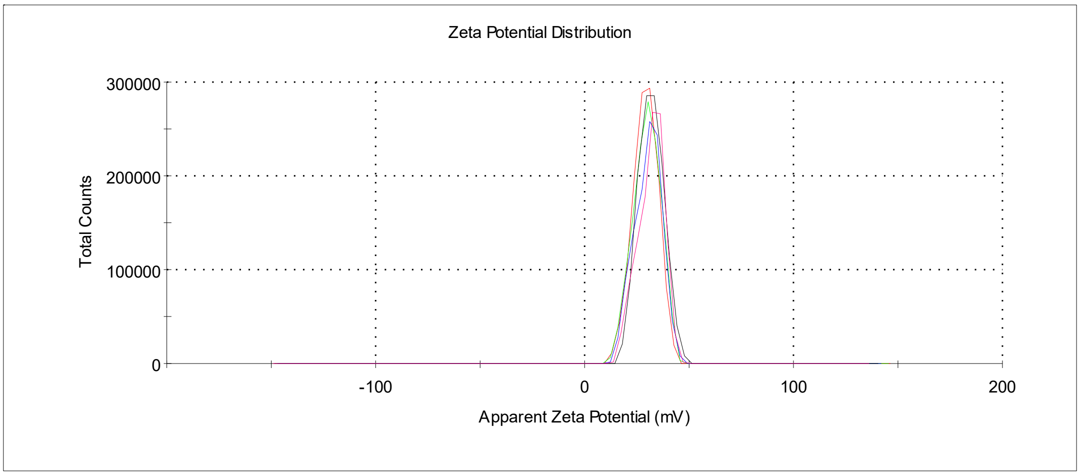

Figure S83. Zeta-potential distributions of **G3-alt** (2 μM) / calf thymus DNA (10 μM in nucleotide base pairs) associates (10 mM Tris-HCl, pH = 7.4).

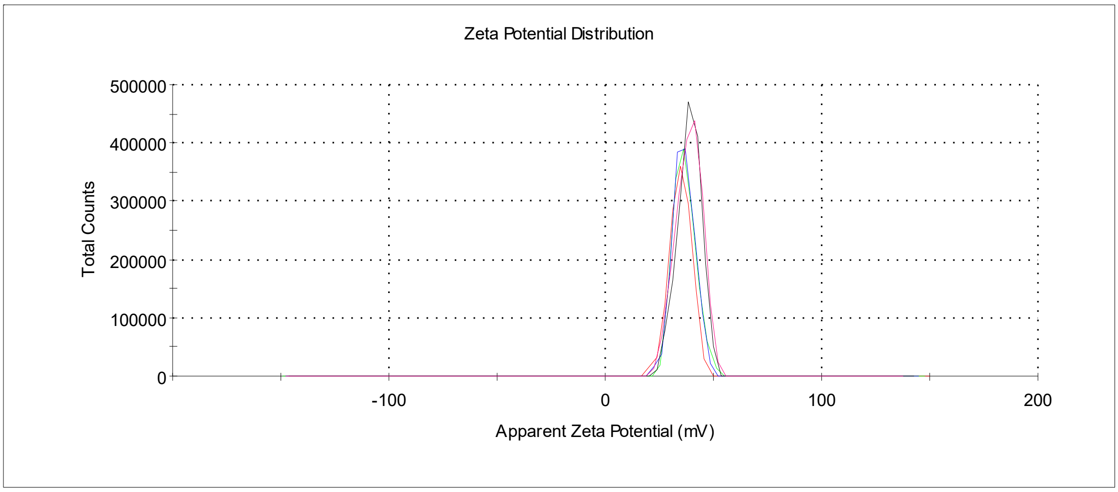

Figure S84. Zeta-potential distributions of **G3-alt** (10 μM) / calf thymus DNA (10 μM in nucleotide base pairs) associates (10 mM Tris-HCl, pH = 7.4).

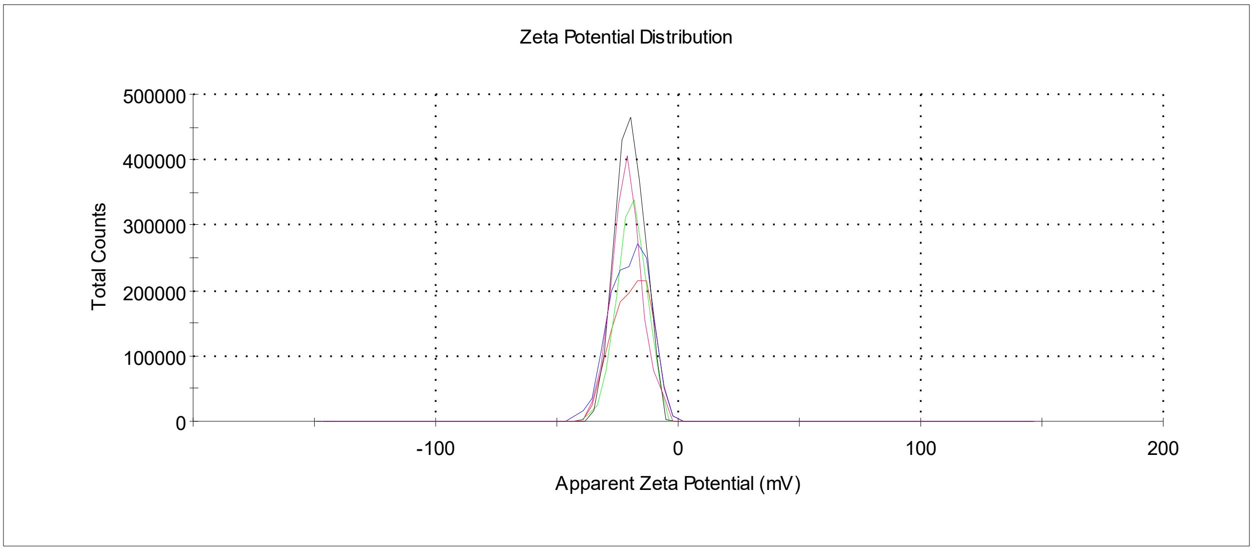

Figure S85. Zeta-potential distributions of **G3-paco** (1 μM) / calf thymus DNA (10 μM in nucleotide base pairs) associates (10 mM Tris-HCl, pH = 7.4).

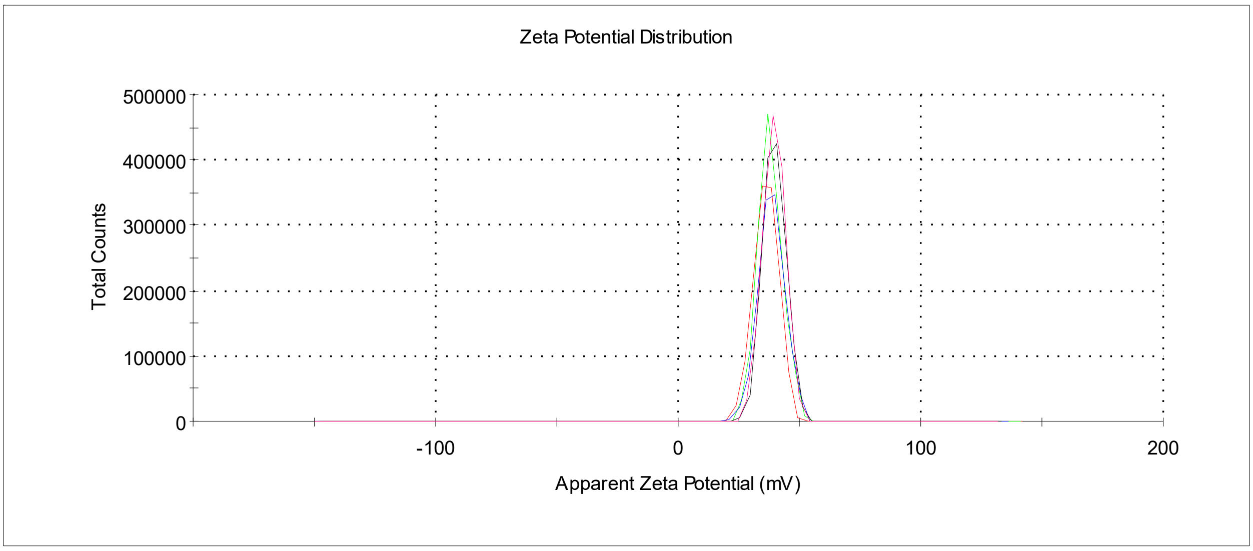

Figure S86. Zeta-potential distributions of **G3-paco** (10 μM) / calf thymus DNA (10 μM in nucleotide base pairs) associates (10 mM Tris-HCl, pH = 7.4).

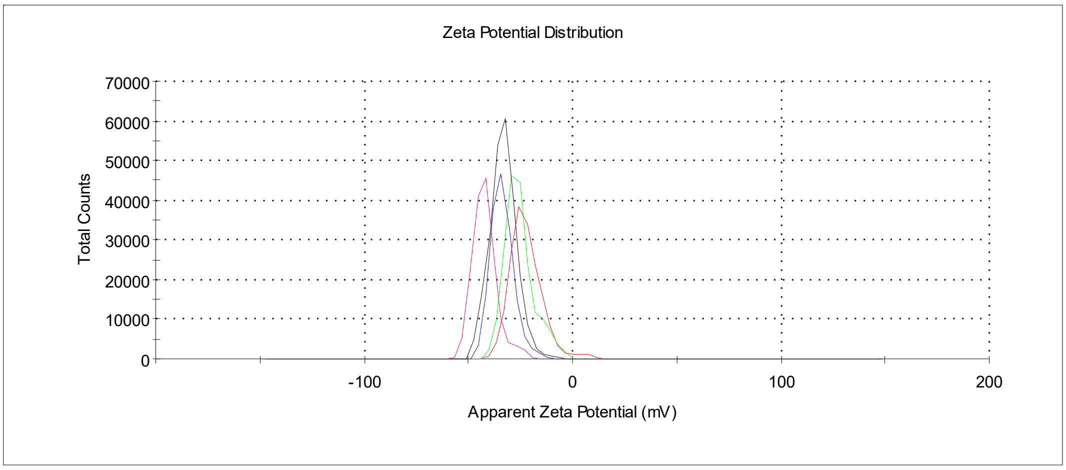

Figure S87. Zeta-potential distributions of calf thymus DNA (10 μM in nucleotide base pairs) associates (10 mM Tris-HCl, pH = 7.4).

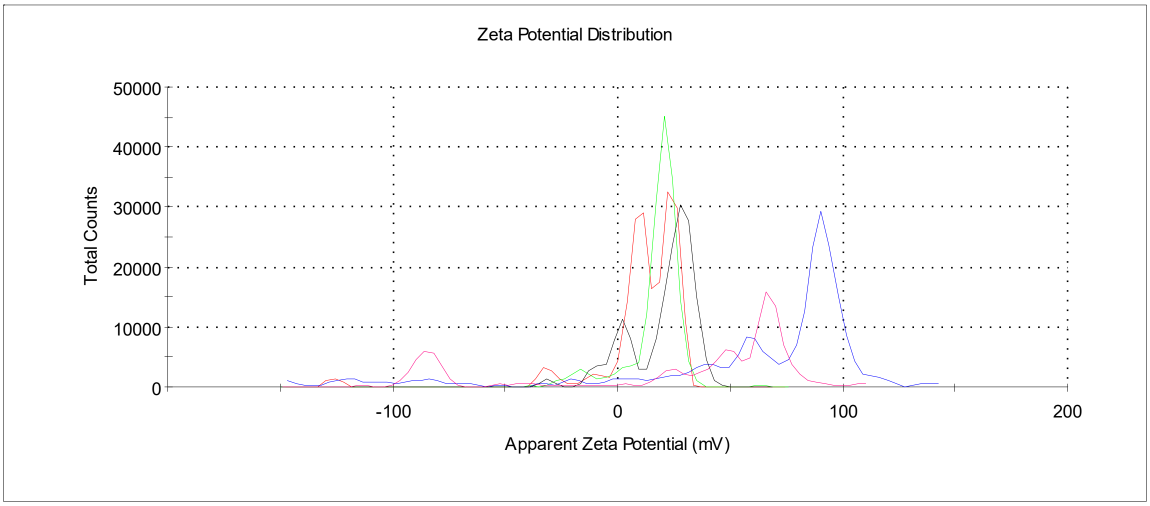

Figure S88. Zeta-potential distributions of **G3-cone** (10 μM) associates (10 mM Tris-HCl, pH = 7.4).

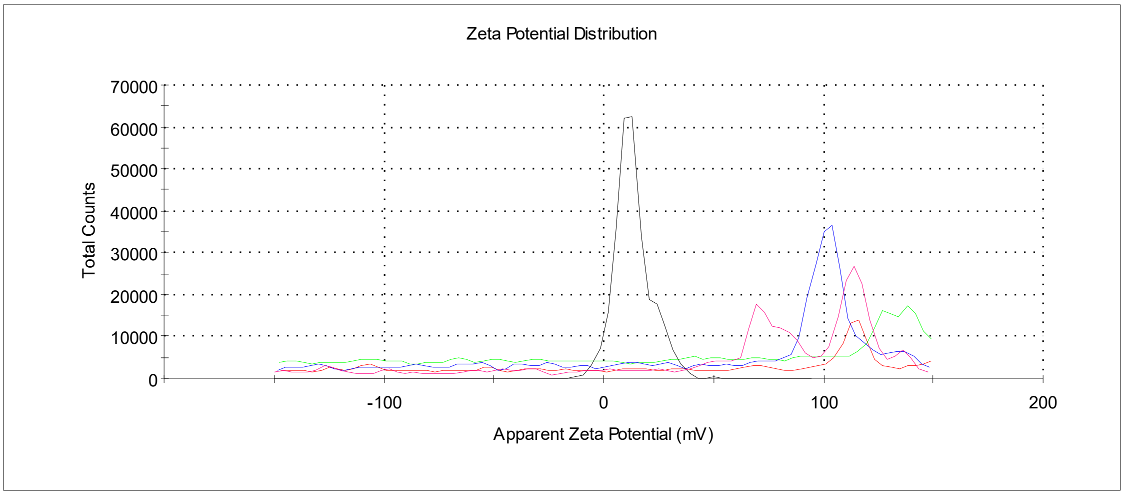

Figure S89. Zeta-potential distributions of **G3-paco** (10 μM) associates (10 mM Tris-HCl, pH = 7.4).

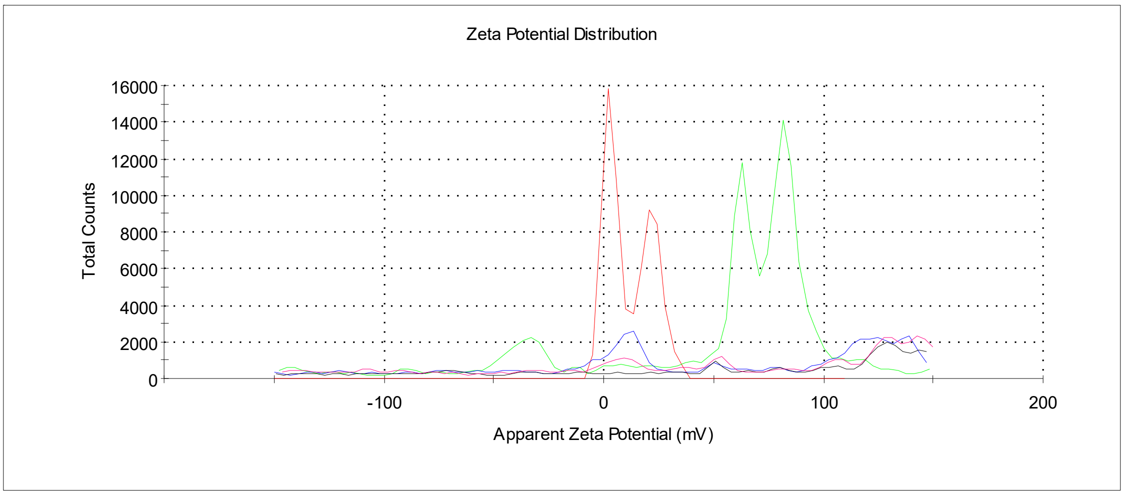

Figure S90. Zeta-potential distributions of **G3-alt** (10 μM) associates (10 mM Tris-HCl, pH = 7.4).

3.4. TEM data

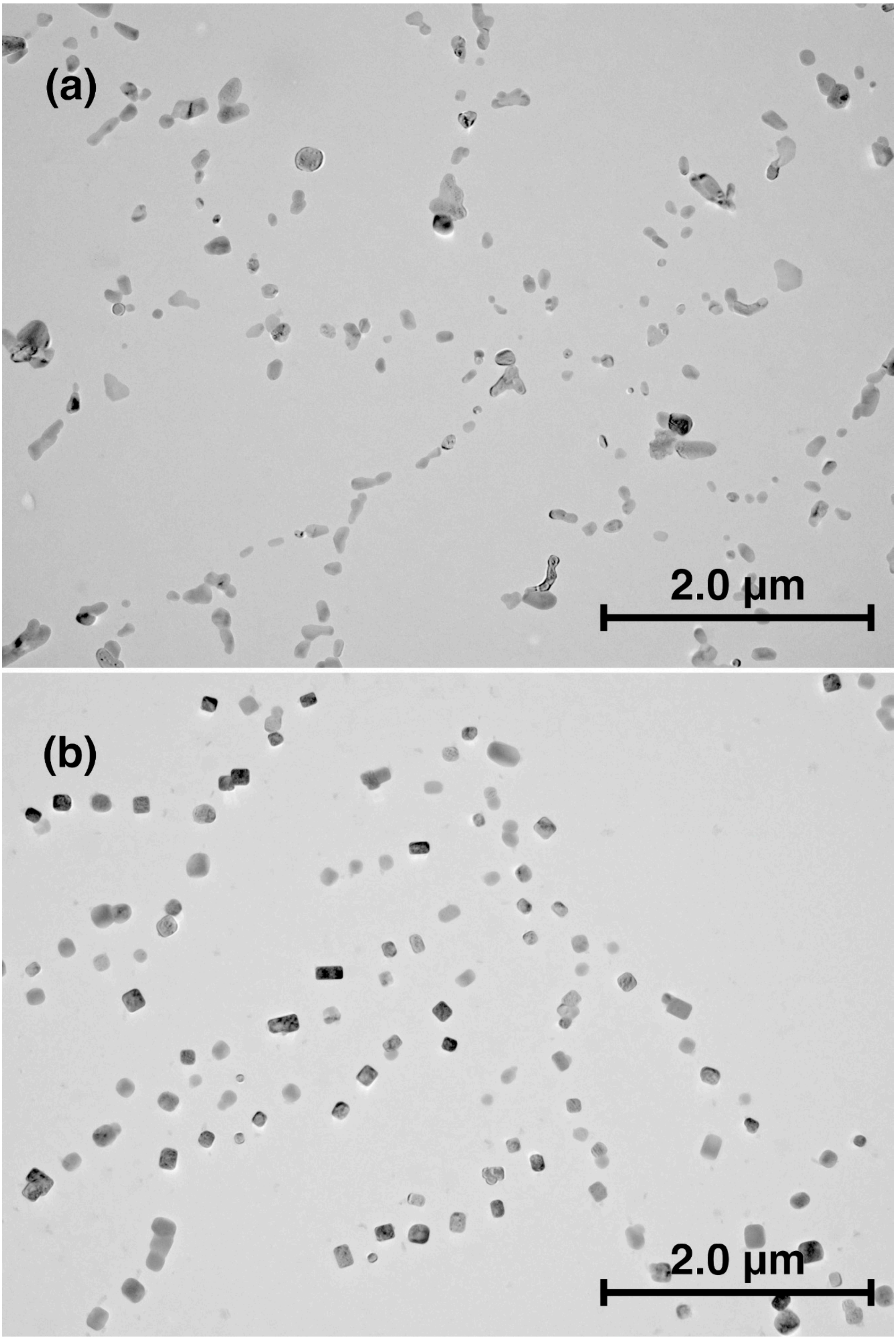

Figure S91. TEM images of (a) **G3-paco** (1 μM); (b) DNA (0.6 μM in nucleotide base pairs) with **G3-paco** (1 μM).

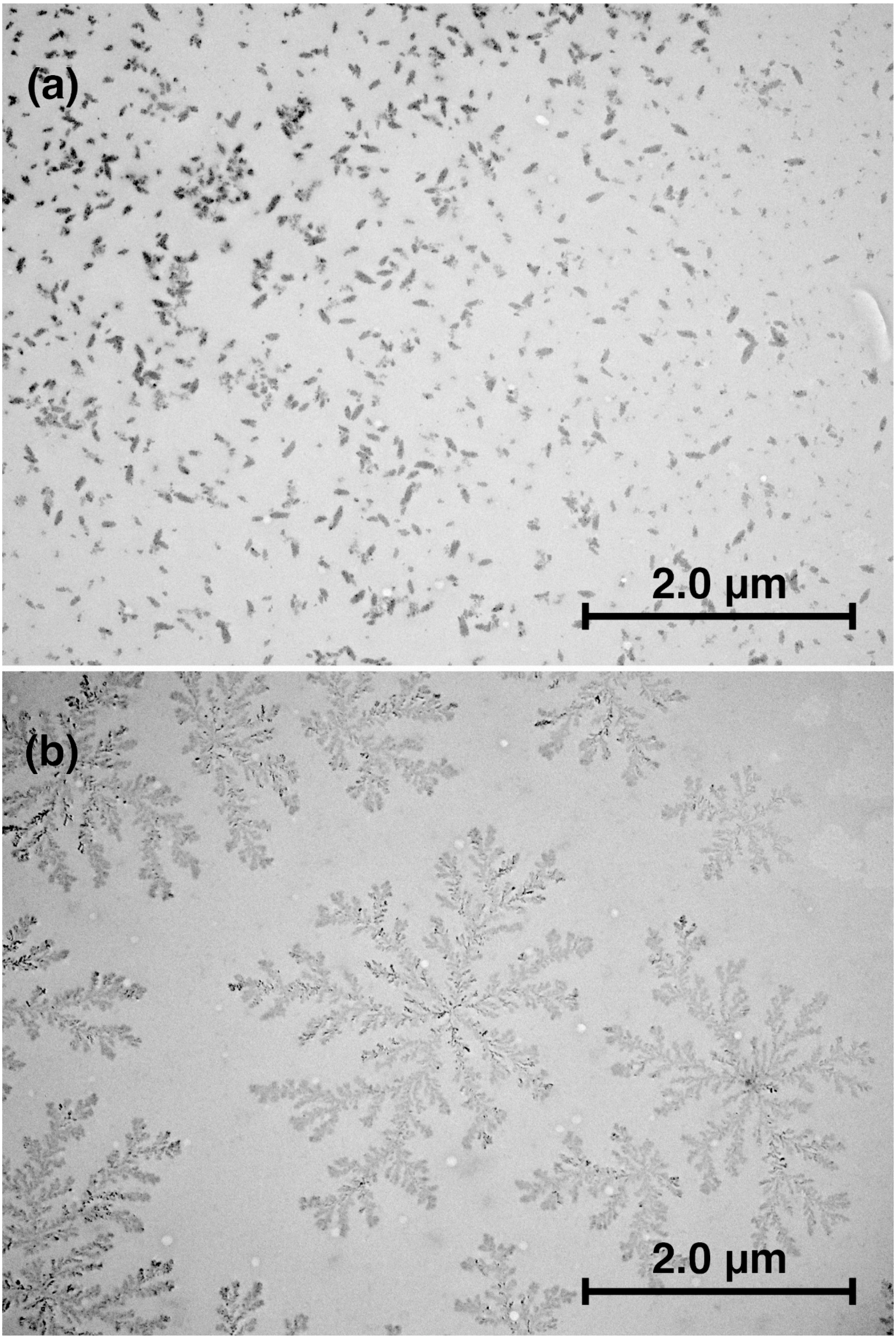

Figure S92. TEM images of (a) **G3-alt** (1 μM); (b) DNA (0.6 μM in nucleotide base pairs) with **G3-alt** (1 μM).

4. References

[1] O. Mostovaya, I. Shiabiev, D. Pysin, A. Stanavaya, V. Abashkin, D. Shcharbin, P. Padnya, I. Stoikov, PAMAM-calix-dendrimers: second generation synthesis, fluorescent properties and catecholamines binding, Pharmaceutics 14 (2022) 2748, https://doi.org/10.3390/pharmaceutics14122748.

[2] O. Mostovaya, P. Padnya, I. Shiabiev, T. Mukhametzyanov, I. Stoikov, PAMAM-calix-dendrimers: synthesis and thiacalixarene conformation effect on DNA binding, Int. J. Mol. Sci. 22 (2021) 11901, https://doi.org/10.3390/ijms222111901.
